# Effects of Combined Diet and Physical Activity on Gestational Weight Gain in Low-Risk Pregnant Women Based on the TIDieR Checklist: A Systematic Review and Meta-Analysis

**DOI:** 10.3390/healthcare14081035

**Published:** 2026-04-14

**Authors:** Wenjing Luo, Haishan Wei, Kaili Zhang, Dehui Wang, Hong Lu, Yinchu Hu, Chunying Li, Junrong Zhang, Xiu Zhu

**Affiliations:** 1 School of Nursing, Peking University, Beijing 100191, China; wenjingluo@bjmu.edu.cn (W.L.); 2411210138@bjmu.edu.cn (H.W.); yinchuhu@bjmu.edu.cn (Y.H.); zhuxiu@bjmu.edu.cn (X.Z.); 2 Department of Obstetrics and Gynaecology, Beijing Hospital, National Center of Gerontology, Institute of Geriatric Medicine, Chinese Academy of Medical Sciences, Beijing 100730, China; zhangkaili3973@bjhmoh.cn (K.Z.); zhangjunrong3106@bjhmoh.cn (J.Z.); 3 Health Science Library, Peking University, Beijing 100191, China; leecy@bjmu.edu.cn

**Keywords:** gestational weight gain, normal pre-pregnancy BMI, diet, physical activity

## Abstract

**Highlights:**

**What are the main findings?**

- Combined diet and physical activity interventions are associated with reduced total gestational weight gain and lower risk of excessive gestational weight gain among low-risk pregnant women.
- Specific intervention features identified in subgroup analyses may be related to improved outcomes, including individual delivery formats, mixed face-to-face and remote modes, moderate frequency and healthcare-based implementation.

**What are the implications of the main findings?**

- Low-risk pregnant women remain an important population for gestational weight management, and structured combined diet and physical activity interventions may offer potential benefits.
- These findings may serve as preliminary evidence to inform the development and refinement of intervention strategies, while further research is needed to establish their effectiveness and applicability in routine clinical settings.

**Abstract:**

Background: While combined diet and physical activity interventions are recommended, evidence specific to low-risk pregnant women remains limited. As responses to combined interventions may vary by pre-pregnancy BMI, maternal health status and pregnancy outcomes, targeted evaluation in low-risk pregnant women is needed. Inconsistencies across studies, along with the lack of a comprehensive synthesis of both effects and intervention components, further limit their implementation. Objective: Our aims were to assess the effects of combined diet and physical activity interventions on gestational weight gain among low-risk women and to systematically characterize the intervention components. Design: We conducted a systematic review and meta-analysis following the *Cochrane Handbook* guidelines and PRISMA 2020. Methods: Eight databases and trial registries were searched from inception to 16 March 2026. Two reviewers independently conducted study selection, data extraction and risk of bias assessment. Intervention components were coded using the TIDieR checklist. The quality of included studies was assessed using the updated Cochrane risk of bias 2.0 tool. Meta-analyses were performed using Review Manager 5.4, and certainty of evidence was assessed using the GRADEpro online tool. Results: A total of 10 studies involving 3977 pregnant women were included. Combined diet and physical activity interventions significantly reduced total gestational weight gain (GWG) (MD = −0.78 kg, 95% CI: −1.12 to −0.44, *p* < 0.00001) and the risk of excessive gestational weight gain (EGWG) (OR = 0.63, 95% CI: 0.49–0.81, *p* = 0.0003). Additionally, individually delivered physical activity components and those implemented in healthcare facilities appeared to be associated with lower total GWG (MD = −0.76 kg, 95% CI: −0.98 to −0.53, *p* < 0.00001). For EGWG, lower risk was observed in interventions using combined face-to-face and remote formats (OR = 0.54, 95% CI: 0.41–0.72, *p* < 0.0001) and moderate frequency (diet: OR = 0.64, 95% CI: 0.51–0.81, *p* = 0.0002, physical activity: OR = 0.65, 95% CI: 0.52–0.83, *p* = 0.0004). Conclusions: Combined diet and physical activity interventions were associated with reduced total GWG and lower EGWG risk in low-risk pregnant women. Intervention characteristics, such as individual delivery formats, combined face-to-face and remote formats, moderate frequency and implementation in healthcare facilities, may be related to intervention effectiveness. Registration number: CRD420251013116 (PROSPERO).

## 1. Introduction

Gestational weight gain (GWG) is a critical indicator of maternal nutritional status during pregnancy and plays a pivotal role in determining both maternal and offspring health outcomes [1]. Inadequate or excessive gestational weight gain may lead to adverse consequences. Among these, excessive gestational weight gain (EGWG) has emerged as a significant global public health concern, with prevalence estimates ranging from 37% to 51% across various populations [2]. EGWG is typically defined as gestational weight gain exceeding the upper limits of recommended ranges [3,4]. Robust evidence has demonstrated that EGWG is closely associated with a heightened risk of adverse maternal outcomes, including gestational diabetes mellitus, hypertensive disorders of pregnancy, and cesarean section [1,5]. Importantly, EGWG has also been linked to long-term maternal morbidities, such as postpartum weight retention [6], persistent low back and pelvic pain [7], and increased cardiovascular risk [8]. In addition to maternal consequences, EGWG is also associated with a higher incidence of adverse offspring outcomes, including macrosomia [4], childhood obesity [9] and allergic diseases [10]. Accumulating evidence suggests that structured antenatal interventions incorporating both diet and physical activity can effectively optimize gestational weight gain, reduce the risk of EGWG, improve perinatal outcomes [11,12], and reduce the burden on healthcare systems [13,14,15]. Accordingly, combined diet and physical activity interventions are considered a promising strategy to optimize gestational weight management [16].

Notably, most existing studies on combined diet and physical activity interventions have predominantly focused on overweight/obese women [17,18,19] or on heterogeneous populations across different BMI categories [11,16], with relatively limited evidence specifically targeting low-risk pregnant women. However, despite being generally classified as low-risk, women with normal pre-pregnancy BMI and no pregnancy complications still bear a substantial burden of EGWG. Santos et al. [20] reported that 33.1% of women with normal pre-pregnancy BMI exceeded recommended gestational weight gain ranges in an individual participant data analysis of 218,216 pregnant women. Similarly, a meta-analysis including populations from the United States, Europe, and East Asia found that approximately 36% of women with normal pre-pregnancy BMI experienced EGWG [2]. Importantly, EGWG in this population is also associated with adverse outcomes, such as gestational diabetes [21], preterm birth [22] and offspring neurodevelopmental disorders [23]. Furthermore, low-risk pregnancies constitute the majority of the obstetric population [24]. Given their numerical predominance, addressing EGWG in this group may have substantial public health implications. A systematic review by Cantor et al. [12] reported greater intervention effects among overweight and obese pregnant women, suggesting that responsiveness to lifestyle interventions may vary by pre-pregnancy BMI classification. In addition, pre-pregnancy BMI influences maternal metabolic profiles and pregnancy outcomes [25,26], further supporting the need to evaluate gestational weight management interventions across different risk groups. Thus, evidence derived from mixed or high-risk populations may not be directly generalizable to low-risk women due to differences in baseline risk and intervention responsiveness. Overall, the effectiveness of combined diet and physical activity interventions among low-risk pregnant women remains insufficiently characterized and warrants further investigation.

A systematic review by O’Brien et al. [27] in 2015, focusing on low-risk women, indicated that combined diet and physical activity interventions could reduce both total GWG and the incidence of EGWG. However, for each of these two outcomes, the pooled sample size was fewer than 1000 participants, and fewer than five studies contributed data, limiting the ability to conduct subgroup or sensitivity analyses and reducing the generalizability of the findings. Furthermore, recent trials have reported inconsistent results in this population: studies by Sagedal [28], Krebs [29] and Yang [30] showed that such interventions were effective in reducing total GWG, whereas those by Kunath [31] and Dodd [32] did not observe statistically significant effects. These inconsistencies highlight the need for an updated and methodologically robust systematic review and meta-analysis to comprehensively evaluate the impact of combined interventions on gestational weight gain among low-risk pregnant women.

Although combined diet and physical activity interventions are recommended for managing gestational weight gain [16,33], the prevalence of EGWG remains high [2,34]. One potential explanation is the gap between intervention efficacy demonstrated in controlled research settings and the diet and physical activity implementation in routine clinical practice [35,36]. Identifying effective components of interventions and their feasible implementation pathways is crucial for advancing the translation into clinical practice [33]. However, most existing systematic reviews focus primarily on overall effectiveness, with limited attention to key intervention characteristics, such as the timing of initiation, duration, intensity and implementers [37], thereby constraining the practical applicability of evidence. This issue is particularly relevant for low-risk pregnant women, among whom the implementation characteristics of combined diet and physical activity interventions have not been systematically described. The Template for Intervention Description and Replication (TIDieR) checklist [38], a standardized tool for reporting and replicating interventions, provides a structured framework for describing key components, such as frequency, mode of delivery and provider. Lim et al. [39] applied the TIDieR checklist in a systematic review and meta-analysis of 33 randomized controlled trials, identifying healthcare professional support and the integration of dietary and physical activity components as important features of effective postpartum weight management interventions. Accordingly, the TIDieR checklist offers a useful framework for identifying intervention components and exploring their associations with gestational weight gain, thereby informing the development of targeted strategies for low-risk pregnant women.

This study aims to assess the effects of combined diet and physical activity interventions on gestational weight gain in low-risk pregnant women. Intervention components and implementation features will be systematically described using the TIDieR checklist to identify potentially effective elements. The findings are expected to inform the standardization and optimization of gestational weight management strategies and provide preliminary evidence to support clinical decision-making.

## 2. Methods

This systematic review was conducted in accordance with the *Cochrane Handbook for Systematic Reviews of Interventions* [40] and reported following the Preferred Reporting Items for Systematic Reviews and Meta-Analyses (PRISMA) guidelines [41] (Supplementary Table S1). The study protocol was prospectively registered in PROSPERO (CRD420251013116).

### 2.1. Literature Search Strategy

A comprehensive search strategy was implemented to identify both published and unpublished studies. Peer-reviewed articles were retrieved from four databases: PubMed, Embase, CINAHL Plus and Web of Science. Completed but unpublished trials were identified from three clinical trial registries: the WHO International Clinical Trials Registry Platform, ClinicalTrials.gov and the Cochrane Central Register of Controlled Trials. Gray literature was accessed via the ProQuest Dissertations & Theses database. The search spanned from database inception to 16 March 2026. Additionally, the reference lists of included studies were manually screened to identify potentially relevant studies not captured in the primary search.

The search strategy was developed based on the PICO framework with guidance from a medical information specialist. It included Medical Subject Headings (MeSH) and free-text terms, combined using Boolean operators (AND/OR). Search terms included, but were not limited to, “pregnant women,” “diet,” “physical activity” and “randomized controlled trial.” The full search strategy is presented in Supplementary File S1.

### 2.2. Inclusion and Exclusion Criteria

#### 2.2.1. Inclusion Criteria

Studies were eligible for inclusion if they met the following criteria:

(1) Population: 18 years or above; single fetus; normal pre-pregnancy BMI (defined as 18.5–24.9 kg/m^2^); without pregnancy complications (e.g., gestational diabetes, gestational hypertension). Studies including mixed-BMI populations were included only if subgroup-specific data for women with normal pre-pregnancy BMI were reported in the original studies and could be extracted for analysis.

(2) Intervention: Combined diet and physical activity interventions implemented during pregnancy, with both components required for inclusion.

(3) Comparator: Standard prenatal care, usual prenatal care or routine prenatal care.

(4) Outcomes: Studies had to report total gestational weight gain, either as a continuous value or the incidence of EGWG (defined as total GWG exceeding the recommended ranges).

(5) Study Design: Cluster randomized controlled trial (CRCT), randomized controlled trial (RCT), follow-up of RCT, or secondary analyses of RCT.

#### 2.2.2. Exclusion Criteria

Studies were excluded if they (1) lacked full-text availability, (2) were not peer-reviewed (e.g., conference abstracts, protocols), (3) were duplicate publications, (4) did not report GWG outcomes or presented incomplete data. For duplicate publications, the most complete version was retained.

### 2.3. Study Selection and Data Extraction

Two reviewers (WJL and HSW) independently performed the study selection in three stages. First, all records were imported into EndNote 20 to remove duplicates. Second, titles and abstracts were screened using predefined eligibility criteria and classified as “included”, “excluded” or “uncertain”. Third, full texts of potentially eligible and “uncertain” records were reviewed to determine final inclusion. Disagreements were resolved through discussion or adjudicated by a third reviewer.

**Figure 1:**
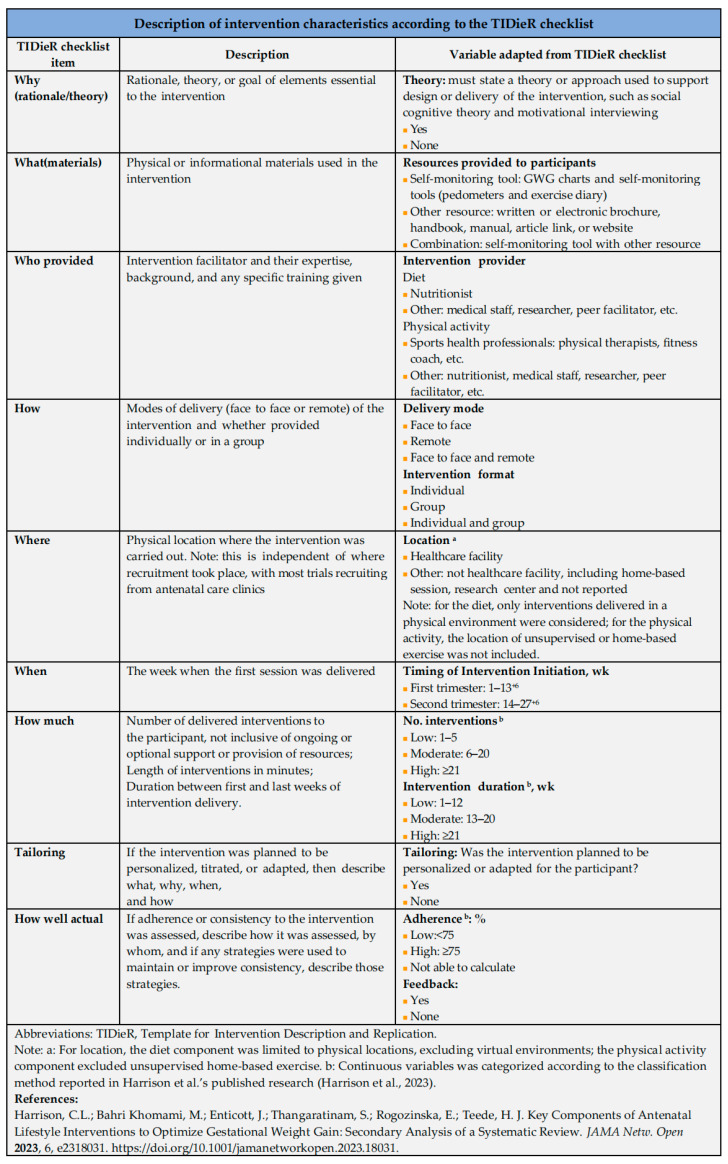
Description of intervention characteristics according to the TIDieR checklist [42].

Data extraction was also conducted independently by the two reviewers (WJL and HSW) using a standardized data extraction form. Disagreements were also resolved through discussion or adjudicated by a third reviewer. Extracted data included study characteristics and detailed intervention features. The study characteristics included the first author’s name, publication year, country, study design, sample size, descriptions of intervention and control conditions, and outcomes relevant to total gestational weight gain. Intervention characteristics were primarily coded using the TIDieR checklist, with partial reference to Harrison et al.’s [42] classification scheme. Key elements extracted included initiation timing, theoretical rationale, resources, feedback, providers, delivery mode and format, frequency, duration, location, tailoring and adherence. A summary of intervention characteristics is provided in Figure 1.

### 2.4. Risk of Bias Assessment

Two reviewers (WJL and HSW) independently assessed the risk of bias using the Cochrane Risk of Bias 2.0 tool (https://www.riskofbias.info/, accessed on 29 July 2025). This tool evaluates five domains: randomization process, deviations from intended interventions, missing outcome data, measurement of outcomes, and selection of the reported results. Each domain was rated as “low risk,” “some concerns,” or “high risk.” Any disagreements were resolved by discussion or arbitration by a third reviewer.

### 2.5. Data Analyses and Certainty Assessment of the Body of Evidence

Meta-analyses were conducted using Review Manager 5.4. For dichotomous outcomes (e.g., incidence of EGWG), odds ratios (ORs) with 95% confidence intervals (CIs) were calculated. For continuous outcomes (e.g., total GWG), mean differences (MDs) with 95% CIs were used. A two-tailed *p*-value < 0.05 was considered statistically significant. When cluster-randomized controlled trials were included, the design effect method recommended in the *Cochrane Handbook for Systematic Reviews of Interventions* [40] was applied to calculate effective sample sizes to account for clustering. For dichotomous outcomes, both the number of participants and events were divided by the same design effect. For continuous outcomes, only the sample size was adjusted, while means and standard deviations remained unchanged. Adjusted effective sample sizes were used in all meta-analyses to ensure comparability between cluster-randomized and individually randomized trials. The design effect was calculated as:

$$design\;effect=1+\left(M-1\right)\times ICC$$

Heterogeneity among studies was assessed using the I^2^ statistic and Cochran’s Q test. If the *p* ≤ 0.1 and *I*^2^ > 50%, indicating substantial heterogeneity, potential sources were explored through subgroup analysis or sensitivity analysis. Moreover, heterogeneity test determined whether to use the fixed-effect model (*p* > 0.1, *I*^2^ ≤ 50%) or the random-effect model (*p* ≤ 0.1, *I*^2^ > 50%). The random-effect model was also adopted when both RCTs and CRCTs were included to account for methodological heterogeneity. Sensitivity analyses were performed by excluding one study at a time to assess the robustness of pooled estimates. Subgroup analyses were conducted to examine the effects of specific characteristics of combined diet and physical activity interventions on gestational weight outcomes, including total GWG and the incidence of EGWG, in low-risk pregnant women. Intervention characteristics were identified and categorized based on the TIDieR checklist through a structured extraction process and were used as predefined variables for subgroup analyses. The classification framework is presented in Figure 1. Publication bias was assessed using funnel plots if ≥10 studies were included.

The certainty of evidence for each outcome was independently evaluated by two reviewers (WJL and HSW) using the GRADEpro Guideline Development Tool (https://gdt.gradepro.org/, accessed on 29 July 2025). Each outcome was initially rated as “high certainty” due to the inclusion of RCT, and could be downgraded based on five domains: risk of bias, inconsistency, indirectness, imprecision and publication bias. Final ratings were categorized as “high,” “moderate,” “low,” or “very low.” Discrepancies were resolved by consensus or third-party adjudication.

## 3. Results

### 3.1. Search Results and Study Selection

**Figure 2:**
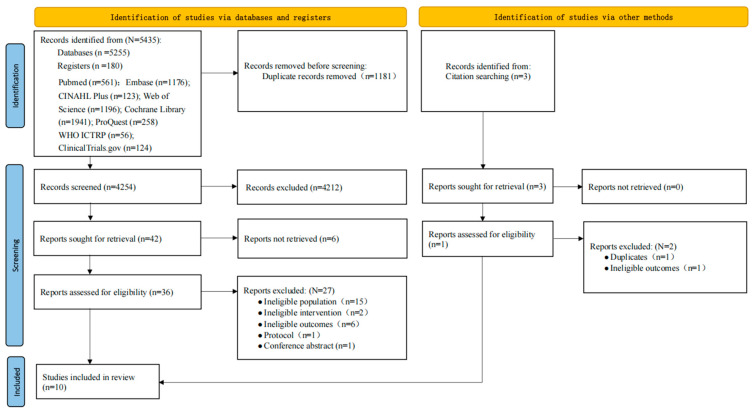
The PRISMA flowchart of search results and study selection.

A total of 5438 records were identified through database searches (*n* = 5255) and trial registries (*n* = 180), with an additional three studies retrieved through citation searching. After removing 1181 duplicates using EndNote 20, 4254 records remained. Following title and abstract screening, 4212 records were excluded due to irrelevance or failure to meet eligibility criteria. Full-text articles were retrieved for 42 potentially eligible studies, but 6 articles were not available in full text, resulting in 36 studies assessed for full-text eligibility. Based on predefined inclusion and exclusion criteria, 27 studies were excluded. In addition, one study was supplemented through manual retrieval of the reference lists from the included studies. The reasons for exclusion are detailed in Supplementary File S2. Ultimately, 10 studies were included in this systematic review (see Supplementary File S3). The study selection process is illustrated in the PRISMA flowchart (Figure 2).

### 3.2. Study Characteristics

A total of ten studies [28,29,30,31,32,43,44,45,46,47] were included, comprising eight individually randomized controlled trials and two cluster-randomized trials [29,31]. These studies were conducted across six countries: the United States (*n* = 3, 33.3%), Canada (*n* = 2, 20%), Germany (*n* = 2, 20%), and one study each from Norway, Australia and China (each *n* = 1, 10%). Overall, the included studies enrolled 3977 pregnant women, with 1990 assigned to intervention groups and 1987 to control groups. And these studies were published between 2002 and 2023. To ensure methodological consistency, effective sample sizes for the two included cluster-randomized trials [29,31] were calculated using the design effect approach, and the adjusted sample sizes were used in all meta-analyses. Detailed calculations are provided in Supplementary File S4.

Two studies [30,32] exclusively recruited women with normal pre-pregnancy BMI, while the remaining eight included broader BMI populations but reported stratified results for the normal-BMI subgroup. Control interventions included standard antenatal care, routine prenatal care or usual care. Regarding outcomes, all studies reported total GWG in kilograms. Eight studies reported the incidence of EGWG, defined as total GWG exceeding the Institute of Medicine (IOM) recommended range [3]. Four studies reported the proportion of women with total GWG within the IOM-recommended range and below the IOM-recommended range. Detailed characteristics are presented in Table 1.

Intervention characteristics were extracted and coded using the TIDieR checklist. Among the ten intervention arms, two interventions commenced in the first trimester, while the remaining eight arms began in the second trimester. Two studies explicitly reported use of a theoretical framework to guide intervention design. Most interventions incorporated feedback (7/10). Moreover, an individual format was predominant. Nutritionists were the providers for diet interventions in five groups, while physical activity interventions were delivered by sports health professionals in one group. Most interventions were conducted in a healthcare facility (8/10). In terms of intensity, most interventions lasted ≥21 weeks (diet: 6/10, physical activity: 7/10). Diet components were typically of moderate frequency (6–20 sessions in 6 arms), while physical activity components were predominantly high frequency (≥21 sessions in 6 arms). Tailoring was common in diet components (10/10) but less frequent in physical activity components (6/10). Regarding additional resources, both components frequently relied on “Other resource” (diet: 7/10, physical activity: 6/10). Detailed intervention characteristics are summarized in Table 2. Among the studies with available adherence data (7/10), the adherence levels were all ≥ 75%, so no subgroup analysis was conducted on this characteristic.

### 3.3. Risk of Bias Assessment

**Figure 3:**
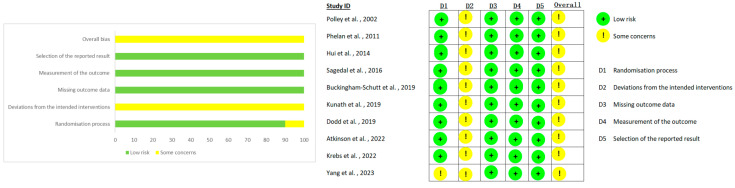
Summary of risk of bias for included studies [28,29,30,31,32,43,44,45,46,47].

Risk of bias was assessed using the Cochrane Risk of Bias 2.0 tool (Figure 3). Overall, all 10 studies were rated as “some concerns” in the domain of deviations from intended interventions, as blinding is generally not feasible in combined diet and physical activity intervention trials. Additionally, one study [30] presented additional risks regarding the randomization process. A detailed risk of bias summary is provided in Supplementary File S5.

### 3.4. Effects of Combined Diet and Physical Activity Interventions on Gestational Weight Gain Outcomes

**Figure 4:**
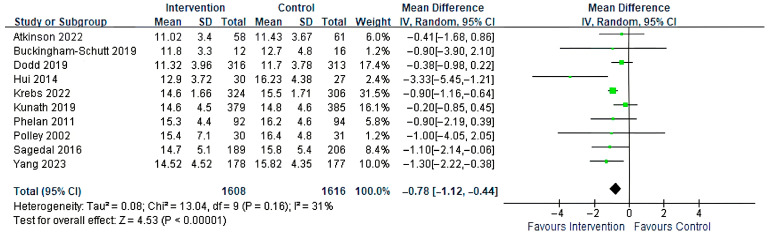
Forest plot of the effect of diet with physical activity on total GWG [28,29,30,31,32,43,44,45,46,47].

Ten studies assessed the effect of combined diet and physical activity interventions on total GWG. Meta-analysis using a random-effects model showed a significant reduction compared with the control group (MD= −0.78 kg, 95% CI: −1.12 to −0.44, *p* < 0.00001; Figure 4).

**Figure 5:**
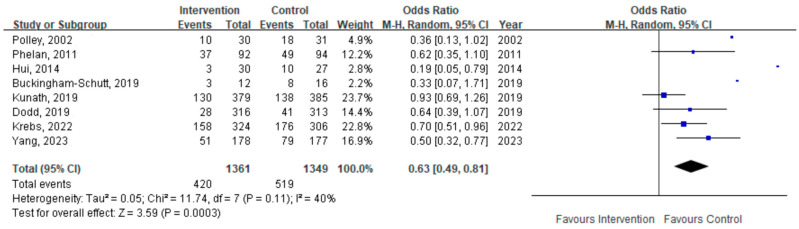
Forest plot of the effect of diet with physical activity on EGWG [29,30,31,32,43,44,45,46].

Eight studies reported the incidence of EGWG. Meta-analysis using a random-effects model indicated a significantly reduction in the risk of EGWG (OR = 0.63, 95% CI: 0.49–0.81, *p* = 0.0003; Figure 5).

**Figure 6:**
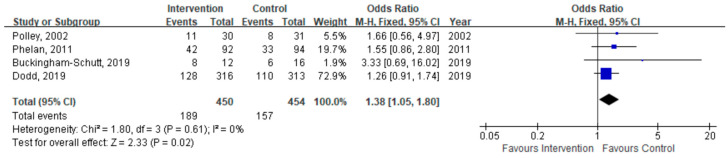
Forest plot of the effect of diet with physical activity on the proportion of women with total GWG within the IOM-recommended range [32,43,44,46].

Four studies reported the proportion of women with total GWG within the IOM-recommended range. Fixed-effects meta-analysis showed that combined interventions were associated with significantly increased odds of achieving IOM-recommended weight gain compared to controls (OR = 1.38, 95% CI: 1.05–1.80, *p* = 0.02; Figure 6).

Four studies assessed the proportion of women with total GWG below the IOM-recommended range. However, the pooled analysis did not show a statistically significant association between the combined intervention and risk of insufficient GWG (*p* = 0.92; Supplementary File S6, Figure S1).

### 3.5. Subgroup Analyses of Combined Interventions on Total GWG Based on TIDieR Checklist

All included studies evaluated the effects of combined diet and physical activity interventions on total GWG, and subgroup analysis results are summarized in Table 3. In subgroup analyses, greater reductions in total GWG were observed in studies with individually delivered physical activity interventions (MD = −0.76 kg, 95% CI: −0.98 to −0.53, *p* < 0.00001) and those conducted in healthcare settings (MD = −0.76 kg, 95% CI: −0.98 to −0.53, *p* < 0.00001). Additionally, reductions in total GWG were also observed in studies without a reported theoretical framework (MD = −0.88 kg, 95% CI: −1.30 to −0.45, *p* < 0.0001). Interventions utilizing “Other resources” showed a statistically significant benefit (diet: MD = −0.90 kg, 95% CI: −1.30 to −0.50, *p* < 0.0001, physical activity: MD = −0.96 kg, 95% CI: −1.41 to −0.51, *p* < 0.0001). No significant differences were observed across subgroups based on timing of intervention initiation, feedback, delivery mode, provider, duration, session frequency or tailoring. The detailed forest plot of the subgroup analysis can be found in Supplementary File S6.

### 3.6. Subgroup Analyses of Combined Interventions on EGWG Based on TIDieR Checklist

Among the eight studies evaluating the effects of combined interventions on EGWG, six studies (75.0%) were initiated during the second trimester and were not informed by a theoretical framework. Additionally, five studies (62.5%) were more frequently delivered using a combination of face-to-face and remote modalities, and seven studies (87.5%) incorporated feedback. Most interventions were delivered individually, accounting for 100% of the diet and 87.5% of the physical activity interventions. Nutritionists provided the diet component in 50.0% of cases, while sports health professionals led the physical activity component in 12.5%. The majority of interventions were delivered in healthcare facilities (87.5%). Moderate frequency (6–20 sessions) predominated in the diet component (62.5%), and was reported in 50.0% of the physical activity interventions. Most interventions were of long duration (≥21 weeks) (diet: 62.5%, physical activity: 75.0%). Tailoring was incorporated into all diets and 62.5% of the physical activity components. More than half of both components utilized “Other resource” (62.5%). More detailed results are presented in Table 4.

In subgroup analyses, a lower risk of EGWG was observed in studies using combined face-to-face and remote delivery (OR = 0.54, 95% CI: 0.41–0.72, *p* < 0.0001). Moderate frequency (6–20 sessions) was significantly associated with lower EGWG in both diet (OR = 0.64, 95% CI: 0.51–0.81, *p* = 0.0002) and physical activity components (OR = 0.65, 95% CI: 0.52–0.83, *p* = 0.0004). The use of “Other resource” in both components was also associated with reduced EGWG (OR = 0.57, 95% CI: 0.44–0.76, *p* < 0.0001). No significant associations were observed for other subgroup analyses, including timing of intervention initiation, theoretical framework, feedback, intervention format, provider, location, duration and tailoring. The more detailed forest plot of the subgroup analysis is presented in Supplementary File S6.

### 3.7. Sensitivity Analyses and Certainty of the Body of Evidence

Heterogeneity in the results regarding the effects of combined diet and physical activity interventions on gestational weight gain outcomes was not substantial. The results for total GWG, the incidence of EGWG and the proportion of women with total GWG below the IOM-recommended range remained unchanged through sensitivity analyses, indicating good stability of these outcomes. However, after excluding the study by Phelan et al. [43], the pooled effect estimate for the proportion of women with total GWG within the IOM-recommended range changed from statistically significant (OR = 1.38, 95% CI: 1.05–1.80, *p* = 0.02) to non-significant (OR = 1.33, 95% CI: 0.98–1.81, *p* = 0.06). This suggests that the result for this outcome was sensitive to the inclusion of a single study and should therefore be interpreted with caution. Detailed results are presented in Supplementary File S7. Furthermore, potential publication bias was assessed via funnel plot for the outcome of total GWG (n = 10). The plot showed a generally symmetrical distribution, indicating a low risk of publication bias (see Supplementary File S6, Figure S37).

The certainty of evidence was evaluated using the GRADE guideline. The certainty of evidence was rated as “moderate” for total GWG, the incidence of EGWG, and the proportion of women with total GWG within the IOM-recommended range, primarily due to concerns related to risk of bias. In contrast, the certainty of evidence for the proportion of women with total GWG below the IOM-recommended range was judged to be “low”, owing to both risk of bias and imprecision. The full GRADE assessment process is presented in Supplementary File S8.

## 4. Discussion

This systematic review and meta-analyses provide evidence that combined diet and physical activity interventions are associated with reductions in both total GWG and the risk of EGWG among low-risk pregnant women. Subgroup analyses further suggested that an individual format, a combined face-to-face and remote mode, moderate frequency (6–20 sessions) and implementation in healthcare facilities may be associated with more favorable gestational weight management outcomes.

### 4.1. Interpretation of Findings

Our findings indicate that, among women with normal pre-pregnancy BMI and no pregnancy complications, combined diet and physical activity interventions were associated with reduced total GWG and a lower risk of EGWG. These findings are broadly consistent with previous meta-analyses, while providing additional context specific to low-risk populations. A review focusing on women with normal BMI by O’Brien et al. [27] reported a significant reduction in both GWG (MD = −1.25 kg, 95% CI: −2.39 to −0.11) and EGWG risk (OR = 0.66, 95% CI: 0.53–0.83) following combined diet and physical activity interventions. Similarly, Teede et al. [16], in a comprehensive meta-analysis including 34,546 participants across 117 randomized controlled trials without BMI stratification, reported reductions in GWG associated with combined diet and physical activity interventions (MD = −1.35 kg, 95% CI: −1.95 to −0.75). Furthermore, Grieger et al. [48] conducted a meta-analysis of 53 randomized controlled trials involving 17,596 pregnant women, which also showed that combined diet and physical activity interventions significantly reduced total GWG (MD = −0.82 kg, 95% CI: −1.45 to −0.18). Taken together, these findings suggest a consistent direction of effects of combined diet and physical activity interventions across populations with different risk profiles. However, the magnitude and potential clinical implications of these effects may vary according to population characteristics, and direct extrapolation between high-risk and low-risk pregnant women should be approached with caution. In addition, this review did not evaluate maternal or neonatal clinical outcomes, such as mode of delivery or infant birthweight. Future research should incorporate both short- and long-term health outcomes to support more comprehensive clinical decision-making.

Subgroup analyses further indicated that individually delivered physical activity interventions may be associated with reduced GWG. This finding is consistent with the perspective proposed by Raab et al. [49], who emphasized that women’s needs and barriers regarding weight management during pregnancy vary substantially. Individualized interventions may enhance the alignment between intervention content and individual needs based on physical condition, lifestyle patterns and personal differences [50], thereby improving the acceptability and adherence of the intervention. Regarding intervention formats, interventions incorporating both face-to-face and remote components appeared to be associated with a lower risk of EGWG. This pattern may reflect the complementary strengths of different delivery modalities. On one hand, face-to-face interventions may facilitate the establishment of trust, enhance the effectiveness of professional guidance, and meet pregnant women’s need for direct communication [51]. On the other hand, remote intervention formats may offer greater flexibility in time and location, thereby reducing participation barriers to some extent and promoting sustained implementation of interventions [52,53]. The combination of these elements may therefore represent a potentially useful approach, although further research is required to confirm this pattern.

Notably, this review also suggested that interventions conducted in healthcare facilities were more likely to be associated with reductions in total GWG, and moderate frequency may be associated with lower EGWG risk. Most included studies conducted interventions within routine antenatal visits, which may explain these findings, as such integration simultaneously defines the healthcare setting and generates moderate-frequency contact. Integrating weight management strategies into existing healthcare infrastructures may improve accessibility, strengthen patient–provider relationships, and promote sustained behavioral reinforcement [54], thereby enhancing overall intervention effectiveness. Therefore, embedding combined diet and physical activity interventions into routine prenatal care may be a promising direction. However, the extent to which these features independently contribute to effectiveness remains to be further examined in more rigorously designed studies.

With respect to supportive components, the provision of “Other resources” alone appeared to be associated with reductions in both total GWG and EGWG risk, whereas combinations including self-monitoring tools did not show similar benefits. This finding may suggest potential differences in underlying mechanisms across resource types. One possible explanation is that self-monitoring requires sustained active engagement from participants, such as regular recording of weight, diet and physical activity, which may increase behavioral burden in certain contexts [55]. Accordingly, when designing future interventions, the selection and combination of supportive resources may need to consider the characteristics of both the target population and the implementation context.

Additionally, subgroup analyses based on theoretical frameworks indicated that interventions not explicitly reported as theory-based were associated with reducing total GWG. This finding warrants cautious interpretation. Firstly, the small number of included studies in the theoretical framework subgroup (*n* = 2) and limited sample sizes may compromise the stability of the effect estimate. Furthermore, some interventions may have incorporated theory-informed behavior change techniques without explicitly reporting the underlying theoretical framework, potentially leading to misclassification bias. More importantly, the effectiveness of theory-driven interventions does not depend on the formal labeling of theoretical names but rather on whether theoretical concepts are clearly translated into specific intervention content and implemented with high quality [56]. Therefore, this finding should not be interpreted as evidence against the value of theoretical frameworks in the design of gestational weight management. Conversely, it suggests that when assessing the impact of theoretical frameworks on intervention effectiveness, it is necessary to further focus on the reporting quality and implementation depth of theoretical application. Future research should more systematically describe theoretical foundations and specify how core constructs are translated into practice to enable more rigorous evaluation of theory-informed intervention design.

### 4.2. Strengths and Limitations

This review contributes to the current evidence by focusing specifically on low-risk pregnant women, a population that has been relatively underrepresented, and by systematically evaluating the effects of combined diet and physical activity interventions on gestational weight gain. Through the application of the TIDieR checklist and subsequent subgroup analyses, this study provides a structured characterization of intervention components and identifies intervention features that may be associated with variations in effectiveness. These findings may offer context-specific insights to inform the design and implementation of gestational weight management strategies within routine clinical practice.

Nevertheless, several limitations should be acknowledged. Firstly, reporting completeness according to the TIDieR checklist was suboptimal across included studies, requiring interpretive judgment during data extraction, which may have introduced classification bias. Secondly, although this review targeted women with normal pre-pregnancy BMI, some evidence was derived from subgroup data within studies including broader BMI populations, which may limit the strength of inferences specific to low-risk women. Moreover, the statistical power of certain subgroup analyses, such as those evaluating “theory-based” interventions, may be limited due to the small number of eligible studies. Finally, this review focused exclusively on gestational weight-related outcomes and did not assess maternal or neonatal outcomes. Future research should expand the scope of outcome evaluation to include maternal, neonatal, long-term health and economic indicators to better inform policy and clinical practice for low-risk populations.

## 5. Conclusions

This systematic review and meta-analysis found that combined diet and physical activity interventions are associated with reductions in total GWG and the incidence of EGWG among low-risk pregnant women. Subgroup analyses based on the TIDieR checklist showed several intervention characteristics that may be relevant to intervention effectiveness in managing gestational weight gain, including individual delivery formats, a combination of face-to-face and remote mode, moderate frequency and implementation in healthcare facilities. These findings provide preliminary evidence that such interventions may be beneficial in low-risk pregnant women and offer insights to inform future research and intervention development. Further well-designed studies are needed to confirm these observations and to clarify the role of specific intervention components in gestational weight management.

## Figures and Tables

**Table 1:** Characteristics of the included studies.

First Author	Published Year	Country	Study Type	Sample Size (I/C)	Pre-Pregnancy BMI (kg/m^2^)	Parity	Gestational Age at Recruitment (wk)	Intervention	Control	Outcomes ^a^
Polley	2002	the United States	RCT	61 (30/31)	NA	Primiparous, Multiparous	≤20	Diet with physical activity	Standard Prenatal Care	I, II, III, IV
Phelan	2011	the United States	RCT	186 (92/94)	19.8–40	Primiparous, Multiparous	10–16	Diet with physical activity	Standard Prenatal Care	I, II, III, IV
Hui	2014	Canada	RCT	57 (30/27)	NA	NA	≤20	Diet with physical activity	Standard Prenatal Care	I, II
Sagedal	2016	Norway	RCT	395 (189/206)	≥19	Primiparous	≤20	Diet with physical activity	Routine Prenatal Care	I
Buckingham–Schutt	2019	the United States	RCT	28 (12/14)	18.5–45.5	Primiparous, Multiparous	8–14	Diet with physical activity	Usual Prenatal Care	I, II, III, IV
Kunath	2019	Germany	CRCT	1232 (608/624)	18.5–40	Primiparous, Multiparous	<14	Diet with physical activity	Routine Prenatal Care	I, II
Dodd	2019	Australia	RCT	629 (316/313)	18.5–24.9	Primiparous, Multiparous	10–20	Diet with physical activity	Standard Prenatal Care	I, II, III, IV
Atkinson	2022	Canada	RCT	119 (58/61)	<40	Primiparous, Multiparous	12–17	Diet with physical activity	Usual Care	I
Krebs	2022	Germany	CRCT	915 (477/438)	NA	Primiparous, Multiparous	<12	Diet with physical activity	Usual Care	I, II
Yang	2023	China	RCT	355 (178/177)	18.5–24	Primiparous	8–12	Diet with physical activity	Routine Prenatal Care	I, II

Note: NA: not available. a: Outcomes: I: total gestational weight gain (kg); II: the incidence of excessive gestational weight gain; III: the proportion of women with total GWG within the IOM-recommended range; IV: the proportion of women with total GWG below the IOM-recommended range [28,29,30,31,32,43,44,45,46,47].

**Table 2:** Characteristics of intervention according to the TIDieR checklist.

Study	Intervention										
				Diet				Physical Activity			
	Timing of Intervention Initiation	Theory; Feedback	Adherence	Delivery Mode ^a^; Intervention Format ^b^	Provider ^c^; Location ^d^	Duration, wk; No. Interventions	Tailoring; Resources ^e^	Delivery Mode ^a^; Intervention Format ^b^	Provider ^c^; Location ^d^	Duration, wk; No. Interventions	Tailoring; Resources ^e^
Polley 2002	Second trimester	None; Yes	93.75%	III; I	Other; Healthcare facility	18; 8	Yes; II	III; I	Other; Healthcare facility	18; 8	Yes; II
Phelan 2011	Second trimester	Yes; Yes	NA	III; I	Nutritionist; Healthcare facility	22; 14	Yes; III	III; I	Other; Healthcare facility	22; 14	None; III
Hui 2014	Second trimester	None; Yes	100%	I; I	Nutritionist; NA	8; 2	Yes; II	I; III	Fitness Coach; Fitness center	28; 54	None; II
Sagedal 2016	Second trimester	None; None	94.03%	II; III	Other; NA	18; 4	Yes; II	I; III	Other; Fitness center	18; 36	None; II
Buckingham-Schutt 2019	Second trimester	None; Yes	100%	III; I	Nutritionist; Healthcare facility	24; 6	Yes; III	III; I	Other; Healthcare facility	24; 174	None; III
Kunath 2019	Second trimester	None; Yes	NA	I; I	Other; Healthcare facility	22; 3	Yes; III	I; I	Other; Healthcare facility	22; 25	Yes; III
Dodd 2019	Second trimester	Yes; Yes	99.06%	III; I	Nutritionist; Healthcare facility	16; 6	Yes; II	III; I	Nutritionist; Healthcare facility	16; 6	Yes; II
Atkinson 2022	Second trimester	None; None	95.08%	I; I	Nutritionist; Healthcare facility	21; 11	Yes; II	I; I	Nutritionist; Healthcare facility	21; 63	Yes; I
Krebs 2022	First trimester	None; None	NA	I; I	Other; Healthcare facility	25; 6	Yes; II	I; I	Other; Healthcare facility	25; 6	Yes; II
Yang 2023	First trimester	None; Yes	100%	III; I	Other; Healthcare facility	27; 5	Yes; II	III; I	Other; Healthcare facility	27; 32	Yes; II

Note: NA: not available. a: I: Face to face; II: Remote; III: Face to face and remote; b: I: Individual; II: Group; III: Individual and group; c: Other: Doctors, nurses, midwives, researchers, etc., other than nutritionist and sports health professionals (fitness coach, physical therapists, etc.); d: Other: not healthcare facility, including home-based session, research center and not reported; e: I: Self-monitoring tool; II: Other resource: written or electronic brochure, handbook, manual, article link, or website; III: Combination: I and II [28,29,30,31,32,43,44,45,46,47].

**Table 3:** Subgroup analyses of combined interventions on total GWG based on TIDieR checklist.

TIDieR Intervention Characteristics	Intervention Groups (No.)	Participants	GWG, MD (95% CI), kg ^a^	*I*^2^ (%)	*p*-Value for Subgroup Differences
**Timing of Intervention Initiation**					0.33
First trimester	2	985	−0.93 [−1.18, −0.68]	0	
Second trimester	8	2239	−0.66 [−1.14, −0.19]	27	
**Theory based**					0.25
Yes	2	815	−0.47 [−1.02, 0.07]	0	
None	8	2409	−0.88 [−1.30, −0.45]	36	
**Feedback**					0.81
Yes	7	2080	−0.81 [−1.40, −0.22]	45	
None	3	1144	−0.89 [−1.14, −0.64]	0	
**Delivery Mode**					
Diet					0.78
Face to face	4	1570	−0.81 [−1.53, −0.09]	69	
Remote	1	395	−1.10 [−2.14, −0.06]	-	
Face-to-face and remote	5	1259	−0.70 [−1.16, −0.24]	0	
Physical activity					0.72
Face to face	5	1965	−0.83 [−1.41, −0.26]	60	
Remote	0				
Face-to-face and remote	5	1259	−0.70 [−1.16, −0.24]	0	
**Intervention format**					
Diet					0.54
Individual	9	2829	−0.76 [−1.14, −0.38]	37	
Group	0				
Individual and Group	1	395	−1.10 [−2.14, −0.06]	-	
Physical activity					0.25
Individual	8	2772	−0.76 [−0.98, −0.53]	2	
Group	0				
Individual and Group	2	452	−2.01 [−4.16, 0.14]	71	
**Provider**					
Diet					0.92
Nutritionist	5	1019	−0.87 [−1.68, −0.05]	44	
Other	5	2205	−0.82 [−1.17, −0.47]	23	
Physical activity					0.02
Sports health professionals	1	57	−3.33 [−5.45, −1.21]	-	
Other	9	3167	−0.78 [−0.99, −0.57]	0	
**Location**					
Diet					0.25
Healthcare facility	8	2772	−0.76 [−0.98, −0.53]	2	
Other	2	452	−2.01 [−4.16, 0.14]	71	
Physical activity					0.25
Healthcare facility	8	2772	−0.76 [−0.98, −0.53]	2	
Other	2	452	−2.01 [−4.16, 0.14]	71	
**Duration, wk**					
Diet					0.05
Low: 1–12	1	57	−3.33 [−5.45, −1.21]	-	
Moderate: 13–20	3	1085	−0.58 [−1.09, −0.06]	0	
High: ≥21	6	2082	−0.80 [−1.07, −0.52]	7	
Physical activity					0.42
Low: 1–12	0				
Moderate: 13–20	3	1085	−0.58 [−1.09, −0.06]	0	
High: ≥21	7	2139	−0.86 [−1.33, −0.39]	44	
**No. interventions**					
Diet					0.48
Low: 1–5	4	1571	−1.16 [−2.11, −0.20]	71	
Moderate: 6–20	6	1653	−0.81 [−1.04, −0.58]	0	
High: ≥21	0				
Physical activity					0.71
Low: 1–5	0				
Moderate: 6–20	4	1506	−0.82 [−1.06, −0.58]	0	
High: ≥21	6	1718	−0.96 [−1.67, −0.25]	53	
**Tailoring**					
Diet					-
Yes	10	3224	−0.78 [−1.12, −0.44]	31	
None	0				
Physical activity					0.17
Yes	6	2558	−0.68 [−1.01, −0.34]	30	
None	4	666	−1.37 [−2.29, −0.44]	27	
**Resource**					
Diet					0.13
Self-monitoring tool	0				
Other	7	2246	−0.90 [−1.30, −0.50]	35	
Combination	3	978	−0.36 [−0.93, 0.21]	0	
Physical activity					0.24
Self-monitoring tool	1	119	−0.41 [−1.68, 0.86]	-	
Other	6	2127	−0.96 [−1.41, −0.51]	43	
Combination	3	978	−0.36 [−0.93, 0.21]	0	

Abbreviations: GWG: gestational weight gain; MD: mean difference; TIDieR: Template for Intervention Description and Replication. a: For all MDs, the reference group was the control group. Data from cluster-randomized trials were analyzed using adjusted effective sample sizes.

**Table 4:** Subgroup analyses of combined interventions on EGWG based on TIDieR checklist.

TIDieR Intervention Characteristics	Intervention Groups (No.)	Participants	EGWG, OR (95% CI), % ^a^	*I*^2^ (%)	*p*-Value for Subgroup Differences
**Timing of Intervention Initiation**					1.00
First trimester	2	985	0.61 [0.44, 0.85]	36	
Second trimester	6	1725	0.61 [0.42, 0.90]	46	
**Theory based**					0.84
Yes	2	815	0.63 [0.43, 0.93]	0	
None	6	1895	0.60 [0.42, 0.86]	57	
**Feedback**					0.45
Yes	7	2080	0.59 [0.42, 0.82]	49	
None	1	630	0.70 [0.51, 0.96]	-	
**Delivery Mode**					
Diet					0.28
Face to face	3	1451	0.72 [0.47, 1.12]	64	
Remote	0	0	0		
Face-to-face and remote	5	1259	0.54 [0.41, 0.72]	0	
Physical activity					0.28
Face to face	3	1451	0.72 [0.47, 1.12]	64	
Remote	0	0	0		
Face-to-face and remote	5	1259	0.54 [0.41, 0.72]	0	
**Intervention format**					
Diet					-
Individual	8	2710	0.63 [0.49, 0.81]	40	
Group	0				
Individual and Group	0				
Physical activity					0.09
Individual	7	2653	0.67 [0.54, 0.83]	30	
Group	0				
Individual and Group	1	57	0.19 [0.05, 0.79]	-	
**Provider**					
Diet					0.51
Nutritionist	4	900	0.57 [0.39, 0.82]	1	
Other	4	1810	0.67 [0.48, 0.93]	59	
Physical activity					0.09
Sports health professionals	1	57	0.19 [0.05, 0.79]	-	
Other	7	2653	0.67 [0.54, 0.83]	30	
**Location**					
Diet					0.09
Healthcare facility	7	2653	0.67 [0.54, 0.83]	30	
Other	1	57	0.19 [0.05, 0.79]	-	
Physical activity					0.09
Healthcare facility	7	2653	0.67 [0.54, 0.83]	30	
Other	1	57	0.19 [0.05, 0.79]	-	
**Duration, wk**					
Diet					0.19
Low: 1–12	1	57	0.19 [0.05, 0.79]	-	
Moderate: 13–20	2	690	0.58 [0.37, 0.91]	0	
High: ≥21	5	1963	0.69 [0.53, 0.90]	41	
Physical activity					0.70
Low: 1–12	0				
Moderate: 13–20	2	690	0.58 [0.37, 0.91]	0	
High: ≥21	6	2020	0.64 [0.47, 0.87]	51	
**No. interventions**					
Diet					0.74
Low: 1–5	3	1176	0.57 [0.29, 1.11]	78	
Moderate: 6–20	5	1534	0.64 [0.51, 0.81]	0	
High: ≥21	0				
Physical activity					0.58
Low: 1–5	0				
Moderate: 6–20	4	1506	0.65 [0.52, 0.83]	0	
High: ≥21	4	1204	0.54 [0.30, 1.00]	70	
**Tailoring**					
Diet					-
Yes	8	2710	0.63 [0.49, 0.81]	40	
None					
Physical activity					0.27
Yes	5	2439	0.68 [0.52, 0.88]	47	
None	3	271	0.45 [0.23, 0.88]	21	
**Resource**					
Diet					0.21
Self-monitoring tool	0				
Other	5	1732	0.57 [0.44, 0.76]	23	
Combination	3	978	0.78 [0.53, 1.15]	29	
Physical activity					0.21
Self-monitoring tool	0				
Other	5	1732	0.57 [0.44, 0.76]	23	
Combination	3	978	0.78 [0.53, 1.15]	29	

Abbreviations: EGWG: excessive gestational weight gain; OR: Odds Ratio; CI: Confidence Interval; TIDieR: Template for Intervention Description and Replication. a: For all ORs, the reference group was the control group. Data from cluster-randomized trials were analyzed using adjusted effective sample sizes.

## Data Availability

No new data were created or analyzed in this study.

## Supplementary Materials

The following supporting information can be downloaded at: https://www.mdpi.com/article/10.3390/healthcare14081035/s1. Table S1: PRISMA checklist; File S1. Search strategy; File S2: The excluded studies with reasons; File S3: The titles of the included studies; File S4: Adjustment of cluster-randomized trials using the design effect method; File S5: Summary of risk of bias assessment; File S6: Supplementary figures; File S7: Summary of the results of sensitivity analyses; File S8: GRADE evidence of outcomes.
