# Supplementary material for: Effects of Combined Diet and Physical Activity on Gestational Weight Gain in Low-Risk Pregnant Women Based on the TIDieR Checklist: A Systematic Review and Meta-Analysis
Source: Healthcare (Basel). 2026 Apr 14;14(8):1035. doi: 10.3390/healthcare14081035 (PMC13115787; doi:10.3390/healthcare14081035)
Supplement: Supplementary file 1 [file healthcare-14-01035-s001.zip › Supplementary File S1. Search strategy.pdf]

Supplementary File S1. Search strategy

1. Pubmed

	Search strategies	Results
#1	"Pregnant People"[Mesh] OR "Pregnancy"[Mesh] OR "Gravidity"[Mesh]	1,081,959
#2	"pregnan*"[Title/Abstract] OR "gestation*"[Title/Abstract] OR "pregnant wom*n"[Title/Abstract] OR "mom"[Title/Abstract] OR "mother"[Title/Abstract] OR "prenatal"[Title/Abstract] OR "maternal"[Title/Abstract] OR "antenatal"[Title/Abstract]	1,130,239
#3	#1 OR #2	1,532,216
#4	"Motor Activity"[Mesh] OR "Sports"[Mesh] OR "Swimming"[Mesh] OR "Walking"[Mesh]	524,472
#5	"exercis*"[Title/Abstract] OR "physical activit*"[Title/Abstract] OR "moderate intensity activit*"[Title/Abstract] OR "high intensity activit*"[Title/Abstract] OR "vigorous intensity activit*"[Title/Abstract] OR "moderate vigorous activit*"[Title/Abstract] OR "moderate to vigorous activit*"[Title/Abstract] OR "outdoor activit*"[Title/Abstract] OR "indoor activit*"[Title/Abstract] OR "fitness"[Title/Abstract] OR "gymnas*"[Title/Abstract] OR "sport*"[Title/Abstract] OR "swimming"[Title/Abstract] OR "walking"[Title/Abstract] OR "dancing"[Title/Abstract] OR "jogging"[Title/Abstract] OR "running"[Title/Abstract] OR "cycling"[Title/Abstract] OR "bicycle"[Title/Abstract] OR "bicycling"[Title/Abstract] OR "physical training"[Title/Abstract] OR "strength training"[Title/Abstract] OR "weight training"[Title/Abstract] OR "resistance training"[Title/Abstract] OR "balance training"[Title/Abstract] OR "aerobic training"[Title/Abstract] OR "anaerobic training"[Title/Abstract] OR "endurance training"[Title/Abstract] OR "muscle training"[Title/Abstract]	1,028,286
#6	#4 OR #5	1,216,357
#7	"Diet, Food, and Nutrition"[Mesh]	1,400,994
#8	"diet"[Title/Abstract] OR "dietary"[Title/Abstract] OR "nutrition"[Title/Abstract] OR "diet service*"[Title/Abstract] OR "dietary service*"[Title/Abstract] OR "diet therap*"[Title/Abstract] OR "dietary therap*"[Title/Abstract] OR "diet restriction*"[Title/Abstract] OR "dietary restriction*"[Title/Abstract] OR "diet modification*"[Title/Abstract] OR "dietary modification*"[Title/Abstract] OR "diet counseling"[Title/Abstract] OR "dietary counseling"[Title/Abstract] OR "diet consultation"[Title/Abstract] OR "dietary consultation"[Title/Abstract] OR "diet management"[Title/Abstract] OR "dietary management"[Title/Abstract] OR "diet intervention*"[Title/Abstract] OR "dietary intervention*"[Title/Abstract] OR "nutritional counselling"[Title/Abstract] OR "nutritional consultation"[Title/Abstract]	890,946
#9	#7 OR #8	1,846,441
#10	#3 AND #6 AND #9	7,872
#11	Filters: Randomized Controlled Trial, from 1900/1/1 - 2026/3/16	561

2. Embase

	Search strategies	Results
#1	'pregnant woman'/exp OR 'pregnancy'/exp	1,321,324
#2	gravidity:ti,kw OR pregnan*:ti,kw OR gestation*:ti,kw OR 'pregnant wom*n':ti,kw OR mom:ti,kw OR mother:ti,kw OR prenatal:ti,kw OR maternal:ti,kw OR antenatal:ti,kw OR gravidity:ti,kw	742,682
#3	#1 OR #2	1,467,288
#4	'motor activity'/exp OR 'sport'/exp OR 'physical activity'/exp	1,454,783
#5	exercis*:ti,kw OR 'physical activit*':ti,kw OR 'moderate intensity activit*':ti,kw OR 'high intensity activit*':ti,kw OR 'vigorous intensity activit*':ti,kw OR 'moderate-vigorous activit*':ti,kw OR 'moderate to vigorous activit*':ti,kw OR 'outdoor activit*':ti,kw OR 'indoor activit*':ti,kw OR fitness:ti,kw OR gymnas*:ti,kw OR sport*:ti,kw OR swimming:ti,kw OR walking:ti,kw OR dancing:ti,kw OR jogging:ti,kw OR running:ti,kw OR cycling:ti,kw OR bicycle:ti,kw OR bicycling:ti,kw OR 'physical training':ti,kw OR 'strength training':ti,kw OR 'weight training':ti,kw OR 'resistance training':ti,kw OR 'balance training':ti,kw OR 'aerobic training':ti,kw OR 'anaerobic training':ti,kw OR 'endurance training':ti,kw OR 'muscle training':ti,kw	563,270
#6	#4 OR #5	1,772,422
#7	'nutrition'/exp	3,288,961
#8	diet:ti,kw OR dietary:ti,kw OR nutrition:ti,kw OR 'diet service*':ti,kw OR 'dietary service*':ti,kw OR 'diet therap*':ti,kw OR 'dietary therap*':ti,kw OR 'diet restriction*':ti,kw OR 'dietary restriction*':ti,kw OR 'diet modification*':ti,kw OR 'dietary modification*':ti,kw OR 'diet counseling':ti,kw OR 'dietary counseling':ti,kw OR 'diet consultation':ti,kw OR 'dietary consultation':ti,kw OR 'diet management':ti,kw OR 'dietary management':ti,kw OR 'diet intervention*':ti,kw OR 'dietary intervention*':ti,kw OR 'nutritional counselling':ti,kw OR 'nutritional consultation':ti,kw	471,379
#9	#7 OR #8	3,347,335
#10	#3 AND #6 AND #9	9,878
#11	#10 AND 'randomized controlled trial'/de	1,177
#12	#11 AND [01-01-1900]/sd NOT [17-03-2026]/sd	1,176

3. CINAHL Plus

	Search strategies	Results
#1	MH "Pregnancy"	237,129
#2	SU (pregnan* OR gestation* OR "pregnant wom*n" OR mom OR mother OR prenatal OR maternal OR antenatal)	332,260
#3	#1 OR #2	332,260
#4	MH ("Sports" OR "Exercise" OR "Physical Activity" OR "Physical Fitness" OR "Motor Activity")	157,040
#5	SU (exercis* OR "physical activit*" OR "moderate intensity activit*" OR "high intensity activit*" OR "vigorous intensity activit*" OR "moderate-vigorous activit*" OR "moderate to vigorous activit*" OR "outdoor activit*" OR "indoor activit*" OR fitness OR gymnas* OR sport* OR swimming OR walking OR dancing OR jogging OR running OR cycling OR bicycle OR bicycling OR "physical training" OR "strength training" OR "weight training" OR "resistance training" OR "balance training" OR "aerobic training" OR "anaerobic training" OR "endurance training" OR "muscle training")	318,451
#6	#4 OR #5	328,328
#7	(MH "Nutrition Services") OR (MH "Nutrition Education")	12,679
#8	SU (diet OR dietary OR nutrition OR "diet service*" OR "dietary service*" OR "diet therap*" OR "dietary therap*" OR "diet restriction*" OR "dietary restriction*" OR "diet modification*" OR "dietary modification*" OR "diet counseling" OR "dietary counseling" OR "diet consultation" OR "dietary consultation" OR "diet management" OR "dietary management" OR "diet intervention*" OR "dietary intervention*" OR "nutritional counselling" OR "nutritional consultation")	262,418
#9	#7 OR #8	262,418
#10	#3 AND #6 AND #9	1,272
#11	Filters: Randomized Controlled Trials;01/01/1900 - 03/16/2026	123

4. Web of Science

	Search strategies	Results
#1	TS=(pregnan* OR gestation* OR "pregnant wom*n" OR mom OR mother OR prenatal OR maternal OR antenatal)	1,736,771
#2	TS=(exercis* OR "physical activit*" OR "moderate intensity activit*" OR "high intensity activit*" OR "vigorous intensity activit*" OR "moderate-vigorous activit*" OR "moderate to vigorous activit*" OR "outdoor activit*" OR "indoor activit*" OR fitness OR gymnas* OR sport* OR swimming OR walking OR dancing OR jogging OR running OR cycling OR bicycle OR bicycling OR "physical training" OR "strength training" OR "weight training" OR "resistance training" OR "balance training" OR "aerobic training" OR "anaerobic training" OR "endurance training" OR "muscle training")	5,257,819
#3	TS=(diet OR dietary OR nutrition OR "diet service*" OR "dietary service*" OR "diet therap*" OR "dietary therap*" OR "diet restriction*" OR "dietary restriction*" OR "diet modification*" OR "dietary modification*" OR "diet counseling" OR "dietary counseling" OR "diet consultation" OR "dietary consultation" OR "diet management" OR "dietary management" OR "diet intervention*" OR "dietary intervention*" OR "nutritional counselling" OR "nutritional consultation")	3,187,929
#4	TS=("randomi$ed controlled trial" OR "controlled trial, randomi$ed" OR "randomi$ed controlled study" OR "trial, randomi$ed controlled" OR placebo)	648,706
#5	#1 AND #2 AND #3 AND #4	1,226
#6	#5 AND DOP=(1900-01-01/2026-03-16) and Article (Document Types)	1,196

5. The Cochrane Library

	Search strategies	Results
#1	MeSH descriptor: [Pregnant Women] explode all trees OR MeSH descriptor: [Pregnancy] explode all trees OR MeSH descriptor: [Gravidity] explode all trees	34,279
#2	(pregnan* OR gestation* OR "pregnant wom*n" OR mom OR mother OR prenatal OR maternal OR antenatal):ti,ab,kw	129,469
#3	#1 OR #2	129,701
#4	MeSH descriptor: [Motor Activity] explode all trees OR MeSH descriptor: [Sports] explode all trees OR MeSH descriptor: [Swimming] explode all trees OR MeSH descriptor: [Walking] explode all trees	55,230
#5	(exercis* OR "physical activit*" OR "moderate intensity activit*" OR "high intensity activit*" OR "vigorous intensity activit*" OR "moderate-vigorous activit*" OR "moderate to vigorous activit*" OR "outdoor activit*" OR "indoor activit*" OR fitness OR gymnas* OR sport* OR swimming OR walking OR dancing OR jogging OR running OR cycling OR bicycle OR bicycling OR "physical training" OR "strength training" OR "weight training" OR "resistance training" OR "balance training" OR "aerobic training" OR "anaerobic training" OR "endurance training" OR "muscle training"):ti,ab,kw	371,886
#6	#4 OR #5	374,593
#7	MeSH descriptor: [Diet, Food, and Nutrition] explode all trees	78,287
#8	(diet OR dietary OR nutrition OR "diet service*" OR "dietary service*" OR "diet therap*" OR "dietary therap*" OR "diet restriction*" OR "dietary restriction*" OR "diet modification*" OR "dietary modification*" OR "diet counseling" OR "dietary counseling" OR "diet consultation" OR "dietary consultation" OR "diet management" OR "dietary management" OR "diet intervention*" OR "dietary intervention*" OR "nutritional counselling" OR "nutritional consultation"):ti,ab,kw	157,129
#9	#7 OR #8	180,725
#10	MeSH descriptor: [Randomized Controlled Trials as Topic] explode all trees	64,428
#11	("randomized controlled trial" OR "controlled trial, randomized" OR "randomized controlled study" OR "trial, randomized controlled" OR placebo):ti,ab,kw	1,059,472
#12	#10 OR #11	1,059,472
#13	#3 AND #6 AND #9 AND #12	2,005
#14	Trials matching #11(to 2026/03/16)	1,941

6. WHO ICTRP

	Search strategies	Results
#1	Condition: pregnan* OR gestation* OR "pregnant women" OR "pregnant woman" OR mom OR mother OR prenatal OR maternal OR antenatal	18,248
#2	Intervention: (exercis* OR "physical activit*" OR "outdoor activit*" OR "indoor activit*" OR fitness OR gymnas* OR sport* OR swimming OR walking OR dancing OR jogging OR running OR cycling OR bicycle OR bicycling OR "physical training" OR "strength training" OR "weight training" OR "resistance training" OR "balance training" OR "aerobic training" OR "anaerobic training" OR "endurance training" OR "muscle training") AND (diet OR dietary OR nutrition OR "diet service*" OR "dietary service*" OR "diet therap*" OR "dietary therap*" OR "diet restriction*" OR "dietary restriction*" OR "diet modification*" OR "dietary modification*" OR "diet counseling" OR "dietary counseling" OR "diet consultation" OR "dietary consultation" OR "diet management" OR "dietary management" OR "diet intervention*" OR "dietary intervention*" OR "nutritional counselling" OR "nutritional consultation")	150,829
#3	#1 AND #2	56

7. ClinicalTrials.gov

	Search strategies	Results
#1	Condition/disease: pregnan* OR gestation* OR "pregnant women" OR "pregnant woman" OR mom OR mother OR prenatal OR maternal OR antenatal	44,175
#2	Intervention: (exercis* OR "physical activit*" OR "outdoor activit*" OR "indoor activit*" OR fitness OR gymnas* OR sport* OR swimming OR walking OR dancing OR jogging OR running OR cycling OR bicycle OR bicycling OR "physical training" OR "strength training" OR "weight training" OR "resistance training" OR "balance training" OR "aerobic training" OR "anaerobic training" OR "endurance training" OR "muscle training") AND (diet OR dietary OR nutrition OR "diet service*" OR "dietary service*" OR "diet therap*" OR "dietary therap*" OR "diet restriction*" OR "dietary restriction*" OR "diet modification*" OR "dietary modification*" OR "diet counseling" OR "dietary counseling" OR "diet consultation" OR "dietary consultation" OR "diet management" OR "dietary management" OR "diet intervention*" OR "dietary intervention*" OR "nutritional counselling" OR "nutritional consultation")	2,141
#3	#1 AND #2	140
#4	Filters: Female participants AND (18 - 64) AND Interventional studies	124

8. ProQuest Dissertations & Theses

	Search strategies	Results
#1	subject(pregnan* OR gestation* OR "pregnant women" OR mom OR mother OR prenatal OR maternal OR antenatal)	757,438
#2	MAINSUBJECT.EXACT("Exercise" OR "Sports")	425,015
#3	subject(exercise OR "physical activity" OR "moderate intensity activity" OR "high intensity activity" OR "vigorous intensity activity" OR "moderate-vigorous activity" OR "moderate to vigorous activity" OR "outdoor activity" OR "indoor activity" OR fitness OR gymnas* OR sport* OR swimming OR walking OR dancing OR jogging OR running OR cycling OR bicycle OR bicycling OR "physical training" OR "strength training" OR "weight training" OR "resistance training" OR "balance training" OR "aerobic training" OR "anaerobic training" OR "endurance training" OR "muscle training")	5,155,598
#4	#2 OR #3	5,155,598
#5	MAINSUBJECT.EXACT("Diet" OR "Nutrition therapy" OR "Nutrition education")	285,449
#6	subject (diet OR dietary OR nutrition OR "diet service*" OR "dietary service*" OR "diet therap*" OR "dietary therap*" OR "diet restriction*" OR "dietary restriction*" OR "diet modification*" OR "dietary modification*" OR "diet counseling" OR "dietary counseling" OR "diet consultation" OR "dietary consultation" OR "diet management" OR "dietary management" OR "diet intervention*" OR "dietary intervention*" OR "nutritional counselling" OR "nutritional consultation")	2,217,393
#7	#5 OR #6	2,421,215
#8	MAINSUBJECT.EXACT("Clinical trials") OR subject("randomized controlled trial" OR "controlled trial, randomized" OR "randomized controlled study" OR "trial, randomized controlled" OR "placebo")	1,361,695
#9	#1 AND #4 AND #7 AND #8	214
#10	#9 AND pd(19000101-20260316)	258
