# Supplementary material for: Effects of Combined Diet and Physical Activity on Gestational Weight Gain in Low-Risk Pregnant Women Based on the TIDieR Checklist: A Systematic Review and Meta-Analysis
Source: Healthcare (Basel). 2026 Apr 14;14(8):1035. doi: 10.3390/healthcare14081035 (PMC13115787; doi:10.3390/healthcare14081035)
Supplement: Supplementary file 1 [file healthcare-14-01035-s001.zip › Supplementary File S2. The excluded studies with reasons.pdf]

Supplementary File S2. The excluded studies with reasons

1. Reason 1: ineligible population（n=15）

[1] Huang TT, Yeh CY, Tsai YC. A diet and physical activity intervention for preventing weight retention among Taiwanese childbearing women: a randomised controlled trial. *Midwifery*. 2011;27(2):257-264. doi:10.1016/j.midw.2009.06.009

[2] Jackson RA, Stotland NE, Caughey AB, Gerbert B. Improving diet and exercise in pregnancy with Video Doctor counseling: a randomized trial. *Patient Educ Couns*. 2011;83(2):203-209. doi:10.1016/j.pec.2010.05.019

[3] Hui A, Back L, Ludwig S, et al. Lifestyle intervention on diet and exercise reduced excessive gestational weight gain in pregnant women under a randomised controlled trial. *BJOG*. 2012;119(1):70-77. doi:10.1111/j.1471-0528.2011.03184.x

[4] Althuizen E, van der Wijden CL, van Mechelen W, Seidell JC, van Poppel MN. The effect of a counselling intervention on weight changes during and after pregnancy: a randomised trial. *BJOG*. 2013;120(1):92-99. doi:10.1111/1471-0528.12014

[5] Thomson JL, Tussing-Humphreys LM, Goodman MH, Olender SE. Gestational Weight Gain: Results from the Delta Healthy Sprouts Comparative Impact Trial. *J Pregnancy*. 2016;2016:5703607. doi:10.1155/2016/5703607

[6] Sanda B, Vistad I, Sagedal LR, Haakstad LAH, Lohne-Seiler H, Torstveit MK. What is the effect of physical activity on duration and mode of delivery? Secondary analysis from the Norwegian Fit for Delivery trial. *Acta Obstet Gynecol Scand*. 2018;97(7):861-871. doi:10.1111/aogs.13351

[7] Broekhuizen K, Simmons D, Devlieger R, et al. Cost-effectiveness of healthy eating and/or physical activity promotion in pregnant women at increased risk of gestational diabetes mellitus: economic evaluation alongside the DALI study, a European multicenter randomized controlled trial. *Int J Behav Nutr Phys Act*. 2018;15(1):23. Published 2018 Mar 14. doi:10.1186/s12966-018-0643-y

[8] Chan RS, Tam WH, Ho IC, et al. Randomized trial examining effectiveness of lifestyle intervention in reducing gestational diabetes in high risk Chinese pregnant women in Hong Kong. *Sci Rep*. 2018;8(1):13849. Published 2018 Sep 14. doi:10.1038/s41598-018-32285-6

[9] Coughlin JW, Martin LM, Henderson J, et al. Feasibility and acceptability of a remotely-delivered behavioural health coaching intervention to limit gestational weight gain. *Obes Sci Pract*. 2020;6(5):484-493. Published 2020 Jul 16. doi:10.1002/osp4.438

[10] Deng Y, Hou Y, Wu L, Liu Y, Ma L, Yao A. Effects of Diet and Exercise Interventions to Prevent Gestational Diabetes Mellitus in Pregnant Women With High-Risk Factors in China: A Randomized Controlled Study. *Clin Nurs Res*. 2022;31(5):836-847. doi:10.1177/10547738211055576

[11] Cao Y, Sheng J, Zhang D, et al. The role of dietary fiber on preventing gestational diabetes mellitus in an at-risk group of high triglyceride-glucose index women: a randomized controlled trial. *Endocrine*. 2023;82(3):542-549. doi:10.1007/s12020-023-03478-5

[12] Rissel C, Khanal S, Raymond J, Clements V, Leung K, Nicholl M. Piloting a Telephone Based Health Coaching Program for Pregnant Women: A Mixed Methods Study. *Matern Child Health J*. 2019;23(3):307-315. doi:10.1007/s10995-019-02735-2

[13] Asbee SM, Jenkins TR, Butler JR, White J, Elliot M, Rutledge A. Preventing excessive

weight gain during pregnancy through dietary and lifestyle counseling: a randomized controlled trial. *Obstet Gynecol*. 2009;113(2 Pt 1):305-312. doi:10.1097/AOG.0b013e318195baef
[14] Sandborg J, Söderström E, Henriksson P, et al. Effectiveness of a Smartphone App to Promote Healthy Weight Gain, Diet, and Physical Activity During Pregnancy (HealthyMoms): Randomized Controlled Trial. *JMIR Mhealth Uhealth*. 2021;9(3):e26091. Published 2021 Mar 11. doi:10.2196/26091
[15] Gesell SB, Katula JA, Strickland C, Vitolins MZ. Feasibility and Initial Efficacy Evaluation of a Community-Based Cognitive-Behavioral Lifestyle Intervention to Prevent Excessive Weight Gain During Pregnancy in Latina Women. Matern Child Health J. 2015;19(8):1842-1852. doi:10.1007/s10995-015-1698-x

2. Reason 2: ineligible intervention（n=4）

[1] Arthur C, Di Corleto E, Ballard E, Kothari A. A randomized controlled trial of daily weighing in pregnancy to control gestational weight gain. *BMC Pregnancy Childbirth*. 2020;20(1):223. Published 2020 Apr 16. doi:10.1186/s12884-020-02884-1
[2] Vadsaria K, Nuruddin R, Mohammed N, Azam I, Sayani S. Efficacy of a Personalized mHealth App in Improving Micronutrient Supplement Use Among Pregnant Women in Karachi, Pakistan: Parallel-Group Randomized Controlled Trial. *J Med Internet Res*. 2025;27:e67166. Published 2025 Apr 9. doi:10.2196/67166
[3] Ruchat SM, Davenport MH, Giroux I, et al. Nutrition and exercise reduce excessive weight gain in normal-weight pregnant women. Med Sci Sports Exerc. 2012;44(8):1419-1426. doi:10.1249/MSS.0b013e31825365f1
[4] Pawalia A, Kulandaivelan S, Savant S, et al. Effect of behavioural interventions for obesity prevention in pregnancy on the adequacy of gestational weight gain and retention: metabolic health of Indian women. *EABR*. 2020;21(1): 35-42. doi: 10.2478/sjecr-2018-0068

3. Reason 3: ineligible outcomes（n=7）

[1] Demment MM, Graham ML, Olson CM. How an online intervention to prevent excessive gestational weight gain is used and by whom: a randomized controlled process evaluation. *J Med Internet Res*. 2014;16(8):e194. Published 2014 Aug 20. doi:10.2196/jmir.3483
[2] Huang RC, Silva D, Beilin L, et al. Feasibility of conducting an early pregnancy diet and lifestyle e-health intervention: the Pregnancy Lifestyle Activity Nutrition (PLAN) project. *J Dev Orig Health Dis*. 2020;11(1):58-70. doi:10.1017/S2040174419000400
[3] Karimipour Z, Sharifi N, Seydkhani H, Sayadi H, Jalilian M. Lifestyle intervention for gestational diabetes prevention in rural woman of Shoush city. *J Educ Health Promot*. 2021;10(1):172. Published 2021 May 31. doi:10.4103/jehp.jehp_1072_20
[4] Perreault M, Mottola MF, Atkinson SA; BHIP study team. Individualized high dairy protein + walking program supports bone health in pregnancy: a randomized controlled trial. *Am J Clin Nutr*. 2022;116(4):887-896. doi:10.1093/ajcn/nqac182
[5] Graham ML, Strawderman MS, Demment M, Olson CM. Does Usage of an eHealth Intervention Reduce the Risk of Excessive Gestational Weight Gain? Secondary Analysis From a Randomized Controlled Trial. *J Med Internet Res*. 2017;19(1):e6. Published 2017 Jan 9. doi:10.2196/jmir.6644

[6] Estevez Burns R, Hare ME, Andres A, et al. An interim analysis of a gestational weight gain intervention in military personnel and other TRICARE beneficiaries. *Obesity (Silver Spring)*. 2022;30(10):1951-1962. doi:10.1002/oby.23523
[7] Bgeginski R, Nagpal TS, Hosein K, et al. Does Delivery of a Nutrition and Exercise Intervention Simultaneously or Sequentially Prevent Excessive Gestational Weight Gain? The NELIP Trial. Med Sci Sports Exerc. 2025;57(9):2032-2039. doi:10.1249/MSS.0000000000003729

4. Reason 4: duplicates（n=1）

[1] Buckingham-Schutt L. The Behavioral Wellness in Pregnancy Study: A Theory-Based Multi-Component Intervention to Promote Appropriate Weight Gain and Healthy Lifestyle Behaviors in Previously Sedentary Pregnant Women. Order No. 10680606 ed. Iowa State University; 2017. https://www.proquest.com/dissertations-theses/behavioral-wellness-pregnancy-study-theory-based/docview/2013203328/se-2

5. Reason 5: protocol（n=1）

[1] Clements V, Leung K, Khanal S, Raymond J, Maxwell M, Rissel C. Pragmatic cluster randomised trial of a free telephone-based health coaching program to support women in managing weight gain during pregnancy: the Get Healthy in Pregnancy Trial. *BMC Health Serv Res*. 2016;16(1):454. Published 2016 Aug 30. doi:10.1186/s12913-016-1704-z

6. Reason 6: conference abstract (n=1)

[1] Flanagan E, Falkenhain K, Beyl R, et al. Gestational Weight Gain Management in Underserved Mothers: A State-Wide Randomized Trial in WIC. Obesity. 2025;33(Supplement 2):47-48. doi:10.1002/oby.70102
