# Supplementary material for: Effects of Combined Diet and Physical Activity on Gestational Weight Gain in Low-Risk Pregnant Women Based on the TIDieR Checklist: A Systematic Review and Meta-Analysis
Source: Healthcare (Basel). 2026 Apr 14;14(8):1035. doi: 10.3390/healthcare14081035 (PMC13115787; doi:10.3390/healthcare14081035)
Supplement: Supplementary file 1 [file healthcare-14-01035-s001.zip › Supplementary File S3. The titles of the included studies.pdf]

Supplementary File S3. The titles of the included studies (N=10)

[1] Polley BA, Wing RR, Sims CJ. Randomized controlled trial to prevent excessive weight gain in pregnant women. *Int J Obes Relat Metab Disord*. 2002;26(11):1494-1502. doi:10.1038/sj.ijo.0802130

[2] Phelan S, Phipps MG, Abrams B, Darroch F, Schaffner A, Wing RR. Randomized trial of a behavioral intervention to prevent excessive gestational weight gain: the Fit for Delivery Study. *Am J Clin Nutr*. 2011;93(4):772-779. doi:10.3945/ajcn.110.005306

[3] Hui AL, Back L, Ludwig S, et al. Effects of lifestyle intervention on dietary intake, physical activity level, and gestational weight gain in pregnant women with different pre-pregnancy Body Mass Index in a randomized control trial. *BMC Pregnancy Childbirth*. 2014;14:331. Published 2014 Sep 24. doi:10.1186/1471-2393-14-331

[4] Sagedal LR, Øverby NC, Bere E, et al. Lifestyle intervention to limit gestational weight gain: the Norwegian Fit for Delivery randomised controlled trial. *BJOG*. 2017;124(1):97-109. doi:10.1111/1471-0528.13862

[5] Buckingham-Schutt LM, Ellingson LD, Vazou S, Campbell CG. The Behavioral Wellness in Pregnancy study: a randomized controlled trial of a multi-component intervention to promote appropriate weight gain. *Am J Clin Nutr*. 2019;109(4):1071-1079. doi:10.1093/ajcn/nqy359

[6] Kunath J, Günther J, Rauh K, et al. Effects of a lifestyle intervention during pregnancy to prevent excessive gestational weight gain in routine care - the cluster-randomised GeliS trial. *BMC Med*. 2019;17(1):5. Published 2019 Jan 14. doi:10.1186/s12916-018-1235-z

[7] Dodd JM, Deussen AR, Louise J. A Randomised Trial to Optimise Gestational Weight Gain and Improve Maternal and Infant Health Outcomes through Antenatal Dietary, Lifestyle and Exercise Advice: The OPTIMISE Randomised Trial. *Nutrients*. 2019;11(12):2911. Published 2019 Dec 2. doi:10.3390/nu11122911

[8] Atkinson SA, Maran A, Dempsey K, Perreault M, Vanniyasingam T, Phillips SM, Hutton EK, Mottola MF, Wahoush O, Xie F, Thabane L. Be Healthy in Pregnancy (BHIP): A Randomized Controlled Trial of Nutrition and Exercise Intervention from Early Pregnancy to Achieve Recommended Gestational Weight Gain. Nutrients. 2022 Feb 15;14(4):810. doi: 10.3390/nu14040810

[9] Krebs F, Lorenz L, Nawabi F, Alayli A, Stock S. Effectiveness of a Brief Lifestyle Intervention in the Prenatal Care Setting to Prevent Excessive Gestational Weight Gain and Improve Maternal and Infant Health Outcomes. *Int J Environ Res Public Health*. 2022;19(10):5863. Published 2022 May 11. doi:10.3390/ijerph19105863

[10] Yang H, Zhao Y, Tu J, Chang Y, Xiao C. Effects of Antenatal Lifestyle Interventions in Pregnant Women with Normal Body Mass Index. *Iran J Public Health*. 2023;52(2):381-388. doi:10.18502/ijph.v52i2.11891
