# Supplementary material for: Effects of Combined Diet and Physical Activity on Gestational Weight Gain in Low-Risk Pregnant Women Based on the TIDieR Checklist: A Systematic Review and Meta-Analysis
Source: Healthcare (Basel). 2026 Apr 14;14(8):1035. doi: 10.3390/healthcare14081035 (PMC13115787; doi:10.3390/healthcare14081035)
Supplement: Supplementary file 1 [file healthcare-14-01035-s001.zip › Supplementary File S4. Adjustment of cluster-randomized trials using the design effect method.pdf]

Supplementary File S4. Adjustment of cluster-randomized trials using the design effect method

1. Methodological Approach

Cluster-randomized controlled trials were adjusted using the design effect method in accordance with the Cochrane Handbook for Systematic Reviews of Interventions [40] to account for intra-cluster correlation. The design effect was calculated as:

$$DE = 1 + (M - 1) \times ICC$$

Where: M is the average cluster size, calculated as the total number of participants divided by the number of clusters; ICC is the intracluster correlation coefficient.

For dichotomous outcomes: Both the number of participants and the number of events were divided by the design effect.

For continuous outcomes: Only the sample size was adjusted, while means and standard deviations remained unchanged.

All adjusted values were rounded to the nearest whole number for meta-analysis.

The ICC used in this study (0.5%) was derived from the trial reported by Kunath et al. [31], and was applied consistently across both cluster-randomized trials. All meta-analyses were conducted using the adjusted effective sample sizes and event counts presented above. This approach ensures appropriate weighting of cluster-randomized trials and comparability with individually randomized trials.

2. Data Calculation

(1) Study 1: Kunath J, Günther J, Rauh K, et al. Effects of a lifestyle intervention during pregnancy to prevent excessive gestational weight gain in routine care - the cluster-randomised GeliS trial. BMC Med. 2019;17(1):5. Published 2019 Jan 14. doi:10.1186/s12916-018-1235-z.

Original data: ICC=0.5%

Intervention group: Participants (n)=608; Events=208; Clusters=5

Control group: Participants (n)=624; Events=224; Clusters=5

Step 1: Average cluster size

Intervention group: M_I= 608/5=121.6; Control group: M_C=624/5=124.8

Step 2: Design effect

Intervention group: DE_I=1+(121.6−1)×0.005=1.6030

Control group: DE_C=1+(124.8−1)×0.005=1.6190

Step 3: Effective sample size and events

Intervention group: $n_{eff, I}$=608/1.6030=379.29≈379

$Events_{eff, I}$=208/1.6030=129.76≈130

Control group: $n_{eff, C}$=624/1.6190=385.42≈385

$Events_{eff, C}$=224/1.6190=138.36≈138

(2) Study 2: Krebs F, Lorenz L, Nawabi F, Alayli A, Stock S. Effectiveness of a Brief Lifestyle Intervention in the Prenatal Care Setting to Prevent Excessive Gestational Weight Gain and Improve Maternal and Infant Health Outcomes. Int J Environ Res Public Health. 2022;19(10):5863. Published 2022 May 11. doi:10.3390/ijerph19105863

Original data:

Intervention group: Participants (n)=438; Events=252; Clusters=5

Control group: Participants (n)=477; Events=233; Clusters=5

Step 1: Average cluster size

Intervention group: M_I= 477/5=95.4; Control group: M_C=438/5=87.6

Step 2: Design effect

Intervention group: DE_I=1+(95.4−1)×0.005=1.472

Control group: DE_C=1+(87.6−1)×0.005=1.433

Step 3: Effective sample size and events

Intervention group: $n_{eff, I}$=477/1.472=324.048913≈324;

$Events_{eff, I}$=233/1.472=158.2880434≈158

Control group: $n_{eff, C}$=438/1.433=305.6524773≈306;

$Events_{eff, C}$=252/1.433=175.8548499≈176
