# Supplementary material for: Effects of Combined Diet and Physical Activity on Gestational Weight Gain in Low-Risk Pregnant Women Based on the TIDieR Checklist: A Systematic Review and Meta-Analysis
Source: Healthcare (Basel). 2026 Apr 14;14(8):1035. doi: 10.3390/healthcare14081035 (PMC13115787; doi:10.3390/healthcare14081035)
Supplement: Supplementary file 1 [file healthcare-14-01035-s001.zip › Supplementary File S5. Summary of risk of bias assessment.pdf]

Supplementary File S5. Summary of risk of bias assessment

Study	Assessors	Risk of Bias																											Overall bias
		Bias Arising from the Randomization Process				Bias Due to Deviations from Intended Interventions								Bias Due to Missing Outcome Data					Bias in Measurement of the Outcome						Bias in Selection of the Reported Result				
		Q 1.1	Q 1.2	Q 1.3	Domain	Q 2.1	Q 2.2	Q 2.3	Q 2.4	Q 2.5	Q 2.6	Q 2.7	Domain	Q 3.1	Q 3.2	Q 3.3	Q 3.4	Domain	Q 4.1	Q 4.2	Q 4.3	Q 4.4	Q 4.5	Domain	Q 5.1	Q 5.2	Q 5.3	Domain	
Polley 2002	1	Y	PY	N	Low	Y	Y	PY	PY	PY	Y	NA	Some concerns	Y	NA	NA	NA	Low	N	N	PY	N	NA	Low	Y	N	N	Low	Some concerns
	2	Y	NI	N	Low	PY	PY	NI	NA	NA	Y	NA	Some concerns	Y	NA	NA	NA	Low	N	N	PY	N	NA	Low	PY	N	N	Low	Some concerns
	Final results	Y	PY	N	Low	Y	Y	PY	PY	PY	Y	NA	Some concerns	Y	NA	NA	NA	Low	N	N	PY	N	NA	Low	PY	N	N	Low	Some concerns
Phelan 2011	1	Y	Y	N	Low	Y	Y	PY	PY	PY	Y	NA	Some concerns	Y	NA	NA	NA	Low	N	N	Y	N	NA	Low	Y	N	N	Low	Some concerns
	2	Y	Y	N	Low	PY	PY	NI	NA	NA	Y	NA	Some concerns	Y	NA	NA	NA	Low	N	N	N	N	NA	Low	Y	N	N	Low	Some concerns
	Final results	Y	Y	N	Low	Y	Y	PY	PY	PY	Y	NA	Some concerns	Y	NA	NA	NA	Low	N	N	Y	N	NA	Low	Y	N	N	Low	Some concerns
Hui 2014	1	Y	Y	N	Low	Y	Y	PY	PY	PY	Y	NA	Some concerns	Y	NA	NA	NA	Low	N	N	PN	NA	NA	Low	Y	N	N	Low	Some concerns
	2	Y	Y	N	Low	Y	Y	PN	NA	NA	Y	NA	Low	Y	NA	NA	NA	Low	N	N	N	NA	NA	Low	NI	N	N	Low	Low
	Final results	Y	Y	N	Low	Y	Y	PY	PY	PY	Y	NA	Some concerns	Y	NA	NA	NA	Low	N	N	N	NA	NA	Low	Y	N	N	Low	Some concerns
Sagedal 2016	1	Y	Y	N	Low	Y	Y	PY	PY	PY	Y	NA	Some concerns	Y	NA	NA	NA	Low	N	N	PN	NA	NA	Low	Y	N	N	Low	Some concerns
	2	Y	Y	N	Low	Y	Y	PN	NA	NA	Y	NA	Low	Y	NA	NA	NA	Low	N	N	N	NA	NA	Low	Y	N	N	Low	Low
	Final results	Y	Y	N	Low	Y	Y	PY	PY	PY	Y	NA	Some concerns	Y	NA	NA	NA	Low	N	N	Y	N	NA	Low	Y	N	N	Low	Some concerns

**Buckingham-Schutt 2019**	**1**	Y	PY	N	Low	Y	Y	PY	PY	PY	Y	NA	Some concerns	Y	NA	NA	NA	Low	N	N	Y	N	NA	Low	Y	N	N	Low	Some concerns
	**2**	NI	NI	N	Low	Y	Y	PN	NA	NA	Y	NA	Low	Y	NA	NA	NA	Low	N	N	PY	N	NA	Low	Y	N	N	Low	Low
	**Final results**	Y	PY	N	Low	Y	Y	PY	PY	PY	Y	NA	Some concerns	Y	NA	NA	NA	Low	N	N	Y	N	NA	Low	Y	N	N	Low	Some concerns
**Kunath 2019**	**1**	Y	PY	N	Low	Y	Y	PY	PY	PY	Y	NA	Some concerns	Y	NA	NA	NA	Low	N	N	PN	NA	NA	Low	Y	N	N	Low	Some concerns
	**2**	PY	Y	N	Low	NI	Y	PN	NA	NA	Y	NA	Low	Y	NA	NA	NA	Low	N	N	PY	N	NA	Low	Y	N	N	Low	Low
	**Final results**	PY	Y	N	Low	Y	Y	PY	PY	PY	Y	NA	Some concerns	Y	NA	NA	NA	Low	N	N	PN	NA	NA	Low	Y	N	N	Low	Some concerns
**Dodd 2019**	**1**	Y	PY	N	Low	Y	Y	PY	PY	PY	Y	NA	Some concerns	Y	NA	NA	NA	Low	N	N	Y	N	NA	Low	Y	N	N	Low	Some concerns
	**2**	Y	Y	N	Low	Y	Y	PN	NA	NA	Y	NA	Low	Y	NA	NA	NA	Low	N	N	N	NA	NA	Low	Y	N	N	Low	Low
	**Final results**	Y	PY	N	Low	Y	Y	PY	PY	PY	Y	NA	Some concerns	Y	NA	NA	NA	Low	N	N	N	NA	NA	Low	Y	N	N	Low	Some concerns
**Atkinson 2022**	**1**	Y	PY	N	Low	Y	Y	PY	PY	PY	Y	NA	Some concerns	Y	NA	NA	NA	Low	N	N	Y	N	NA	Low	Y	N	N	Low	Some concerns
	**2**	Y	Y	N	Low	Y	Y	PN	NA	NA	Y	NA	Low	Y	NA	NA	NA	Low	N	N	NI	N	NA	Low	Y	N	N	Low	Low
	**Final results**	Y	PY	N	Low	Y	Y	PY	PY	PY	Y	NA	Some concerns	Y	NA	NA	NA	Low	N	N	PY	N	NA	Low	Y	N	N	Low	Some concerns
**Krebs 2022**	**1**	Y	PY	N	Low	Y	Y	PY	PY	PY	Y	NA	Some concerns	Y	NA	NA	NA	Low	N	N	PY	N	NA	Low	Y	N	N	Low	Some concerns
	**2**	Y	PY	N	Low	N	PY	PN	NA	NA	Y	NA	Low	Y	NA	NA	NA	Low	N	N	PY	N	NA	Low	NI	N	N	Low	Low
	**Final results**	Y	PY	N	Low	PN	PY	PY	PY	PY	Y	NA	Some concerns	Y	NA	NA	NA	Low	N	N	PY	N	NA	Low	Y	N	N	Low	Some concerns
**Yang 2023**	**1**	Y	NI	N	Some concerns	Y	Y	PY	PY	PY	Y	NA	Some concerns	Y	NA	NA	NA	Low	N	N	Y	N	NA	Low	Y	N	N	Low	Some concerns
	**2**	Y	Y	N	Low	Y	PY	PN	NA	NA	Y	NA	Low	Y	NA	NA	NA	Low	N	N	Y	N	NA	Low	Y	N	N	Low	Low
	**Final results**	Y	NI	N	Some concerns	Y	Y	PY	PY	PY	Y	NA	Some concerns	Y	NA	NA	NA	Low	N	N	Y	N	NA	Low	Y	N	N	Low	Some concerns
