# Supplementary material for: Effects of Combined Diet and Physical Activity on Gestational Weight Gain in Low-Risk Pregnant Women Based on the TIDieR Checklist: A Systematic Review and Meta-Analysis
Source: Healthcare (Basel). 2026 Apr 14;14(8):1035. doi: 10.3390/healthcare14081035 (PMC13115787; doi:10.3390/healthcare14081035)

Supplementary File S6. Supplementary figures

1. Forest plot of the effect of combined diet with physical activity on total GWG below the IOM-recommended range

Figure S1. Forest plot of the effect of diet with physical activity on the proportion of women with total GWG below the IOM-recommended range

	Intervention		Control			Risk Difference		Risk Difference
Study or Subgroup	Events	Total	Events	Total	Weight	M-H, Fixed, 95% CI	Year	M-H, Fixed, 95% CI
Polley 2002	9	30	5	31	6.8%	0.14 [-0.07, 0.35]	2002	
Phelan 2011	13	92	12	94	20.6%	0.01 [-0.08, 0.11]	2011	
Buckingham-Schutt 2019	1	12	2	16	3.0%	-0.04 [-0.27, 0.18]	2019	
Dodd 2019	160	316	162	313	69.6%	-0.01 [-0.09, 0.07]	2019	
**Total (95% CI)**		**450**		**454**	**100.0%**	**0.00 [-0.06, 0.06]**		
Total events	183		181					

Heterogeneity: Chi² = 1.94, df = 3 (P = 0.58); I² = 0%

Test for overall effect: Z = 0.10 (P = 0.92)

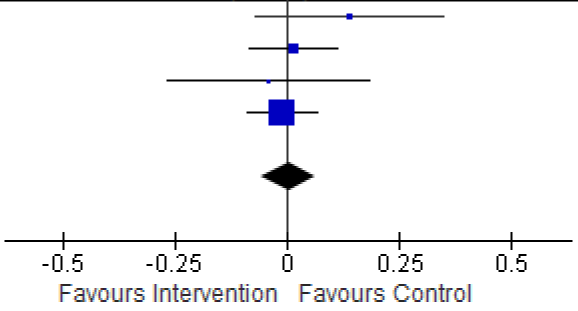

2. Forest plot of subgroup analyses of combined diet with physical activity on total GWG based on TIDieR checklist

Figure S2. Forest plot of the effect of **Timing of Intervention Initiation** on total GWG

Study or Subgroup	Intervention Mean	Intervention SD	Intervention Total	Control Mean	Control SD	Control Total	Weight	Mean Difference IV, Random, 95% CI	Year
**1.2.1 First trimester**									
Krebs 2022	14.6	1.66	324	15.5	1.71	306	31.5%	-0.90 [-1.16, -0.64]	2022
Yang 2023	14.52	4.52	178	15.82	4.35	177	10.0%	-1.30 [-2.22, -0.38]	2023
**Subtotal (95% CI)**			**502**			**483**	**41.5%**	**-0.93 [-1.18, -0.68]**	
Heterogeneity: Tau² = 0.00; Chi² = 0.67, df = 1 (P = 0.41); I² = 0%									
Test for overall effect: Z = 7.20 (P < 0.00001)									
**1.2.2 Second trimester**									
Polley 2002	15.4	7.1	30	16.4	4.8	31	1.2%	-1.00 [-4.05, 2.05]	2002
Phelan 2011	15.3	4.4	92	16.2	4.6	94	5.8%	-0.90 [-2.19, 0.39]	2011
Hui 2014	12.9	3.72	30	16.23	4.38	27	2.4%	-3.33 [-5.45, -1.21]	2014
Sagedal 2016	14.7	5.1	189	15.8	5.4	206	8.4%	-1.10 [-2.14, -0.06]	2016
Kunath 2019	14.6	4.5	379	14.8	4.6	385	16.1%	-0.20 [-0.85, 0.45]	2019
Buckingham-Schutt 2019	11.8	3.3	12	12.7	4.8	16	1.2%	-0.90 [-3.90, 2.10]	2019
Dodd 2019	11.32	3.96	316	11.7	3.78	313	17.4%	-0.38 [-0.98, 0.22]	2019
Atkinson 2022	11.02	3.4	58	11.43	3.67	61	6.0%	-0.41 [-1.68, 0.86]	2022
**Subtotal (95% CI)**			**1106**			**1133**	**58.5%**	**-0.66 [-1.14, -0.19]**	
Heterogeneity: Tau² = 0.12; Chi² = 9.58, df = 7 (P = 0.21); I² = 27%									
Test for overall effect: Z = 2.72 (P = 0.006)									
**Total (95% CI)**			**1608**			**1616**	**100.0%**	**-0.78 [-1.12, -0.44]**	
Heterogeneity: Tau² = 0.08; Chi² = 13.04, df = 9 (P = 0.16); I² = 31%									
Test for overall effect: Z = 4.53 (P < 0.00001)									
Test for subgroup differences: Chi² = 0.96, df = 1 (P = 0.33), I² = 0%									

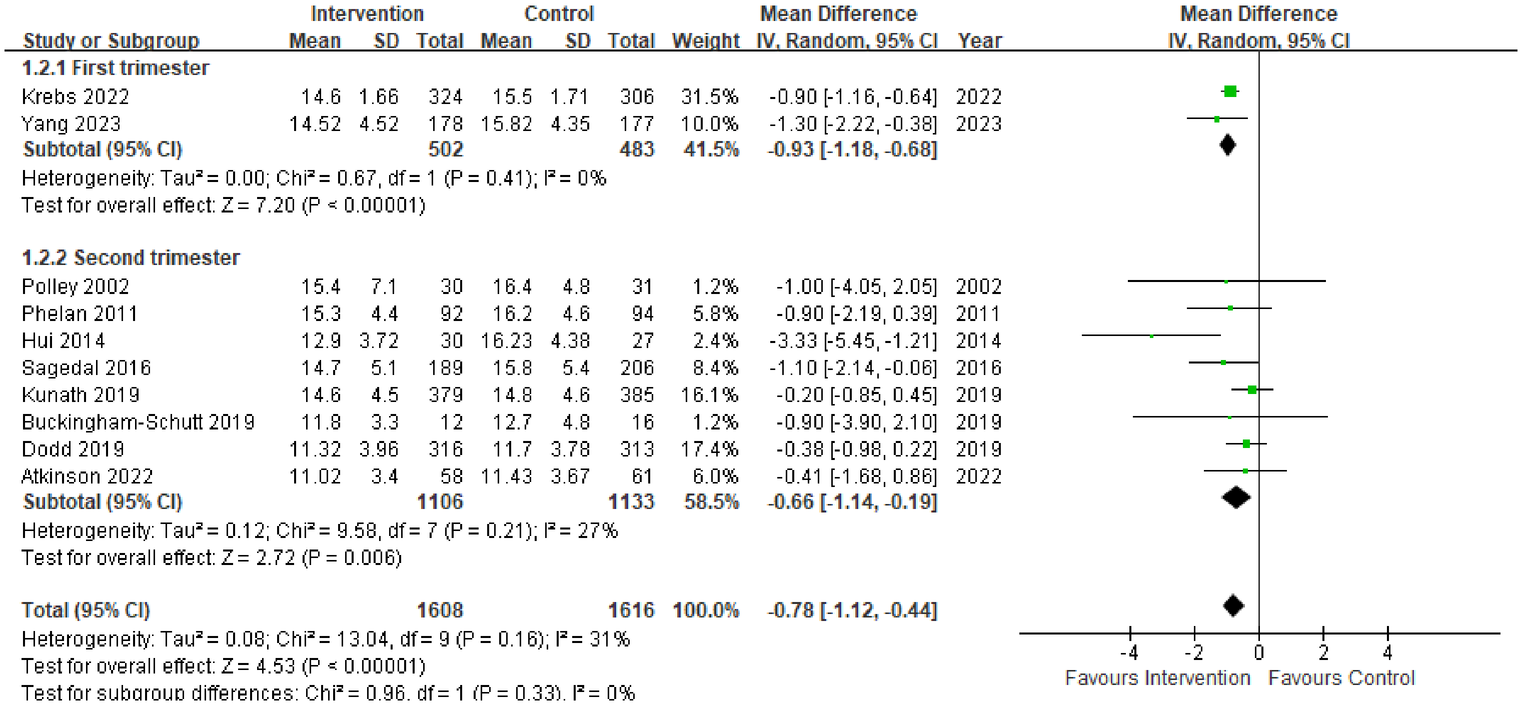

Figure S3. Forest plot of the effect of **Theory** on total GWG

Study or Subgroup	Intervention Mean	Intervention SD	Intervention Total	Control Mean	Control SD	Control Total	Weight	Mean Difference IV, Random, 95% CI	Year
**1.3.1 Theory based**									
Phelan 2011	15.3	4.4	92	16.2	4.6	94	5.8%	-0.90 [-2.19, 0.39]	2011
Dodd 2019	11.32	3.96	316	11.7	3.78	313	17.4%	-0.38 [-0.98, 0.22]	2019
**Subtotal (95% CI)**			**408**			**407**	**23.2%**	**-0.47 [-1.02, 0.07]**	
Heterogeneity: Tau² = 0.00; Chi² = 0.51, df = 1 (P = 0.48); I² = 0%									
Test for overall effect: Z = 1.69 (P = 0.09)									
**1.3.2 Non-theory based**									
Polley 2002	15.4	7.1	30	16.4	4.8	31	1.2%	-1.00 [-4.05, 2.05]	2002
Hui 2014	12.9	3.72	30	16.23	4.38	27	2.4%	-3.33 [-5.45, -1.21]	2014
Sagedal 2016	14.7	5.1	189	15.8	5.4	206	8.4%	-1.10 [-2.14, -0.06]	2016
Kunath 2019	14.6	4.5	379	14.8	4.6	385	16.1%	-0.20 [-0.85, 0.45]	2019
Buckingham-Schutt 2019	11.8	3.3	12	12.7	4.8	16	1.2%	-0.90 [-3.90, 2.10]	2019
Krebs 2022	14.6	1.66	324	15.5	1.71	306	31.5%	-0.90 [-1.16, -0.64]	2022
Atkinson 2022	11.02	3.4	58	11.43	3.67	61	6.0%	-0.41 [-1.68, 0.86]	2022
Yang 2023	14.52	4.52	178	15.82	4.35	177	10.0%	-1.30 [-2.22, -0.38]	2023
**Subtotal (95% CI)**			**1200**			**1209**	**76.8%**	**-0.88 [-1.30, -0.45]**	
Heterogeneity: Tau² = 0.11; Chi² = 10.88, df = 7 (P = 0.14); I² = 36%									
Test for overall effect: Z = 4.06 (P < 0.0001)									
**Total (95% CI)**			**1608**			**1616**	**100.0%**	**-0.78 [-1.12, -0.44]**	
Heterogeneity: Tau² = 0.08; Chi² = 13.04, df = 9 (P = 0.16); I² = 31%									
Test for overall effect: Z = 4.53 (P < 0.00001)									
Test for subgroup differences: Chi² = 1.31, df = 1 (P = 0.25), I² = 23.7%									

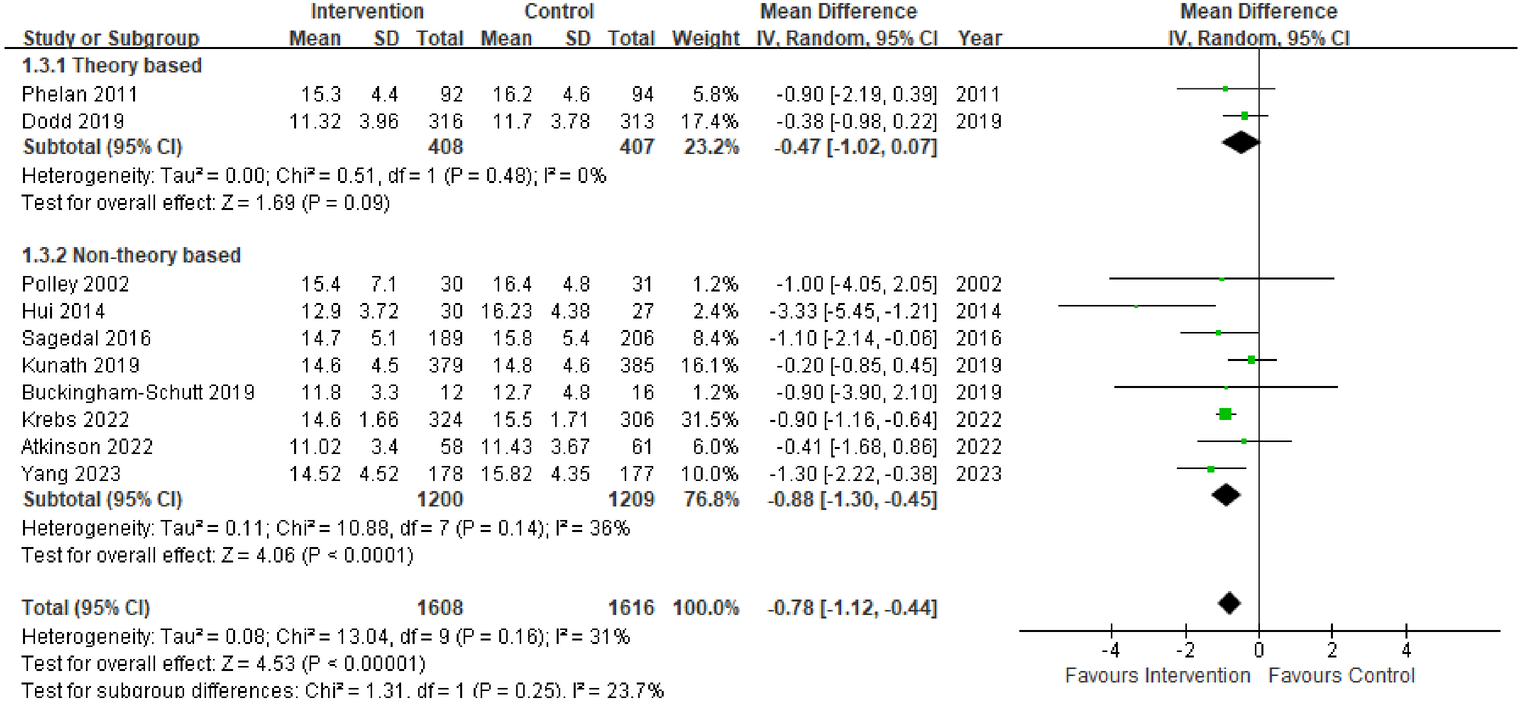

Figure S4. Forest plot of the effect of **Feedback** on total GWG

Study or Subgroup	Intervention Mean	Intervention SD	Intervention Total	Control Mean	Control SD	Control Total	Weight	Mean Difference IV, Random, 95% CI	Year
**1.12.1 With feedback**									
Polley 2002	15.4	7.1	30	16.4	4.8	31	1.2%	-1.00 [-4.05, 2.05]	2002
Phelan 2011	15.3	4.4	92	16.2	4.6	94	5.8%	-0.90 [-2.19, 0.39]	2011
Hui 2014	12.9	3.72	30	16.23	4.38	27	2.4%	-3.33 [-5.45, -1.21]	2014
Buckingham-Schutt 2019	11.8	3.3	12	12.7	4.8	16	1.2%	-0.90 [-3.90, 2.10]	2019
Dodd 2019	11.32	3.96	316	11.7	3.78	313	17.4%	-0.38 [-0.98, 0.22]	2019
Kunath 2019	14.6	4.5	379	14.8	4.6	385	16.1%	-0.20 [-0.85, 0.45]	2019
Yang 2023	14.52	4.52	178	15.82	4.35	177	10.0%	-1.30 [-2.22, -0.38]	2023
**Subtotal (95% CI)**			**1037**			**1043**	**54.1%**	**-0.81 [-1.40, -0.22]**	
Heterogeneity: $Tau^2 = 0.24$; $Chi^2 = 10.86$, df = 6 (P = 0.09); $I^2 = 45\%$									
Test for overall effect: Z = 2.70 (P = 0.007)									
**1.12.2 Without feedback**									
Sagedal 2016	14.7	5.1	189	15.8	5.4	206	8.4%	-1.10 [-2.14, -0.06]	2016
Krebs 2022	14.6	1.66	324	15.5	1.71	306	31.5%	-0.90 [-1.16, -0.64]	2022
Atkinson 2022	11.02	3.4	58	11.43	3.67	61	6.0%	-0.41 [-1.68, 0.86]	2022
**Subtotal (95% CI)**			**571**			**573**	**45.9%**	**-0.89 [-1.14, -0.64]**	
Heterogeneity: $Tau^2 = 0.00$; $Chi^2 = 0.71$, df = 2 (P = 0.70); $I^2 = 0\%$									
Test for overall effect: Z = 6.99 (P < 0.00001)									
**Total (95% CI)**			**1608**			**1616**	**100.0%**	**-0.78 [-1.12, -0.44]**	
Heterogeneity: $Tau^2 = 0.08$; $Chi^2 = 13.04$, df = 9 (P = 0.16); $I^2 = 31\%$									
Test for overall effect: Z = 4.53 (P < 0.00001)									
Test for subgroup differences: $Chi^2 = 0.06$, df = 1 (P = 0.81), $I^2 = 0\%$									

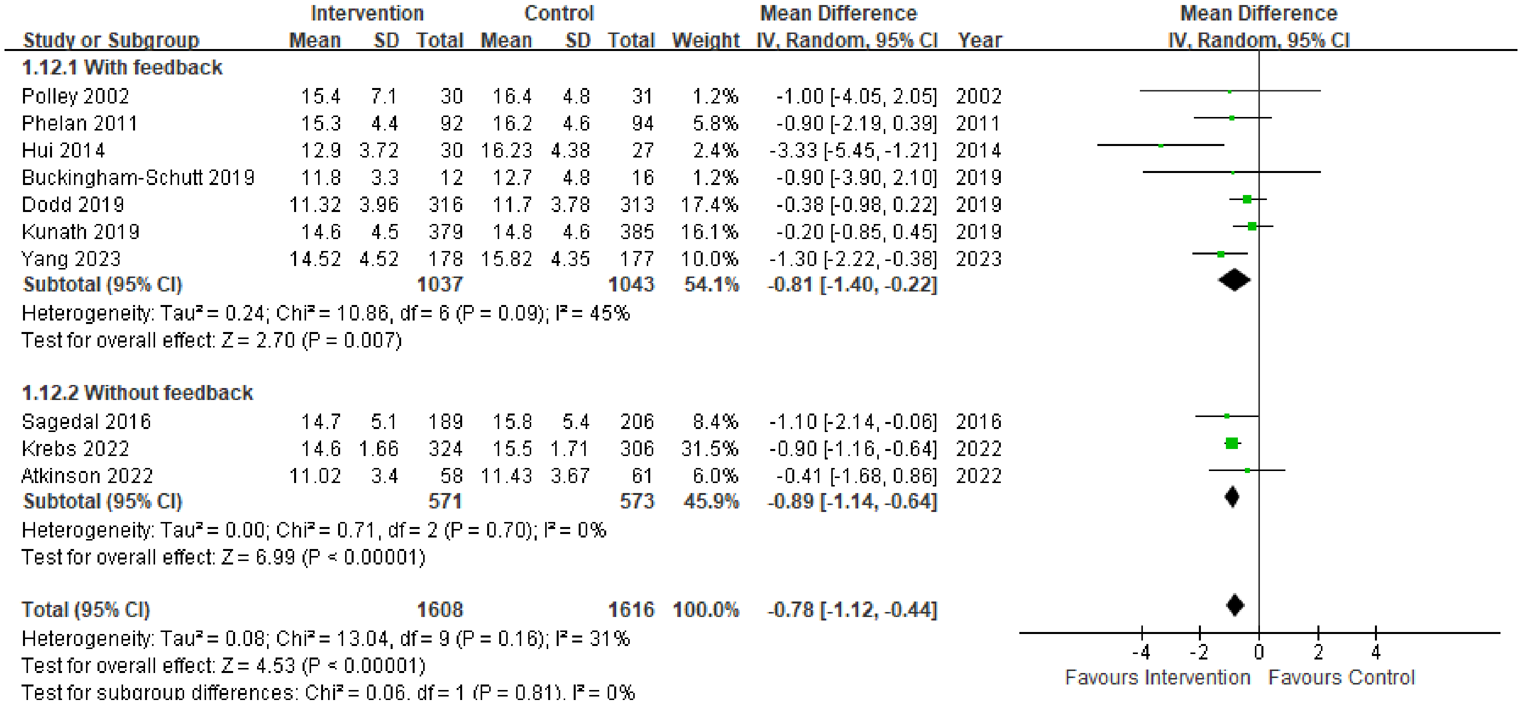

Figure S5. Forest plot of Effect of **Delivery Mode** in the diet component on total GWG

Study or Subgroup	Intervention Mean	Intervention SD	Intervention Total	Control Mean	Control SD	Control Total	Weight	Mean Difference IV, Random, 95% CI	Year
**1.6.1 Face to face**									
Hui 2014	12.9	3.72	30	16.23	4.38	27	2.4%	-3.33 [-5.45, -1.21]	2014
Kunath 2019	14.6	4.5	379	14.8	4.6	385	16.1%	-0.20 [-0.85, 0.45]	2019
Atkinson 2022	11.02	3.4	58	11.43	3.67	61	6.0%	-0.41 [-1.68, 0.86]	2022
Krebs 2022	14.6	1.66	324	15.5	1.71	306	31.5%	-0.90 [-1.16, -0.64]	2022
**Subtotal (95% CI)**			**791**			**779**	**56.0%**	**-0.81 [-1.53, -0.09]**	
Heterogeneity: Tau² = 0.31; Chi² = 9.68, df = 3 (P = 0.02); I² = 69%									
Test for overall effect: Z = 2.20 (P = 0.03)									
**1.6.2 Remote**									
Sagedal 2016	14.7	5.1	189	15.8	5.4	206	8.4%	-1.10 [-2.14, -0.06]	2016
**Subtotal (95% CI)**			**189**			**206**	**8.4%**	**-1.10 [-2.14, -0.06]**	
Heterogeneity: Not applicable									
Test for overall effect: Z = 2.08 (P = 0.04)									
**1.6.3 Face to face and remote**									
Polley 2002	15.4	7.1	30	16.4	4.8	31	1.2%	-1.00 [-4.05, 2.05]	2002
Phelan 2011	15.3	4.4	92	16.2	4.6	94	5.8%	-0.90 [-2.19, 0.39]	2011
Buckingham-Schutt 2019	11.8	3.3	12	12.7	4.8	16	1.2%	-0.90 [-3.90, 2.10]	2019
Dodd 2019	11.32	3.96	316	11.7	3.78	313	17.4%	-0.38 [-0.98, 0.22]	2019
Yang 2023	14.52	4.52	178	15.82	4.35	177	10.0%	-1.30 [-2.22, -0.38]	2023
**Subtotal (95% CI)**			**628**			**631**	**35.6%**	**-0.70 [-1.16, -0.24]**	
Heterogeneity: Tau² = 0.00; Chi² = 2.84, df = 4 (P = 0.58); I² = 0%									
Test for overall effect: Z = 2.99 (P = 0.003)									
**Total (95% CI)**			**1608**			**1616**	**100.0%**	**-0.78 [-1.12, -0.44]**	
Heterogeneity: Tau² = 0.08; Chi² = 13.04, df = 9 (P = 0.16); I² = 31%									
Test for overall effect: Z = 4.53 (P < 0.00001)									
Test for subgroup differences: Chi² = 0.49, df = 2 (P = 0.78), I² = 0%									

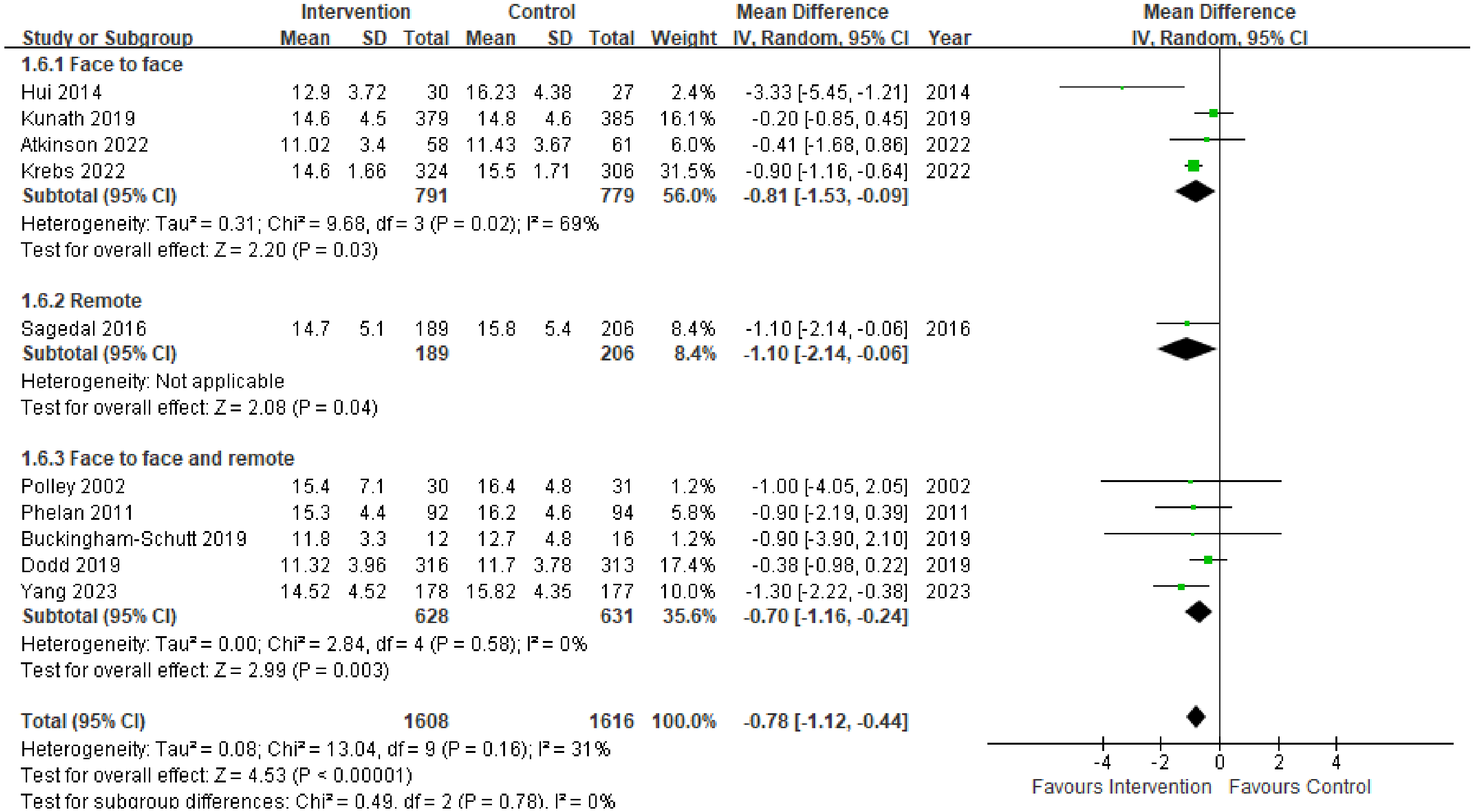

Figure S6. Forest plot of Effect of **Delivery Mode** in the physical activity component on total GWG

	Intervention			Control				Mean Difference	
**Study or Subgroup**	**Mean**	**SD**	**Total**	**Mean**	**SD**	**Total**	**Weight**	**IV, Random, 95% CI**	**Year**
**1.15.1 Face to face**									
Hui 2014	12.9	3.72	30	16.23	4.38	27	2.4%	-3.33 [-5.45, -1.21]	2014
Sagedal 2016	14.7	5.1	189	15.8	5.4	206	8.4%	-1.10 [-2.14, -0.06]	2016
Kunath 2019	14.6	4.5	379	14.8	4.6	385	16.1%	-0.20 [-0.85, 0.45]	2019
Atkinson 2022	11.02	3.4	58	11.43	3.67	61	6.0%	-0.41 [-1.68, 0.86]	2022
Krebs 2022	14.6	1.66	324	15.5	1.71	306	31.5%	-0.90 [-1.16, -0.64]	2022
**Subtotal (95% CI)**			**980**			**985**	**64.4%**	**-0.83 [-1.41, -0.26]**	
Heterogeneity: Tau² = 0.22; Chi² = 9.95, df = 4 (P = 0.04); I² = 60%									
Test for overall effect: Z = 2.83 (P = 0.005)									
**1.15.2 Face to face and remote**									
Polley 2002	15.4	7.1	30	16.4	4.8	31	1.2%	-1.00 [-4.05, 2.05]	2002
Phelan 2011	15.3	4.4	92	16.2	4.6	94	5.8%	-0.90 [-2.19, 0.39]	2011
Buckingham-Schutt 2019	11.8	3.3	12	12.7	4.8	16	1.2%	-0.90 [-3.90, 2.10]	2019
Dodd 2019	11.32	3.96	316	11.7	3.78	313	17.4%	-0.38 [-0.98, 0.22]	2019
Yang 2023	14.52	4.52	178	15.82	4.35	177	10.0%	-1.30 [-2.22, -0.38]	2023
**Subtotal (95% CI)**			**628**			**631**	**35.6%**	**-0.70 [-1.16, -0.24]**	
Heterogeneity: Tau² = 0.00; Chi² = 2.84, df = 4 (P = 0.58); I² = 0%									
Test for overall effect: Z = 2.99 (P = 0.003)									
**Total (95% CI)**			**1608**			**1616**	**100.0%**	**-0.78 [-1.12, -0.44]**	
Heterogeneity: Tau² = 0.08; Chi² = 13.04, df = 9 (P = 0.16); I² = 31%									
Test for overall effect: Z = 4.53 (P < 0.00001)									
Test for subgroup differences: Chi² = 0.13, df = 1 (P = 0.72), I² = 0%									

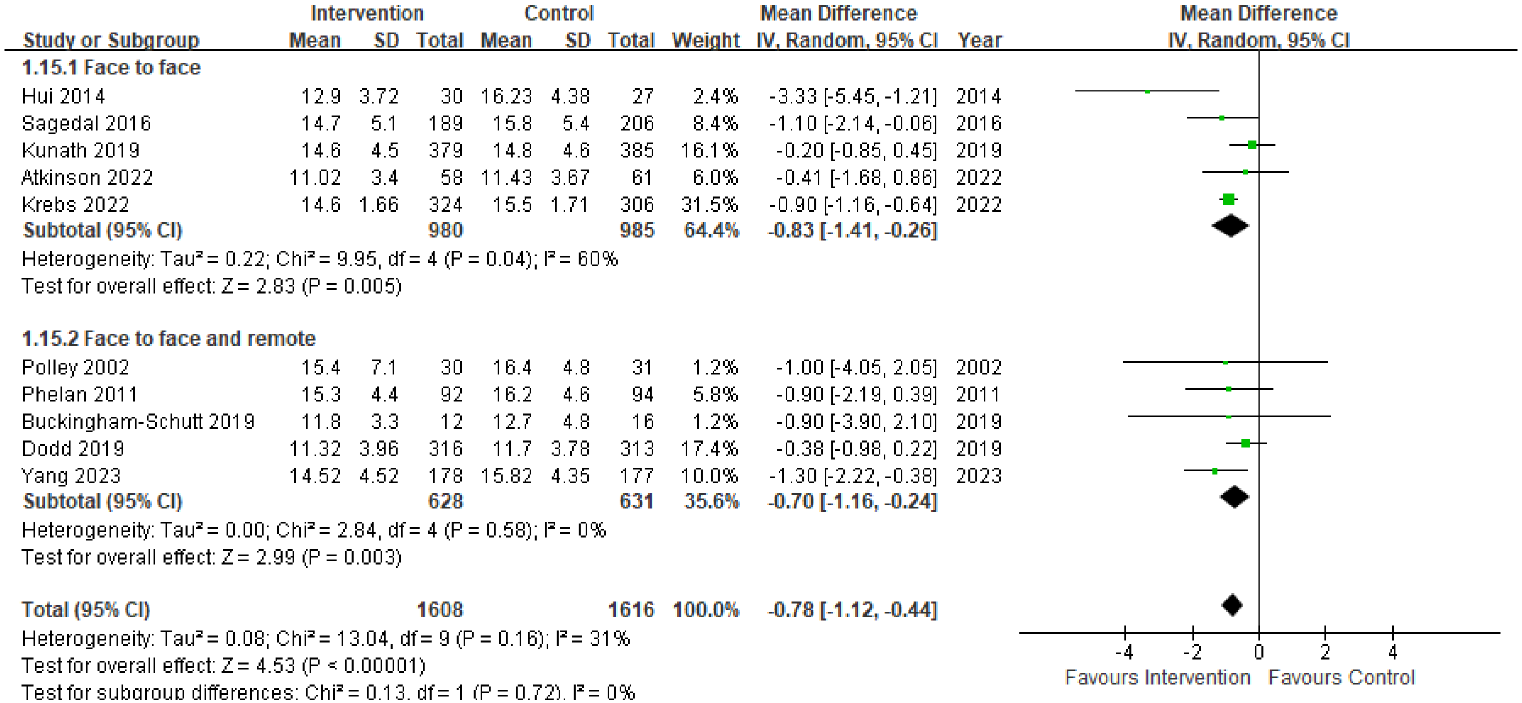

Figure S7. Forest plot of Effect of **Intervention format** in the diet component on total GWG

	Intervention			Control				Mean Difference	
Study or Subgroup	Mean	SD	Total	Mean	SD	Total	Weight	IV, Random, 95% CI	Year
**1.7.1 Individual**									
Polley 2002	15.4	7.1	30	16.4	4.8	31	1.2%	-1.00 [-4.05, 2.05]	2002
Phelan 2011	15.3	4.4	92	16.2	4.6	94	5.8%	-0.90 [-2.19, 0.39]	2011
Hui 2014	12.9	3.72	30	16.23	4.38	27	2.4%	-3.33 [-5.45, -1.21]	2014
Buckingham-Schutt 2019	11.8	3.3	12	12.7	4.8	16	1.2%	-0.90 [-3.90, 2.10]	2019
Kunath 2019	14.6	4.5	379	14.8	4.6	385	16.1%	-0.20 [-0.85, 0.45]	2019
Dodd 2019	11.32	3.96	316	11.7	3.78	313	17.4%	-0.38 [-0.98, 0.22]	2019
Krebs 2022	14.6	1.66	324	15.5	1.71	306	31.5%	-0.90 [-1.16, -0.64]	2022
Atkinson 2022	11.02	3.4	58	11.43	3.67	61	6.0%	-0.41 [-1.68, 0.86]	2022
Yang 2023	14.52	4.52	178	15.82	4.35	177	10.0%	-1.30 [-2.22, -0.38]	2023
**Subtotal (95% CI)**			**1419**			**1410**	**91.6%**	**-0.76 [-1.14, -0.38]**	
Heterogeneity: Tau² = 0.10; Chi² = 12.72, df = 8 (P = 0.12); I² = 37%									
Test for overall effect: Z = 3.95 (P < 0.0001)									
**1.7.2 Individual and group**									
Sagedal 2016	14.7	5.1	189	15.8	5.4	206	8.4%	-1.10 [-2.14, -0.06]	2016
**Subtotal (95% CI)**			**189**			**206**	**8.4%**	**-1.10 [-2.14, -0.06]**	
Heterogeneity: Not applicable									
Test for overall effect: Z = 2.08 (P = 0.04)									
**Total (95% CI)**			**1608**			**1616**	**100.0%**	**-0.78 [-1.12, -0.44]**	
Heterogeneity: Tau² = 0.08; Chi² = 13.04, df = 9 (P = 0.16); I² = 31%									
Test for overall effect: Z = 4.53 (P < 0.00001)									
Test for subgroup differences: Chi² = 0.37, df = 1 (P = 0.54), I² = 0%									

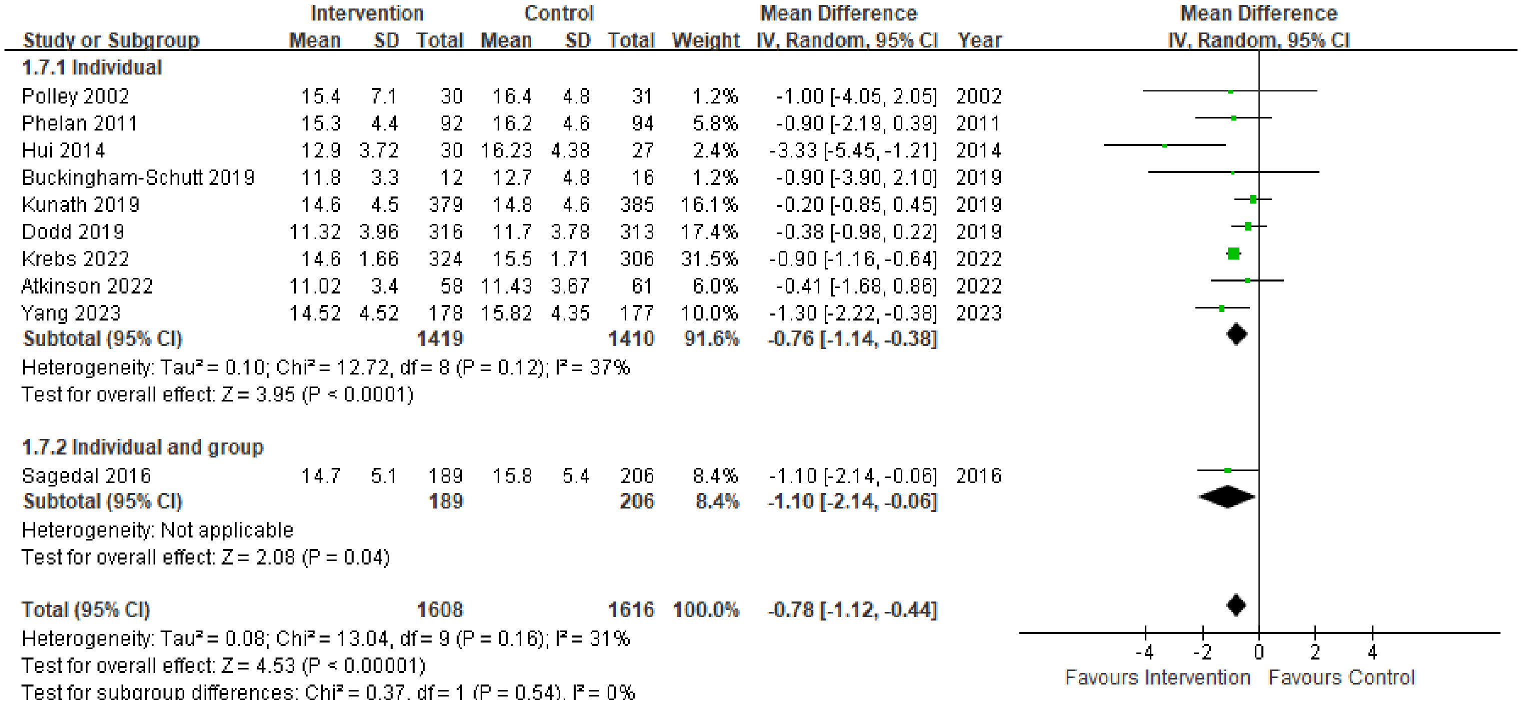

Figure S8. Forest plot of Effect of **Intervention format** in the physical activity component on total GWG

Study or Subgroup	Intervention Mean	Intervention SD	Intervention Total	Control Mean	Control SD	Control Total	Weight	Mean Difference IV, Random, 95% CI	Year
**1.16.1 Individual**									
Polley 2002	15.4	7.1	30	16.4	4.8	31	1.2%	-1.00 [-4.05, 2.05]	2002
Phelan 2011	15.3	4.4	92	16.2	4.6	94	5.8%	-0.90 [-2.19, 0.39]	2011
Dodd 2019	11.32	3.96	316	11.7	3.78	313	17.4%	-0.38 [-0.98, 0.22]	2019
Buckingham-Schutt 2019	11.8	3.3	12	12.7	4.8	16	1.2%	-0.90 [-3.90, 2.10]	2019
Kunath 2019	14.6	4.5	379	14.8	4.6	385	16.1%	-0.20 [-0.85, 0.45]	2019
Krebs 2022	14.6	1.66	324	15.5	1.71	306	31.5%	-0.90 [-1.16, -0.64]	2022
Atkinson 2022	11.02	3.4	58	11.43	3.67	61	6.0%	-0.41 [-1.68, 0.86]	2022
Yang 2023	14.52	4.52	178	15.82	4.35	177	10.0%	-1.30 [-2.22, -0.38]	2023
**Subtotal (95% CI)**			**1389**			**1383**	**89.2%**	**-0.76 [-0.98, -0.53]**	
Heterogeneity: Tau² = 0.00; Chi² = 7.17, df = 7 (P = 0.41); I² = 2%									
Test for overall effect: Z = 6.53 (P < 0.00001)									
**1.16.2 Individual and group**									
Hui 2014	12.9	3.72	30	16.23	4.38	27	2.4%	-3.33 [-5.45, -1.21]	2014
Sagedal 2016	14.7	5.1	189	15.8	5.4	206	8.4%	-1.10 [-2.14, -0.06]	2016
**Subtotal (95% CI)**			**219**			**233**	**10.8%**	**-2.01 [-4.16, 0.14]**	
Heterogeneity: Tau² = 1.76; Chi² = 3.43, df = 1 (P = 0.06); I² = 71%									
Test for overall effect: Z = 1.84 (P = 0.07)									
**Total (95% CI)**			**1608**			**1616**	**100.0%**	**-0.78 [-1.12, -0.44]**	
Heterogeneity: Tau² = 0.08; Chi² = 13.04, df = 9 (P = 0.16); I² = 31%									
Test for overall effect: Z = 4.53 (P < 0.00001)									
Test for subgroup differences: Chi² = 1.30, df = 1 (P = 0.25), I² = 23.3%									

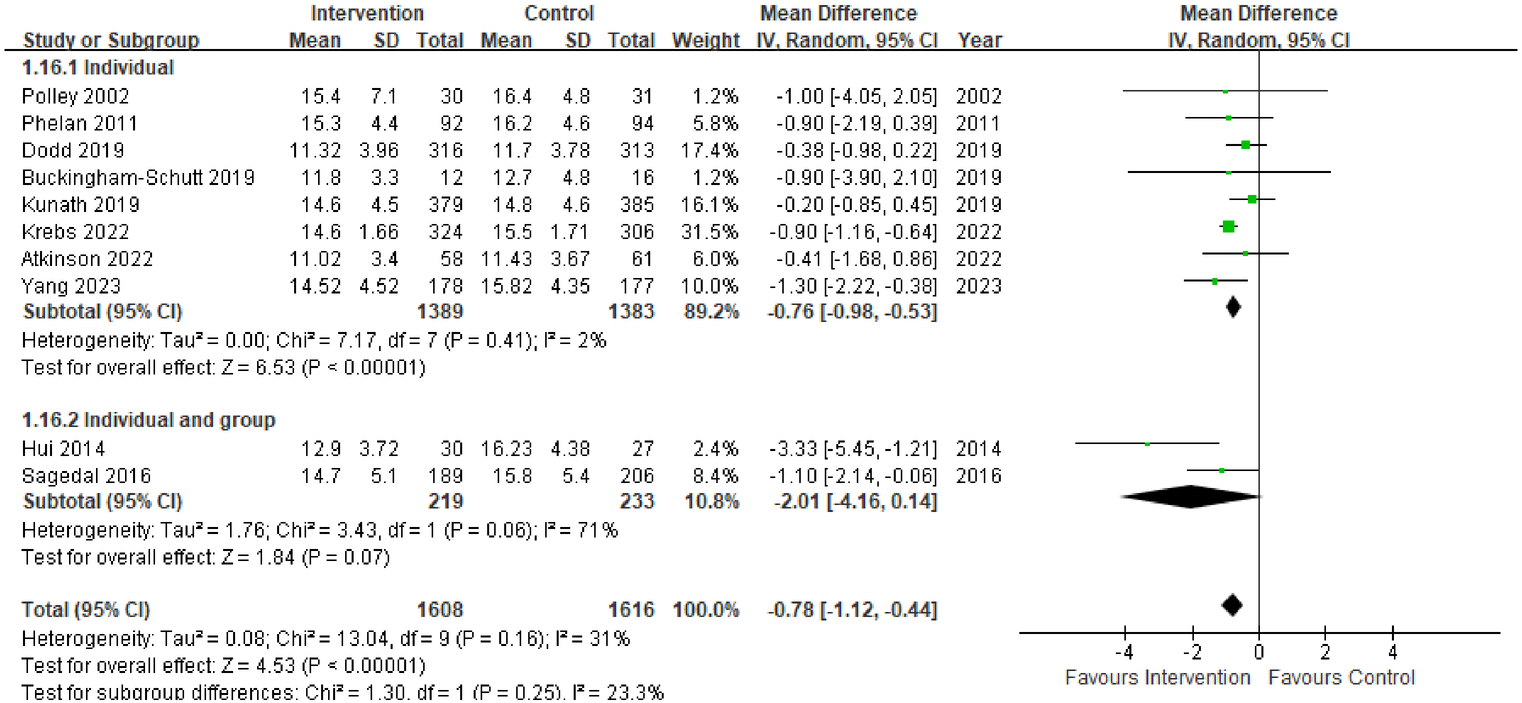

Figure S9. Forest plot of Effect of **Provider** in the diet component on total GWG

Study or Subgroup	Intervention Mean	Intervention SD	Intervention Total	Control Mean	Control SD	Control Total	Weight	Mean Difference IV, Random, 95% CI	Year
**1.8.1 Nutritionist**									
Phelan 2011	15.3	4.4	92	16.2	4.6	94	5.8%	-0.90 [-2.19, 0.39]	2011
Hui 2014	12.9	3.72	30	16.23	4.38	27	2.4%	-3.33 [-5.45, -1.21]	2014
Dodd 2019	11.32	3.96	316	11.7	3.78	313	17.4%	-0.38 [-0.98, 0.22]	2019
Buckingham-Schutt 2019	11.8	3.3	12	12.7	4.8	16	1.2%	-0.90 [-3.90, 2.10]	2019
Atkinson 2022	11.02	3.4	58	11.43	3.67	61	6.0%	-0.41 [-1.68, 0.86]	2022
**Subtotal (95% CI)**			**508**			**511**	**32.8%**	**-0.87 [-1.68, -0.05]**	
Heterogeneity: Tau² = 0.35; Chi² = 7.19, df = 4 (P = 0.13); I² = 44%									
Test for overall effect: Z = 2.09 (P = 0.04)									
**1.8.2 Other**									
Polley 2002	15.4	7.1	30	16.4	4.8	31	1.2%	-1.00 [-4.05, 2.05]	2002
Sagedal 2016	14.7	5.1	189	15.8	5.4	206	8.4%	-1.10 [-2.14, -0.06]	2016
Kunath 2019	14.6	4.5	379	14.8	4.6	385	16.1%	-0.20 [-0.85, 0.45]	2019
Krebs 2022	14.6	1.66	324	15.5	1.71	306	31.5%	-0.90 [-1.16, -0.64]	2022
Yang 2023	14.52	4.52	178	15.82	4.35	177	10.0%	-1.30 [-2.22, -0.38]	2023
**Subtotal (95% CI)**			**1100**			**1105**	**67.2%**	**-0.82 [-1.17, -0.47]**	
Heterogeneity: Tau² = 0.04; Chi² = 5.18, df = 4 (P = 0.27); I² = 23%									
Test for overall effect: Z = 4.59 (P < 0.00001)									
**Total (95% CI)**			**1608**			**1616**	**100.0%**	**-0.78 [-1.12, -0.44]**	
Heterogeneity: Tau² = 0.08; Chi² = 13.04, df = 9 (P = 0.16); I² = 31%									
Test for overall effect: Z = 4.53 (P < 0.00001)									
Test for subgroup differences: Chi² = 0.01, df = 1 (P = 0.92), I² = 0%									

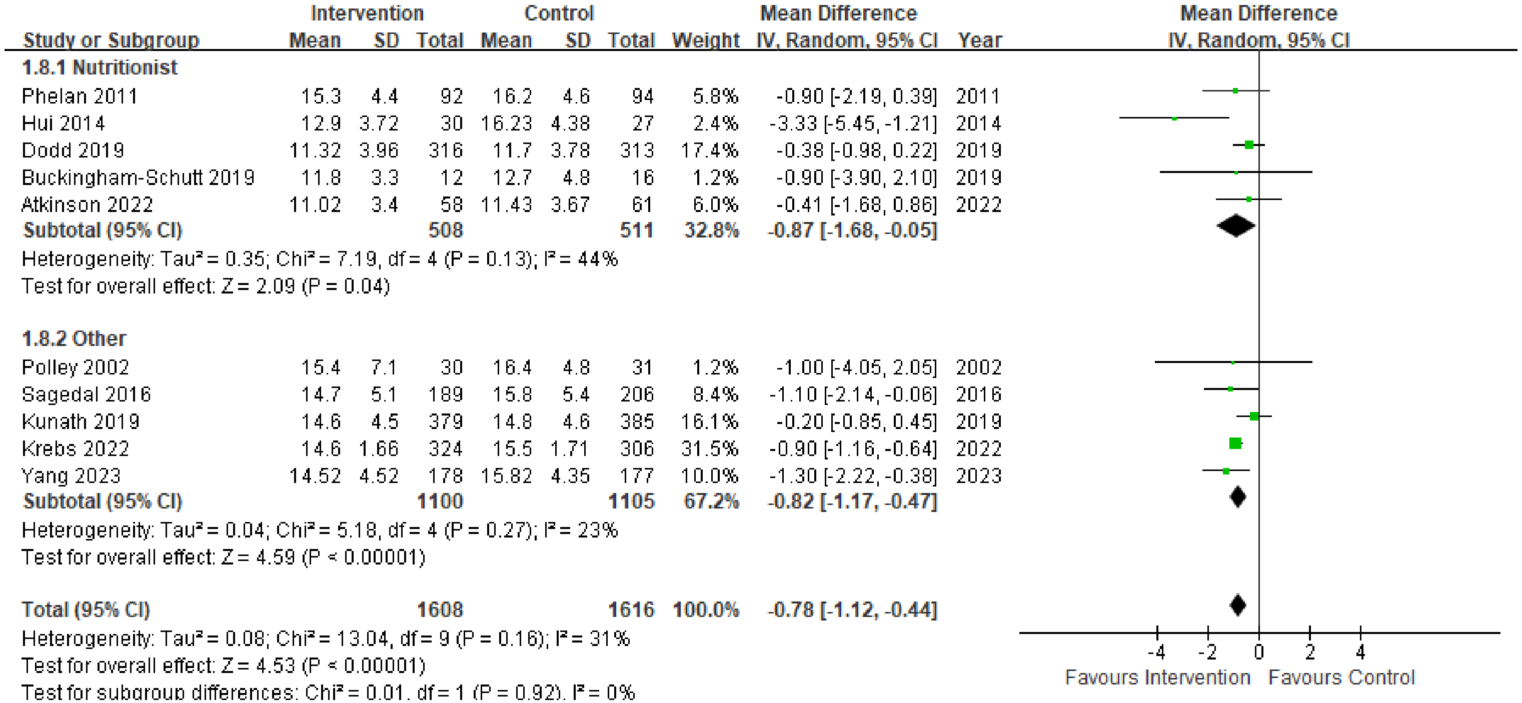

Figure S10. Forest plot of Effect of **Provider** in the physical activity component on total GWG

Study or Subgroup	Intervention Mean	Intervention SD	Intervention Total	Control Mean	Control SD	Control Total	Weight	Mean Difference IV, Random, 95% CI	Year
**1.17.1 Sports health professionals**									
Hui 2014	12.9	3.72	30	16.23	4.38	27	2.4%	-3.33 [-5.45, -1.21]	2014
**Subtotal (95% CI)**			**30**			**27**	**2.4%**	**-3.33 [-5.45, -1.21]**	
Heterogeneity: Not applicable									
Test for overall effect: Z = 3.08 (P = 0.002)									
**1.17.2 Other**									
Polley 2002	15.4	7.1	30	16.4	4.8	31	1.2%	-1.00 [-4.05, 2.05]	2002
Phelan 2011	15.3	4.4	92	16.2	4.6	94	5.8%	-0.90 [-2.19, 0.39]	2011
Sagedal 2016	14.7	5.1	189	15.8	5.4	206	8.4%	-1.10 [-2.14, -0.06]	2016
Dodd 2019	11.32	3.96	316	11.7	3.78	313	17.4%	-0.38 [-0.98, 0.22]	2019
Buckingham-Schutt 2019	11.8	3.3	12	12.7	4.8	16	1.2%	-0.90 [-3.90, 2.10]	2019
Kunath 2019	14.6	4.5	379	14.8	4.6	385	16.1%	-0.20 [-0.85, 0.45]	2019
Atkinson 2022	11.02	3.4	58	11.43	3.67	61	6.0%	-0.41 [-1.68, 0.86]	2022
Krebs 2022	14.6	1.66	324	15.5	1.71	306	31.5%	-0.90 [-1.16, -0.64]	2022
Yang 2023	14.52	4.52	178	15.82	4.35	177	10.0%	-1.30 [-2.22, -0.38]	2023
**Subtotal (95% CI)**			**1578**			**1589**	**97.6%**	**-0.78 [-0.99, -0.57]**	
Heterogeneity: Tau² = 0.00; Chi² = 7.55, df = 8 (P = 0.48); I² = 0%									
Test for overall effect: Z = 7.36 (P < 0.00001)									
**Total (95% CI)**			**1608**			**1616**	**100.0%**	**-0.78 [-1.12, -0.44]**	
Heterogeneity: Tau² = 0.08; Chi² = 13.04, df = 9 (P = 0.16); I² = 31%									
Test for overall effect: Z = 4.53 (P < 0.00001)									
Test for subgroup differences: Chi² = 5.49, df = 1 (P = 0.02), I² = 81.8%									

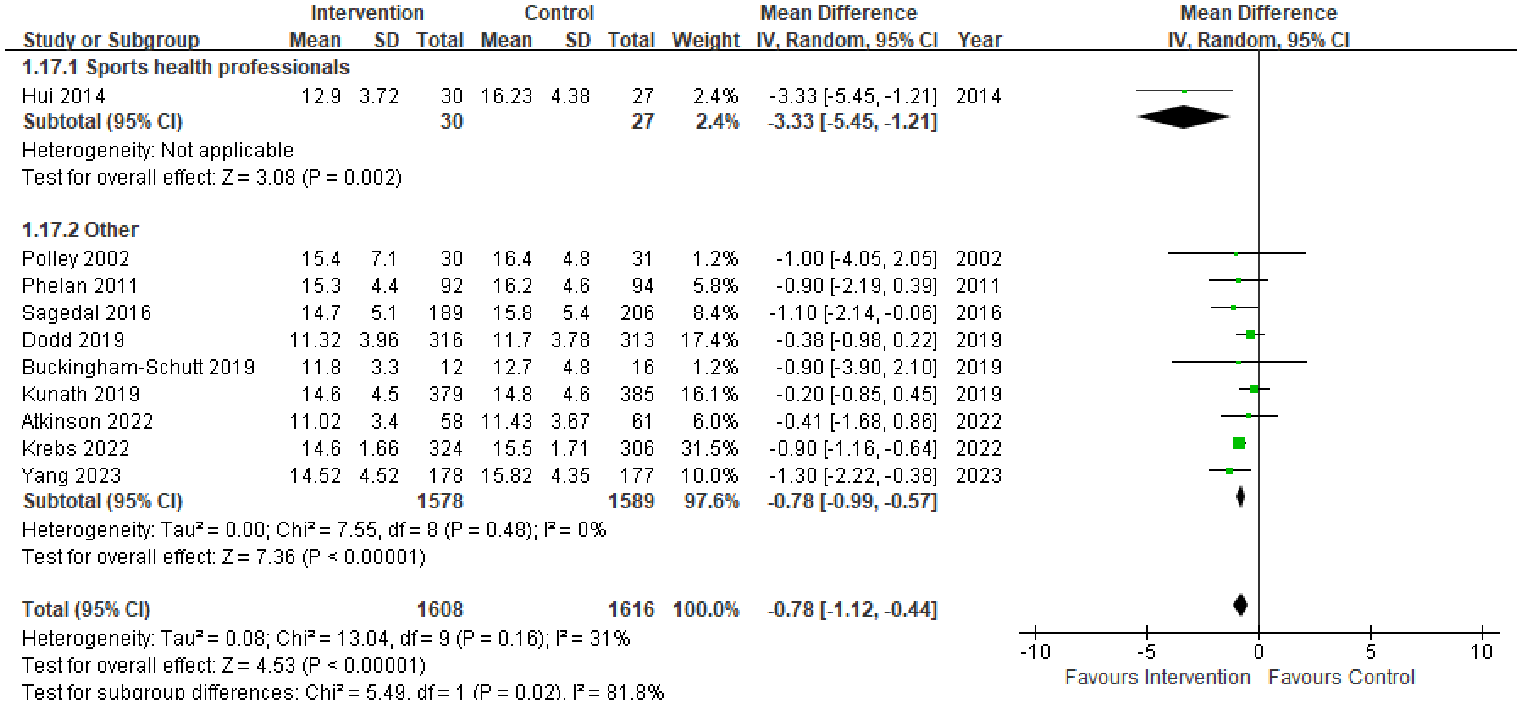

Figure S11. Forest plot of Effect of **Location** in the diet component on total GWG

Study or Subgroup	Intervention Mean	Intervention SD	Intervention Total	Control Mean	Control SD	Control Total	Weight	Mean Difference IV, Random, 95% CI	Year
**1.11.1 Healthcare Facility**									
Polley 2002	15.4	7.1	30	16.4	4.8	31	1.2%	-1.00 [-4.05, 2.05]	2002
Phelan 2011	15.3	4.4	92	16.2	4.6	94	5.8%	-0.90 [-2.19, 0.39]	2011
Kunath 2019	14.6	4.5	379	14.8	4.6	385	16.1%	-0.20 [-0.85, 0.45]	2019
Dodd 2019	11.32	3.96	316	11.7	3.78	313	17.4%	-0.38 [-0.98, 0.22]	2019
Buckingham-Schutt 2019	11.8	3.3	12	12.7	4.8	16	1.2%	-0.90 [-3.90, 2.10]	2019
Krebs 2022	14.6	1.66	324	15.5	1.71	306	31.5%	-0.90 [-1.16, -0.64]	2022
Atkinson 2022	11.02	3.4	58	11.43	3.67	61	6.0%	-0.41 [-1.68, 0.86]	2022
Yang 2023	14.52	4.52	178	15.82	4.35	177	10.0%	-1.30 [-2.22, -0.38]	2023
**Subtotal (95% CI)**			**1389**			**1383**	**89.2%**	**-0.76 [-0.98, -0.53]**	
Heterogeneity: Tau² = 0.00; Chi² = 7.17, df = 7 (P = 0.41); I² = 2%									
Test for overall effect: Z = 6.53 (P < 0.00001)									
**1.11.2 Other**									
Hui 2014	12.9	3.72	30	16.23	4.38	27	2.4%	-3.33 [-5.45, -1.21]	2014
Sagedal 2016	14.7	5.1	189	15.8	5.4	206	8.4%	-1.10 [-2.14, -0.06]	2016
**Subtotal (95% CI)**			**219**			**233**	**10.8%**	**-2.01 [-4.16, 0.14]**	
Heterogeneity: Tau² = 1.76; Chi² = 3.43, df = 1 (P = 0.06); I² = 71%									
Test for overall effect: Z = 1.84 (P = 0.07)									
**Total (95% CI)**			**1608**			**1616**	**100.0%**	**-0.78 [-1.12, -0.44]**	
Heterogeneity: Tau² = 0.08; Chi² = 13.04, df = 9 (P = 0.16); I² = 31%									
Test for overall effect: Z = 4.53 (P < 0.00001)									
Test for subgroup differences: Chi² = 1.30, df = 1 (P = 0.25), I² = 23.3%									

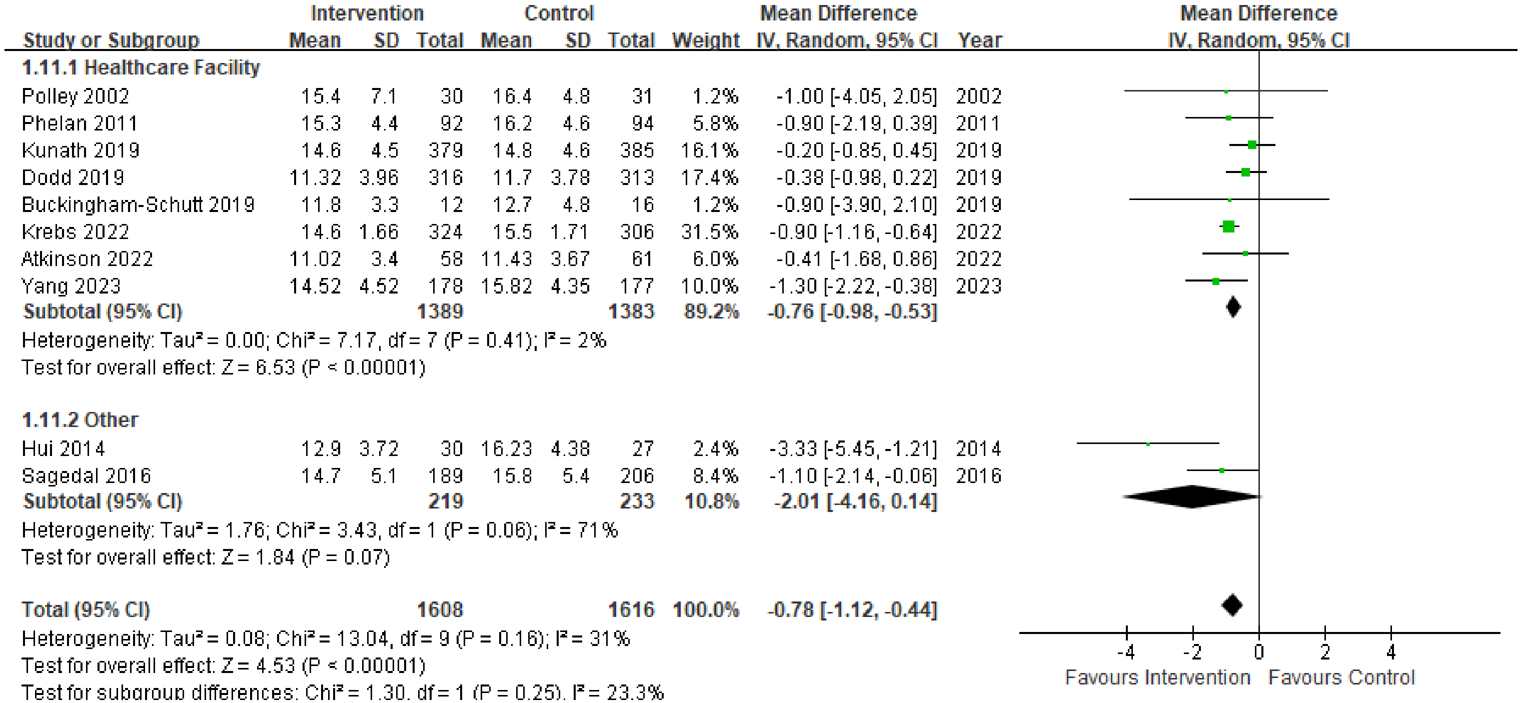

Figure S12. Forest plot of Effect of **Location** in the physical activity component on total GWG

Study or Subgroup	Intervention Mean	Intervention SD	Intervention Total	Control Mean	Control SD	Control Total	Weight	Mean Difference IV, Random, 95% CI	Year
**1.19.1 Healthcare Facility**									
Polley 2002	15.4	7.1	30	16.4	4.8	31	1.2%	-1.00 [-4.05, 2.05]	2002
Phelan 2011	15.3	4.4	92	16.2	4.6	94	5.8%	-0.90 [-2.19, 0.39]	2011
Buckingham-Schutt 2019	11.8	3.3	12	12.7	4.8	16	1.2%	-0.90 [-3.90, 2.10]	2019
Dodd 2019	11.32	3.96	316	11.7	3.78	313	17.4%	-0.38 [-0.98, 0.22]	2019
Kunath 2019	14.6	4.5	379	14.8	4.6	385	16.1%	-0.20 [-0.85, 0.45]	2019
Atkinson 2022	11.02	3.4	58	11.43	3.67	61	6.0%	-0.41 [-1.68, 0.86]	2022
Krebs 2022	14.6	1.66	324	15.5	1.71	306	31.5%	-0.90 [-1.16, -0.64]	2022
Yang 2023	14.52	4.52	178	15.82	4.35	177	10.0%	-1.30 [-2.22, -0.38]	2023
**Subtotal (95% CI)**			**1389**			**1383**	**89.2%**	**-0.76 [-0.98, -0.53]**	
Heterogeneity: Tau² = 0.00; Chi² = 7.17, df = 7 (P = 0.41); I² = 2%									
Test for overall effect: Z = 6.53 (P < 0.00001)									
**1.19.2 Other**									
Hui 2014	12.9	3.72	30	16.23	4.38	27	2.4%	-3.33 [-5.45, -1.21]	2014
Sagedal 2016	14.7	5.1	189	15.8	5.4	206	8.4%	-1.10 [-2.14, -0.06]	2016
**Subtotal (95% CI)**			**219**			**233**	**10.8%**	**-2.01 [-4.16, 0.14]**	
Heterogeneity: Tau² = 1.76; Chi² = 3.43, df = 1 (P = 0.06); I² = 71%									
Test for overall effect: Z = 1.84 (P = 0.07)									
**Total (95% CI)**			**1608**			**1616**	**100.0%**	**-0.78 [-1.12, -0.44]**	
Heterogeneity: Tau² = 0.08; Chi² = 13.04, df = 9 (P = 0.16); I² = 31%									
Test for overall effect: Z = 4.53 (P < 0.00001)									
Test for subgroup differences: Chi² = 1.30, df = 1 (P = 0.25), I² = 23.3%									

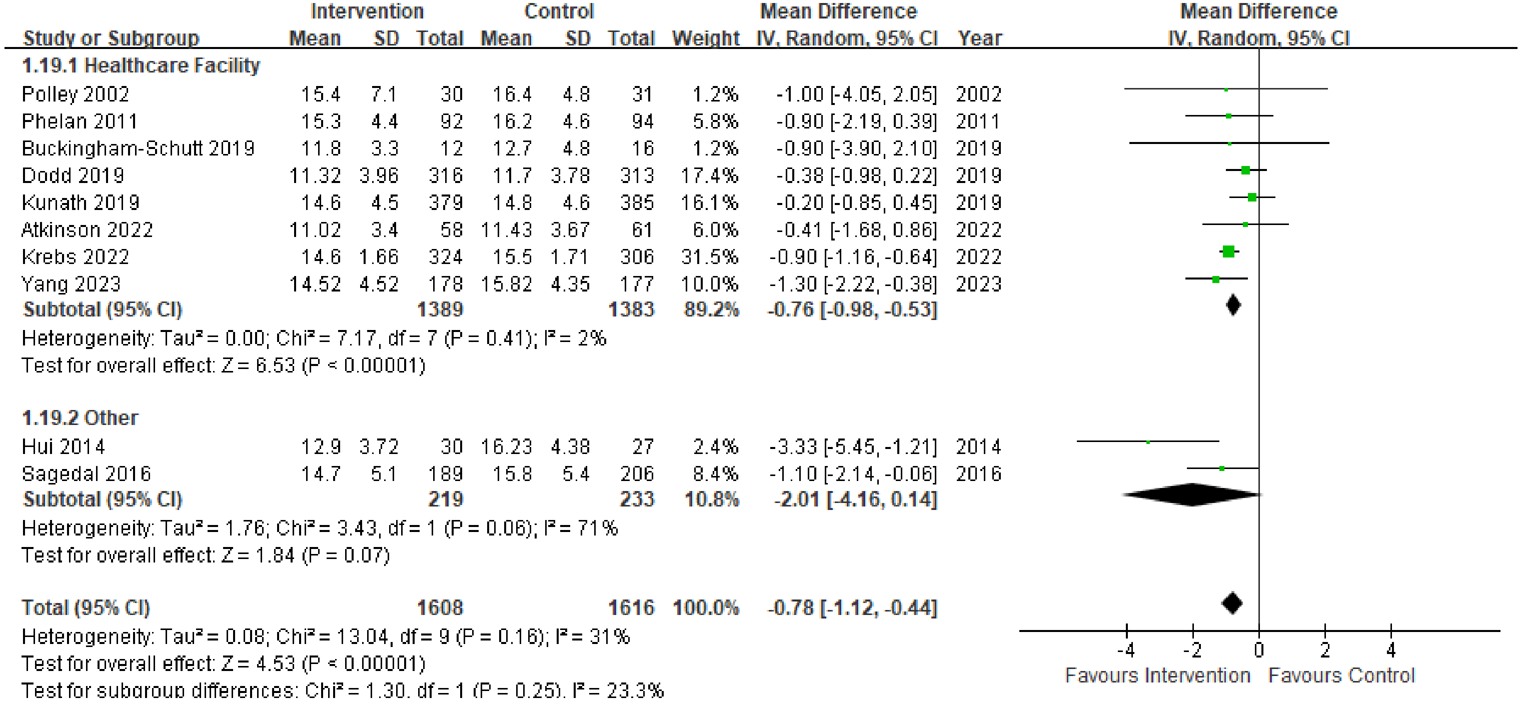

Figure S13. Forest plot of Effect of **Duration** in the diet component on total GWG

Study or Subgroup	Intervention Mean	Intervention SD	Intervention Total	Control Mean	Control SD	Control Total	Weight	Mean Difference IV, Random, 95% CI	Year
**1.10.1 Low: 1-12**									
Hui 2014	12.9	3.72	30	16.23	4.38	27	2.4%	-3.33 [-5.45, -1.21]	2014
**Subtotal (95% CI)**			**30**			**27**	**2.4%**	**-3.33 [-5.45, -1.21]**	
Heterogeneity: Not applicable									
Test for overall effect: Z = 3.08 (P = 0.002)									
**1.10.2 Moderate: 13-20**									
Polley 2002	15.4	7.1	30	16.4	4.8	31	1.2%	-1.00 [-4.05, 2.05]	2002
Sagedal 2016	14.7	5.1	189	15.8	5.4	206	8.4%	-1.10 [-2.14, -0.06]	2016
Dodd 2019	11.32	3.96	316	11.7	3.78	313	17.4%	-0.38 [-0.98, 0.22]	2019
**Subtotal (95% CI)**			**535**			**550**	**26.9%**	**-0.58 [-1.09, -0.06]**	
Heterogeneity: Tau² = 0.00; Chi² = 1.46, df = 2 (P = 0.48); I² = 0%									
Test for overall effect: Z = 2.19 (P = 0.03)									
**1.10.3 High: ⩾21**									
Phelan 2011	15.3	4.4	92	16.2	4.6	94	5.8%	-0.90 [-2.19, 0.39]	2011
Buckingham-Schutt 2019	11.8	3.3	12	12.7	4.8	16	1.2%	-0.90 [-3.90, 2.10]	2019
Kunath 2019	14.6	4.5	379	14.8	4.6	385	16.1%	-0.20 [-0.85, 0.45]	2019
Atkinson 2022	11.02	3.4	58	11.43	3.67	61	6.0%	-0.41 [-1.68, 0.86]	2022
Krebs 2022	14.6	1.66	324	15.5	1.71	306	31.5%	-0.90 [-1.16, -0.64]	2022
Yang 2023	14.52	4.52	178	15.82	4.35	177	10.0%	-1.30 [-2.22, -0.38]	2023
**Subtotal (95% CI)**			**1043**			**1039**	**70.7%**	**-0.80 [-1.07, -0.52]**	
Heterogeneity: Tau² = 0.01; Chi² = 5.36, df = 5 (P = 0.37); I² = 7%									
Test for overall effect: Z = 5.73 (P < 0.00001)									
**Total (95% CI)**			**1608**			**1616**	**100.0%**	**-0.78 [-1.12, -0.44]**	
Heterogeneity: Tau² = 0.08; Chi² = 13.04, df = 9 (P = 0.16); I² = 31%									
Test for overall effect: Z = 4.53 (P < 0.00001)									
Test for subgroup differences: Chi² = 6.17, df = 2 (P = 0.05), I² = 67.6%									

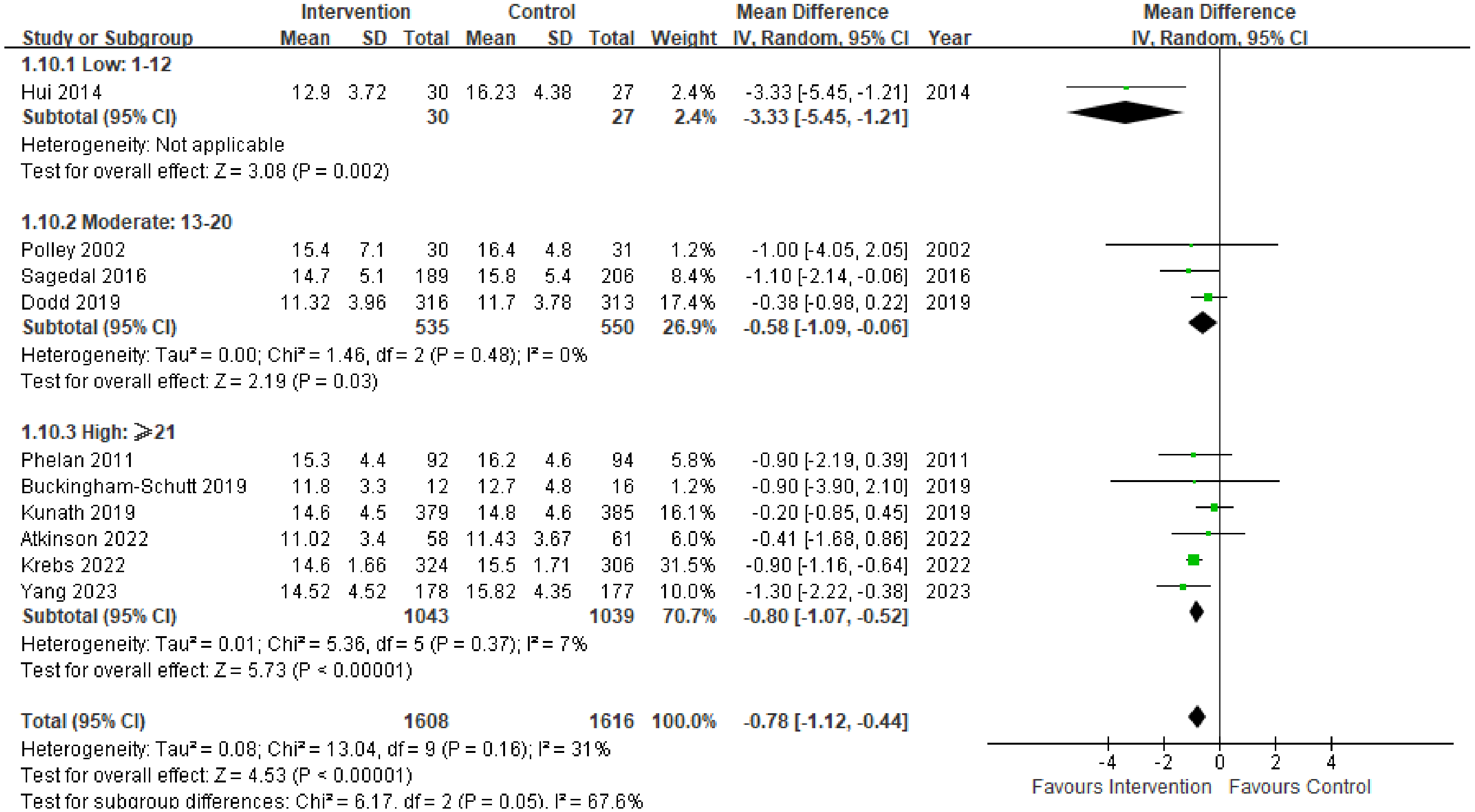

Figure S14. Forest plot of Effect of **Duration** in the physical activity component on total GWG

Study or Subgroup	Intervention Mean	Intervention SD	Intervention Total	Control Mean	Control SD	Control Total	Weight	Mean Difference IV, Random, 95% CI	Year
**1.20.1 Moderate: 13-20**									
Polley 2002	15.4	7.1	30	16.4	4.8	31	1.2%	-1.00 [-4.05, 2.05]	2002
Sagedal 2016	14.7	5.1	189	15.8	5.4	206	8.4%	-1.10 [-2.14, -0.06]	2016
Dodd 2019	11.32	3.96	316	11.7	3.78	313	17.4%	-0.38 [-0.98, 0.22]	2019
**Subtotal (95% CI)**			**535**			**550**	**26.9%**	**-0.58 [-1.09, -0.06]**	
Heterogeneity: Tau² = 0.00; Chi² = 1.46, df = 2 (P = 0.48); I² = 0%									
Test for overall effect: Z = 2.19 (P = 0.03)									
**1.20.2 High: ⩾21**									
Phelan 2011	15.3	4.4	92	16.2	4.6	94	5.8%	-0.90 [-2.19, 0.39]	2011
Hui 2014	12.9	3.72	30	16.23	4.38	27	2.4%	-3.33 [-5.45, -1.21]	2014
Buckingham-Schutt 2019	11.8	3.3	12	12.7	4.8	16	1.2%	-0.90 [-3.90, 2.10]	2019
Kunath 2019	14.6	4.5	379	14.8	4.6	385	16.1%	-0.20 [-0.85, 0.45]	2019
Atkinson 2022	11.02	3.4	58	11.43	3.67	61	6.0%	-0.41 [-1.68, 0.86]	2022
Krebs 2022	14.6	1.66	324	15.5	1.71	306	31.5%	-0.90 [-1.16, -0.64]	2022
Yang 2023	14.52	4.52	178	15.82	4.35	177	10.0%	-1.30 [-2.22, -0.38]	2023
**Subtotal (95% CI)**			**1073**			**1066**	**73.1%**	**-0.86 [-1.33, -0.39]**	
Heterogeneity: Tau² = 0.14; Chi² = 10.67, df = 6 (P = 0.10); I² = 44%									
Test for overall effect: Z = 3.61 (P = 0.0003)									
**Total (95% CI)**			**1608**			**1616**	**100.0%**	**-0.78 [-1.12, -0.44]**	
Heterogeneity: Tau² = 0.08; Chi² = 13.04, df = 9 (P = 0.16); I² = 31%									
Test for overall effect: Z = 4.53 (P < 0.00001)									
Test for subgroup differences: Chi² = 0.64, df = 1 (P = 0.42), I² = 0%									

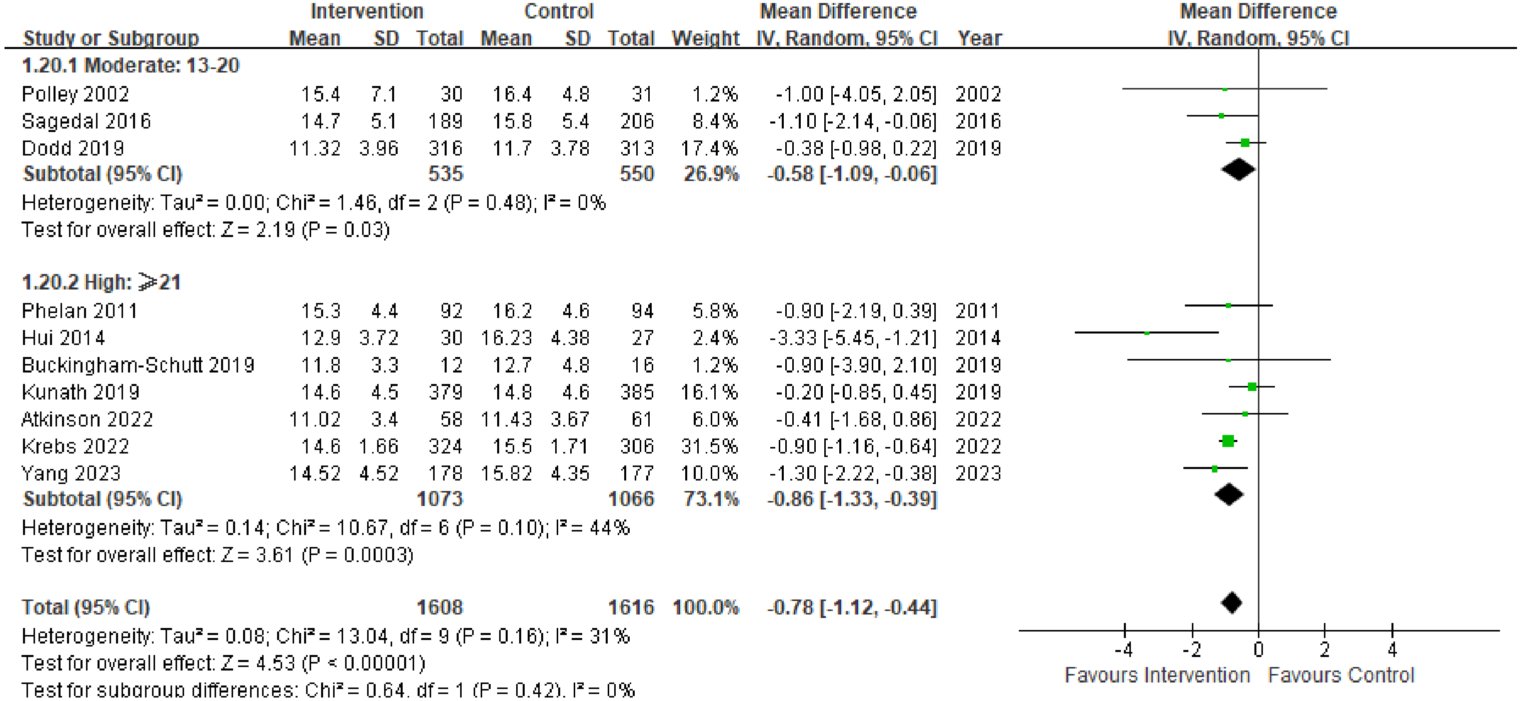

Figure S15. Forest plot of Effect of **No. interventions** in the diet component on total GWG

	Intervention			Control				Mean Difference	
Study or Subgroup	Mean	SD	Total	Mean	SD	Total	Weight	IV, Random, 95% CI	Year
**1.9.1 Low: 1-5**									
Hui 2014	12.9	3.72	30	16.23	4.38	27	2.4%	-3.33 [-5.45, -1.21]	2014
Sagedal 2016	14.7	5.1	189	15.8	5.4	206	8.4%	-1.10 [-2.14, -0.06]	2016
Kunath 2019	14.6	4.5	379	14.8	4.6	385	16.1%	-0.20 [-0.85, 0.45]	2019
Yang 2023	14.52	4.52	178	15.82	4.35	177	10.0%	-1.30 [-2.22, -0.38]	2023
**Subtotal (95% CI)**			**776**			**795**	**36.9%**	**-1.16 [-2.11, -0.20]**	
Heterogeneity: Tau² = 0.62; Chi² = 10.23, df = 3 (P = 0.02); I² = 71%									
Test for overall effect: Z = 2.38 (P = 0.02)									
**1.9.2 Moderate: 6-20**									
Polley 2002	15.4	7.1	30	16.4	4.8	31	1.2%	-1.00 [-4.05, 2.05]	2002
Phelan 2011	15.3	4.4	92	16.2	4.6	94	5.8%	-0.90 [-2.19, 0.39]	2011
Buckingham-Schutt 2019	11.8	3.3	12	12.7	4.8	16	1.2%	-0.90 [-3.90, 2.10]	2019
Dodd 2019	11.32	3.96	316	11.7	3.78	313	17.4%	-0.38 [-0.98, 0.22]	2019
Atkinson 2022	11.02	3.4	58	11.43	3.67	61	6.0%	-0.41 [-1.68, 0.86]	2022
Krebs 2022	14.6	1.66	324	15.5	1.71	306	31.5%	-0.90 [-1.16, -0.64]	2022
**Subtotal (95% CI)**			**832**			**821**	**63.1%**	**-0.81 [-1.04, -0.58]**	
Heterogeneity: Tau² = 0.00; Chi² = 2.81, df = 5 (P = 0.73); I² = 0%									
Test for overall effect: Z = 6.82 (P < 0.00001)									
**Total (95% CI)**			**1608**			**1616**	**100.0%**	**-0.78 [-1.12, -0.44]**	
Heterogeneity: Tau² = 0.08; Chi² = 13.04, df = 9 (P = 0.16); I² = 31%									
Test for overall effect: Z = 4.53 (P < 0.00001)									
Test for subgroup differences: Chi² = 0.49, df = 1 (P = 0.48), I² = 0%									

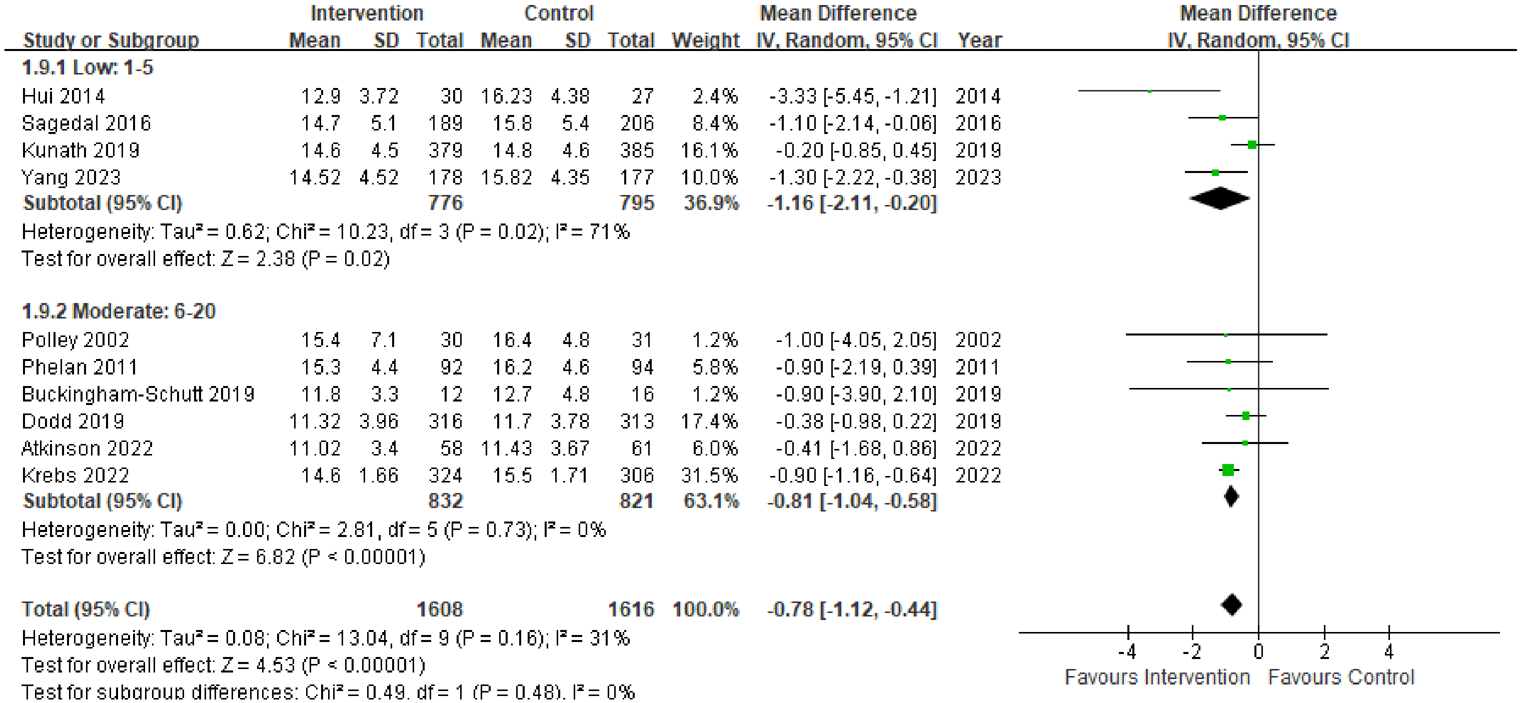

Figure S16. Forest plot of Effect of **No. interventions** in the physical activity component on total GWG

Study or Subgroup	Intervention Mean	Intervention SD	Intervention Total	Control Mean	Control SD	Control Total	Weight	Mean Difference IV, Random, 95% CI	Year
**1.18.1 Moderate: 6-20**									
Polley 2002	15.4	7.1	30	16.4	4.8	31	1.2%	-1.00 [-4.05, 2.05]	2002
Phelan 2011	15.3	4.4	92	16.2	4.6	94	5.8%	-0.90 [-2.19, 0.39]	2011
Dodd 2019	11.32	3.96	316	11.7	3.78	313	17.4%	-0.38 [-0.98, 0.22]	2019
Krebs 2022	14.6	1.66	324	15.5	1.71	306	31.5%	-0.90 [-1.16, -0.64]	2022
**Subtotal (95% CI)**			**762**			**744**	**55.9%**	**-0.82 [-1.06, -0.58]**	
Heterogeneity: Tau² = 0.00; Chi² = 2.41, df = 3 (P = 0.49); I² = 0%									
Test for overall effect: Z = 6.80 (P < 0.00001)									
**1.18.2 High: ≥21**									
Hui 2014	12.9	3.72	30	16.23	4.38	27	2.4%	-3.33 [-5.45, -1.21]	2014
Sagedal 2016	14.7	5.1	189	15.8	5.4	206	8.4%	-1.10 [-2.14, -0.06]	2016
Kunath 2019	14.6	4.5	379	14.8	4.6	385	16.1%	-0.20 [-0.85, 0.45]	2019
Buckingham-Schutt 2019	11.8	3.3	12	12.7	4.8	16	1.2%	-0.90 [-3.90, 2.10]	2019
Atkinson 2022	11.02	3.4	58	11.43	3.67	61	6.0%	-0.41 [-1.68, 0.86]	2022
Yang 2023	14.52	4.52	178	15.82	4.35	177	10.0%	-1.30 [-2.22, -0.38]	2023
**Subtotal (95% CI)**			**846**			**872**	**44.1%**	**-0.96 [-1.67, -0.25]**	
Heterogeneity: Tau² = 0.37; Chi² = 10.56, df = 5 (P = 0.06); I² = 53%									
Test for overall effect: Z = 2.65 (P = 0.008)									
**Total (95% CI)**			**1608**			**1616**	**100.0%**	**-0.78 [-1.12, -0.44]**	
Heterogeneity: Tau² = 0.08; Chi² = 13.04, df = 9 (P = 0.16); I² = 31%									
Test for overall effect: Z = 4.53 (P < 0.00001)									
Test for subgroup differences: Chi² = 0.14, df = 1 (P = 0.71), I² = 0%									

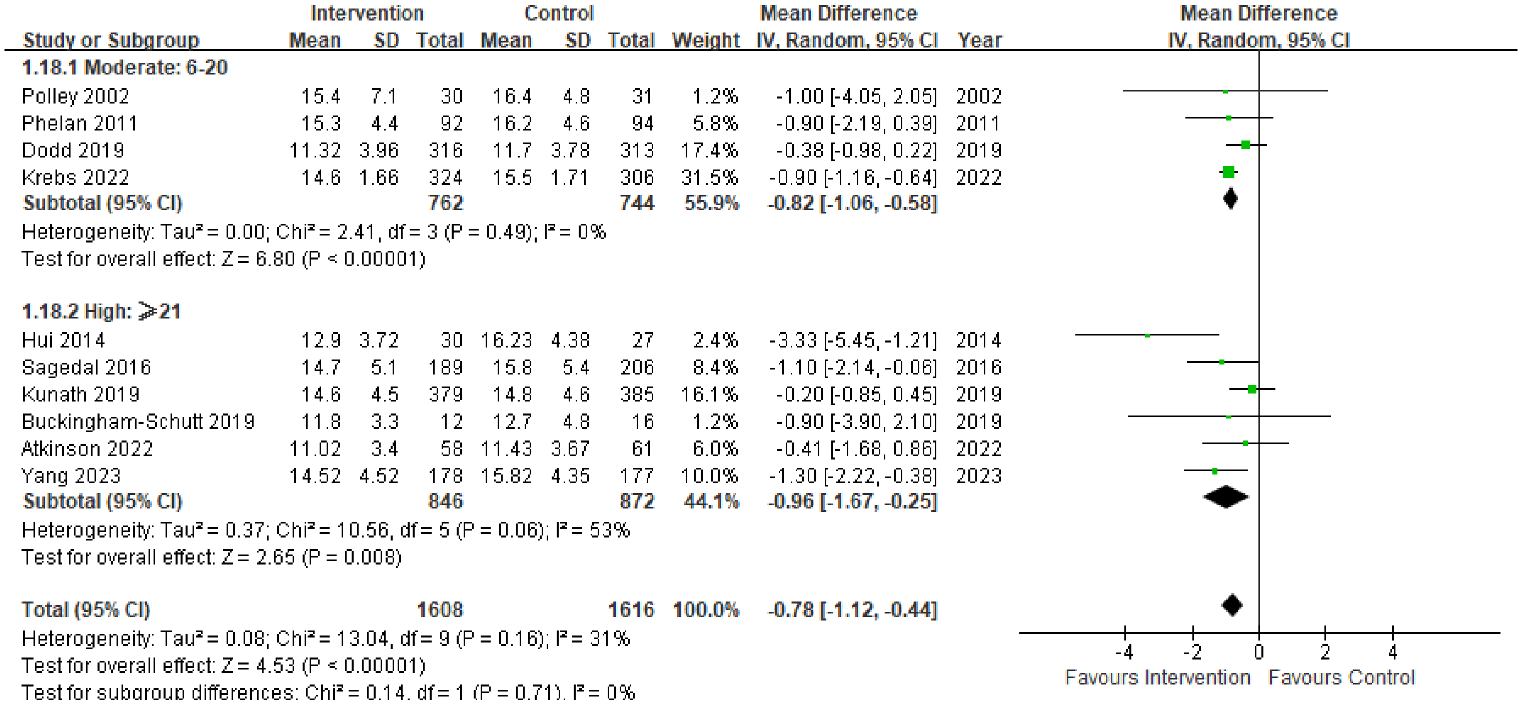

Figure S17. Forest plot of Effect of **Tailoring** in the physical activity component on total GWG

Study or Subgroup	Intervention Mean	Intervention SD	Intervention Total	Control Mean	Control SD	Control Total	Weight	Mean Difference IV, Random, 95% CI	Year
**1.21.1 With tailoring**									
Polley 2002	15.4	7.1	30	16.4	4.8	31	1.2%	-1.00 [-4.05, 2.05]	2002
Dodd 2019	11.32	3.96	316	11.7	3.78	313	17.4%	-0.38 [-0.98, 0.22]	2019
Kunath 2019	14.6	4.5	379	14.8	4.6	385	16.1%	-0.20 [-0.85, 0.45]	2019
Atkinson 2022	11.02	3.4	58	11.43	3.67	61	6.0%	-0.41 [-1.68, 0.86]	2022
Krebs 2022	14.6	1.66	324	15.5	1.71	306	31.5%	-0.90 [-1.16, -0.64]	2022
Yang 2023	14.52	4.52	178	15.82	4.35	177	10.0%	-1.30 [-2.22, -0.38]	2023
**Subtotal (95% CI)**			**1285**			**1273**	**82.2%**	**-0.68 [-1.01, -0.34]**	
Heterogeneity: Tau² = 0.05; Chi² = 7.12, df = 5 (P = 0.21); I² = 30%									
Test for overall effect: Z = 3.97 (P < 0.0001)									
**1.21.2 Without tailoring**									
Phelan 2011	15.3	4.4	92	16.2	4.6	94	5.8%	-0.90 [-2.19, 0.39]	2011
Hui 2014	12.9	3.72	30	16.23	4.38	27	2.4%	-3.33 [-5.45, -1.21]	2014
Sagedal 2016	14.7	5.1	189	15.8	5.4	206	8.4%	-1.10 [-2.14, -0.06]	2016
Buckingham-Schutt 2019	11.8	3.3	12	12.7	4.8	16	1.2%	-0.90 [-3.90, 2.10]	2019
**Subtotal (95% CI)**			**323**			**343**	**17.8%**	**-1.37 [-2.29, -0.44]**	
Heterogeneity: Tau² = 0.24; Chi² = 4.09, df = 3 (P = 0.25); I² = 27%									
Test for overall effect: Z = 2.90 (P = 0.004)									
**Total (95% CI)**			**1608**			**1616**	**100.0%**	**-0.78 [-1.12, -0.44]**	
Heterogeneity: Tau² = 0.08; Chi² = 13.04, df = 9 (P = 0.16); I² = 31%									
Test for overall effect: Z = 4.53 (P < 0.00001)									
Test for subgroup differences: Chi² = 1.88, df = 1 (P = 0.17), I² = 46.9%									

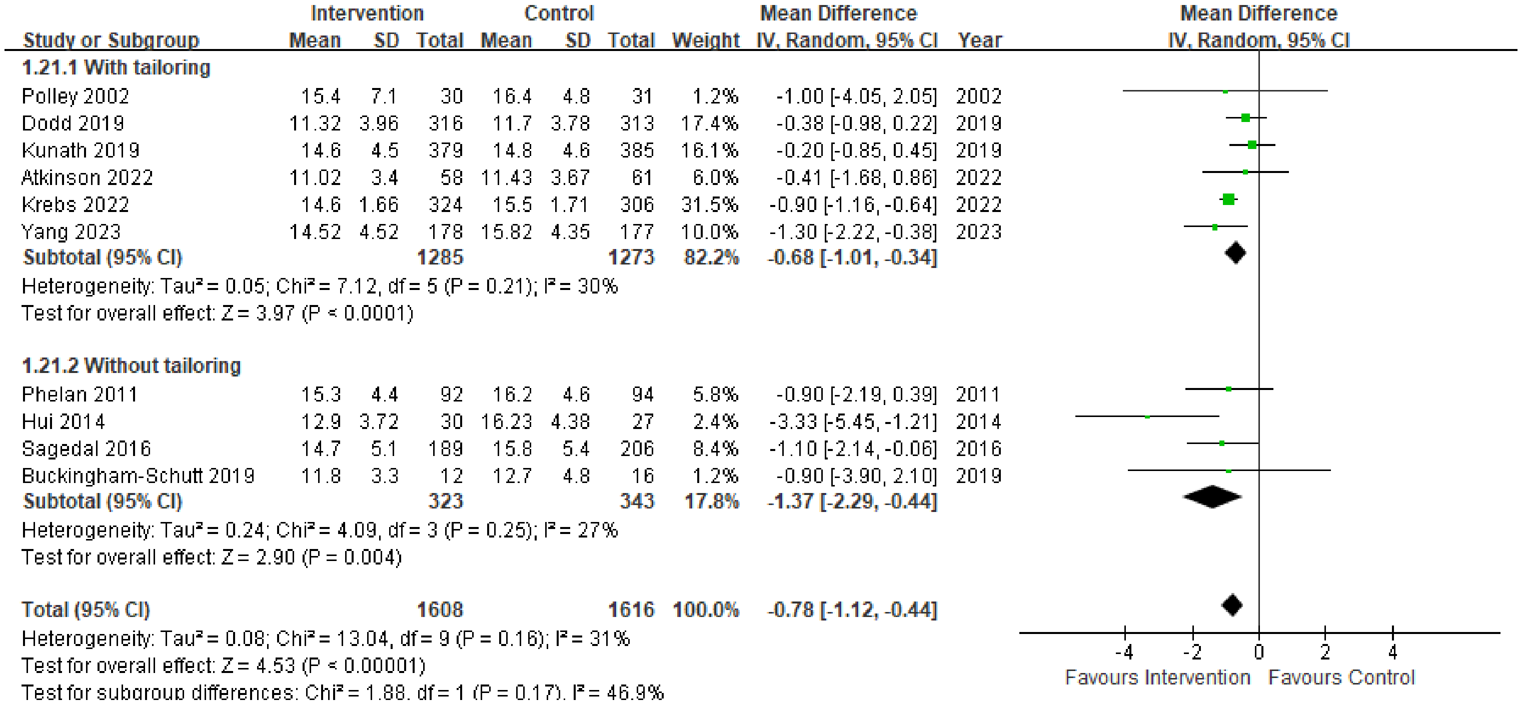

Figure S18. Forest plot of Effect of **Resource** in the diet component on total GWG

Study or Subgroup	Intervention Mean	Intervention SD	Intervention Total	Control Mean	Control SD	Control Total	Weight	Mean Difference IV, Random, 95% CI	Year
**1.4.1 Other resource**									
Polley 2002	15.4	7.1	30	16.4	4.8	31	1.2%	-1.00 [-4.05, 2.05]	2002
Hui 2014	12.9	3.72	30	16.23	4.38	27	2.4%	-3.33 [-5.45, -1.21]	2014
Sagedal 2016	14.7	5.1	189	15.8	5.4	206	8.4%	-1.10 [-2.14, -0.06]	2016
Dodd 2019	11.32	3.96	316	11.7	3.78	313	17.4%	-0.38 [-0.98, 0.22]	2019
Krebs 2022	14.6	1.66	324	15.5	1.71	306	31.5%	-0.90 [-1.16, -0.64]	2022
Atkinson 2022	11.02	3.4	58	11.43	3.67	61	6.0%	-0.41 [-1.68, 0.86]	2022
Yang 2023	14.52	4.52	178	15.82	4.35	177	10.0%	-1.30 [-2.22, -0.38]	2023
**Subtotal (95% CI)**			**1125**			**1121**	**76.8%**	**-0.90 [-1.30, -0.50]**	
Heterogeneity: Tau² = 0.09; Chi² = 9.27, df = 6 (P = 0.16); I² = 35%									
Test for overall effect: Z = 4.38 (P < 0.0001)									
**1.4.2 Combination**									
Phelan 2011	15.3	4.4	92	16.2	4.6	94	5.8%	-0.90 [-2.19, 0.39]	2011
Kunath 2019	14.6	4.5	379	14.8	4.6	385	16.1%	-0.20 [-0.85, 0.45]	2019
Buckingham-Schutt 2019	11.8	3.3	12	12.7	4.8	16	1.2%	-0.90 [-3.90, 2.10]	2019
**Subtotal (95% CI)**			**483**			**495**	**23.2%**	**-0.36 [-0.93, 0.21]**	
Heterogeneity: Tau² = 0.00; Chi² = 1.03, df = 2 (P = 0.60); I² = 0%									
Test for overall effect: Z = 1.24 (P = 0.21)									
**Total (95% CI)**			**1608**			**1616**	**100.0%**	**-0.78 [-1.12, -0.44]**	
Heterogeneity: Tau² = 0.08; Chi² = 13.04, df = 9 (P = 0.16); I² = 31%									
Test for overall effect: Z = 4.53 (P < 0.00001)									
Test for subgroup differences: Chi² = 2.33, df = 1 (P = 0.13), I² = 57.1%									

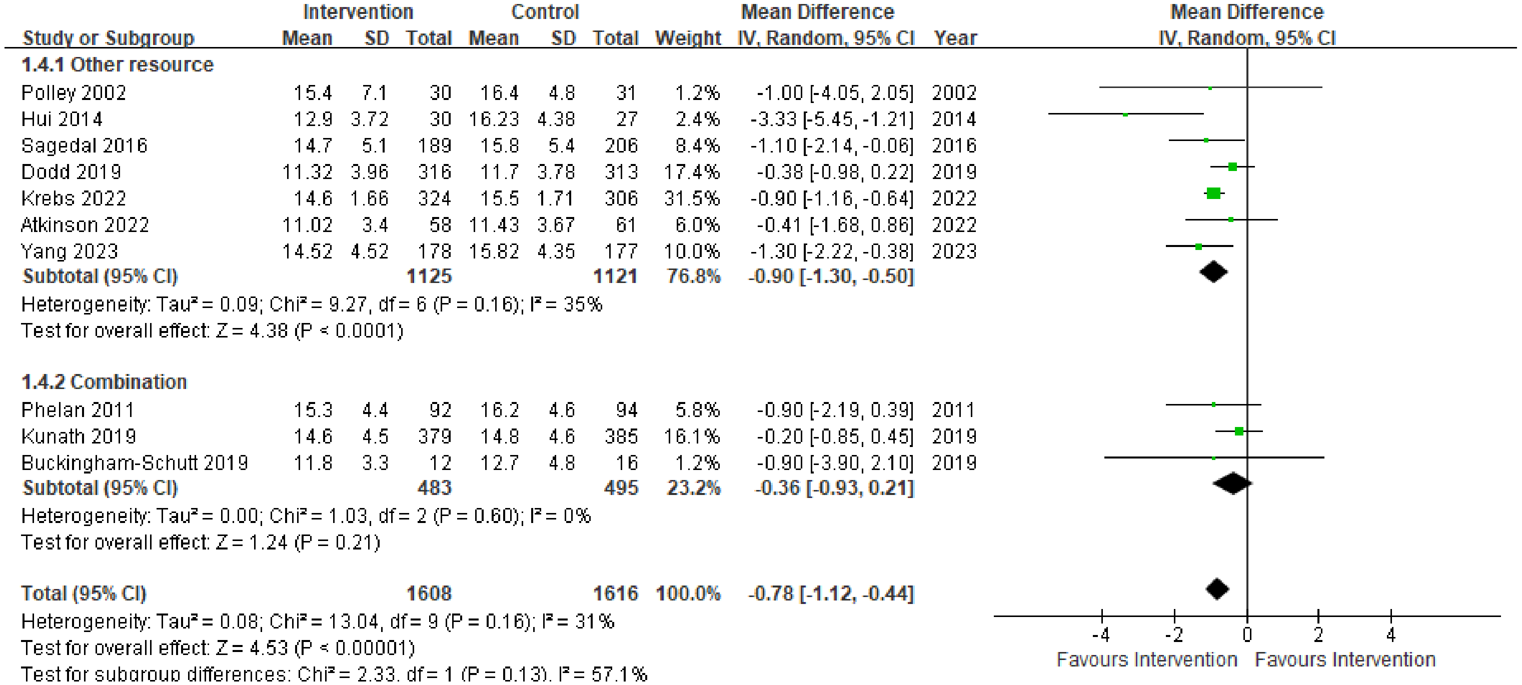

Figure S19. Forest plot of Effect of **Resource** in the physical activity component on total GWG

	Intervention			Control				Mean Difference	
Study or Subgroup	Mean	SD	Total	Mean	SD	Total	Weight	IV, Random, 95% CI	Year
**1.13.1 Self-monitoring tool**									
Atkinson 2022	11.02	3.4	58	11.43	3.67	61	6.0%	-0.41 [-1.68, 0.86]	2022
**Subtotal (95% CI)**			**58**			**61**	**6.0%**	**-0.41 [-1.68, 0.86]**	
Heterogeneity: Not applicable									
Test for overall effect: Z = 0.63 (P = 0.53)									
**1.13.2 Other resource**									
Polley 2002	15.4	7.1	30	16.4	4.8	31	1.2%	-1.00 [-4.05, 2.05]	2002
Hui 2014	12.9	3.72	30	16.23	4.38	27	2.4%	-3.33 [-5.45, -1.21]	2014
Sagedal 2016	14.7	5.1	189	15.8	5.4	206	8.4%	-1.10 [-2.14, -0.06]	2016
Dodd 2019	11.32	3.96	316	11.7	3.78	313	17.4%	-0.38 [-0.98, 0.22]	2019
Krebs 2022	14.6	1.66	324	15.5	1.71	306	31.5%	-0.90 [-1.16, -0.64]	2022
Yang 2023	14.52	4.52	178	15.82	4.35	177	10.0%	-1.30 [-2.22, -0.38]	2023
**Subtotal (95% CI)**			**1067**			**1060**	**70.8%**	**-0.96 [-1.41, -0.51]**	
Heterogeneity: Tau² = 0.11; Chi² = 8.74, df = 5 (P = 0.12); I² = 43%									
Test for overall effect: Z = 4.18 (P < 0.0001)									
**1.13.3 Combination**									
Phelan 2011	15.3	4.4	92	16.2	4.6	94	5.8%	-0.90 [-2.19, 0.39]	2011
Kunath 2019	14.6	4.5	379	14.8	4.6	385	16.1%	-0.20 [-0.85, 0.45]	2019
Buckingham-Schutt 2019	11.8	3.3	12	12.7	4.8	16	1.2%	-0.90 [-3.90, 2.10]	2019
**Subtotal (95% CI)**			**483**			**495**	**23.2%**	**-0.36 [-0.93, 0.21]**	
Heterogeneity: Tau² = 0.00; Chi² = 1.03, df = 2 (P = 0.60); I² = 0%									
Test for overall effect: Z = 1.24 (P = 0.21)									
**Total (95% CI)**			**1608**			**1616**	**100.0%**	**-0.78 [-1.12, -0.44]**	
Heterogeneity: Tau² = 0.08; Chi² = 13.04, df = 9 (P = 0.16); I² = 31%									
Test for overall effect: Z = 4.53 (P < 0.00001)									
Test for subgroup differences: Chi² = 2.87, df = 2 (P = 0.24), I² = 30.4%									

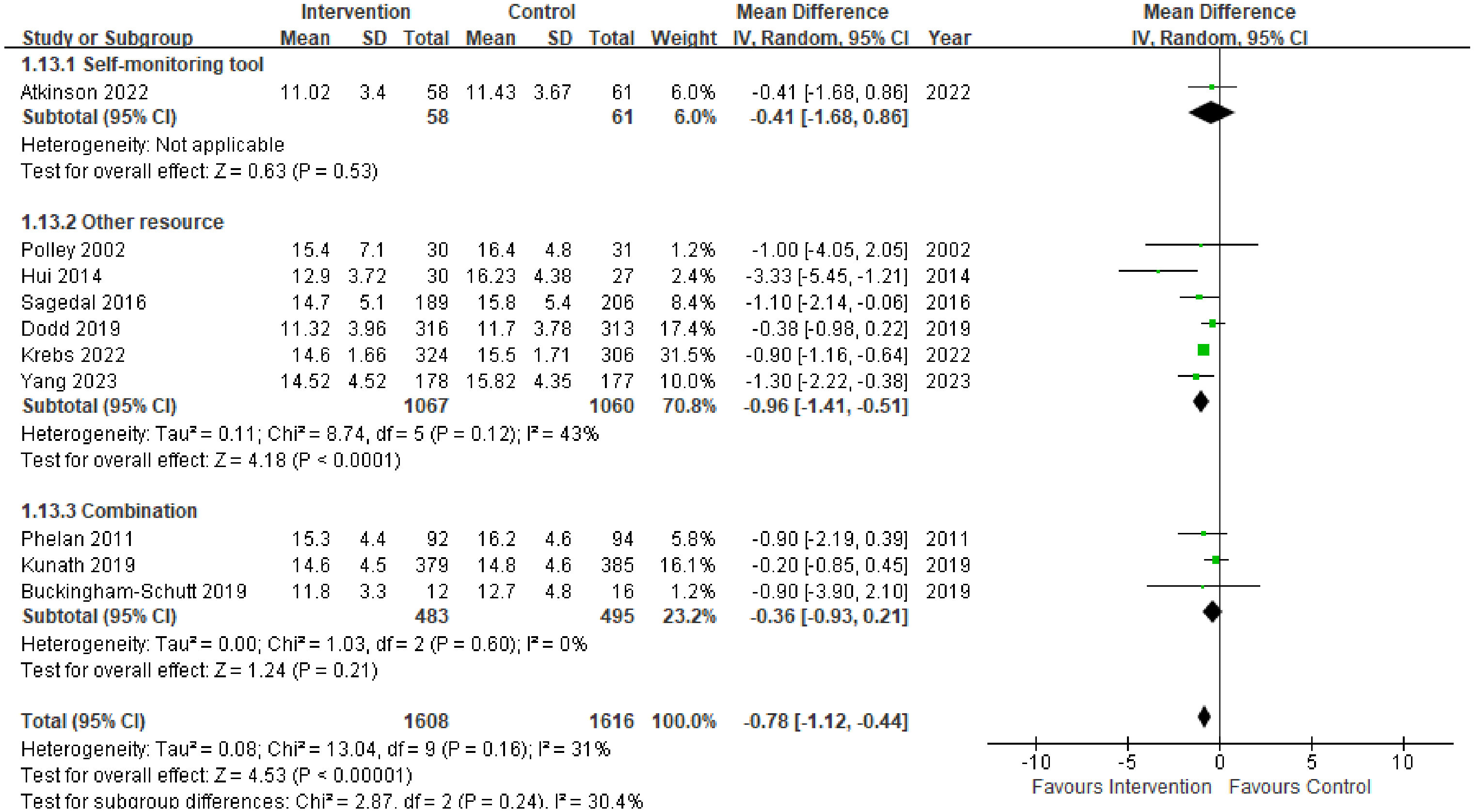

3. Forest plot of subgroup analyses of combined diet with physical activity on EGWG based on TIDieR checklist

Figure S20. Forest plot of the effect of **Timing of Intervention Initiation** on EGWG

Study or Subgroup	Intervention Events	Intervention Total	Control Events	Control Total	Weight	Odds Ratio M-H, Random, 95% CI	Year
**2.2.1 First trimester**							
Krebs 2022	158	324	176	306	22.8%	0.70 [0.51, 0.96]	2022
Yang 2023	51	178	79	177	16.9%	0.50 [0.32, 0.77]	2023
**Subtotal (95% CI)**		**502**		**483**	**39.8%**	**0.61 [0.44, 0.85]**	
Total events	209		255				
Heterogeneity: Tau² = 0.02; Chi² = 1.56, df = 1 (P = 0.21); I² = 36%							
Test for overall effect: Z = 2.90 (P = 0.004)							
**2.2.2 Second trimester**							
Polley 2002	10	30	18	31	4.9%	0.36 [0.13, 1.02]	2002
Phelan 2011	37	92	49	94	12.2%	0.62 [0.35, 1.10]	2011
Hui 2014	3	30	10	27	2.8%	0.19 [0.05, 0.79]	2014
Kunath 2019	130	379	138	385	23.7%	0.93 [0.69, 1.26]	2019
Dodd 2019	28	316	41	313	14.4%	0.64 [0.39, 1.07]	2019
Buckingham-Schutt 2019	3	12	8	16	2.2%	0.33 [0.07, 1.71]	2019
**Subtotal (95% CI)**		**859**		**866**	**60.2%**	**0.61 [0.42, 0.90]**	
Total events	211		264				
Heterogeneity: Tau² = 0.09; Chi² = 9.28, df = 5 (P = 0.10); I² = 46%							
Test for overall effect: Z = 2.51 (P = 0.01)							
**Total (95% CI)**		**1361**		**1349**	**100.0%**	**0.63 [0.49, 0.81]**	
Total events	420		519				
Heterogeneity: Tau² = 0.05; Chi² = 11.74, df = 7 (P = 0.11); I² = 40%							
Test for overall effect: Z = 3.59 (P = 0.0003)							
Test for subgroup differences: Chi² = 0.00, df = 1 (P = 1.00), I² = 0%							

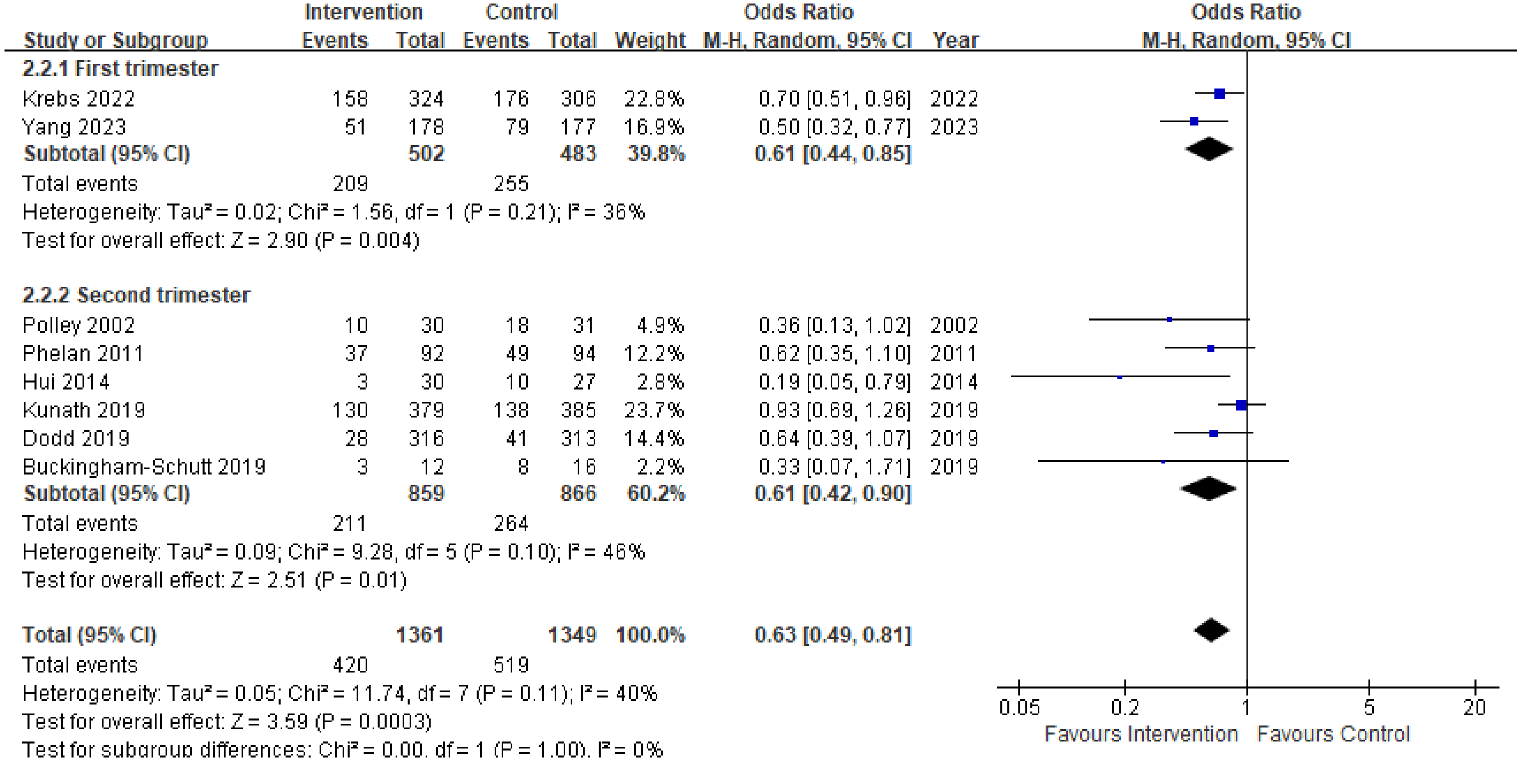

Figure S21. Forest plot of the effect of **Theory** on EGWG

Study or Subgroup	Intervention Events	Intervention Total	Control Events	Control Total	Weight	Odds Ratio M-H, Random, 95% CI	Year
**2.3.1 Theory based**							
Phelan 2011	37	92	49	94	12.2%	0.62 [0.35, 1.10]	2011
Dodd 2019	28	316	41	313	14.4%	0.64 [0.39, 1.07]	2019
**Subtotal (95% CI)**		**408**		**407**	**26.6%**	**0.63 [0.43, 0.93]**	
Total events	65		90				
Heterogeneity: Tau² = 0.00; Chi² = 0.01, df = 1 (P = 0.91); I² = 0%							
Test for overall effect: Z = 2.34 (P = 0.02)							
**2.3.2 Non-theory based**							
Polley 2002	10	30	18	31	4.9%	0.36 [0.13, 1.02]	2002
Hui 2014	3	30	10	27	2.8%	0.19 [0.05, 0.79]	2014
Kunath 2019	130	379	138	385	23.7%	0.93 [0.69, 1.26]	2019
Buckingham-Schutt 2019	3	12	8	16	2.2%	0.33 [0.07, 1.71]	2019
Krebs 2022	158	324	176	306	22.8%	0.70 [0.51, 0.96]	2022
Yang 2023	51	178	79	177	16.9%	0.50 [0.32, 0.77]	2023
**Subtotal (95% CI)**		**953**		**942**	**73.4%**	**0.60 [0.42, 0.86]**	
Total events	355		429				
Heterogeneity: Tau² = 0.09; Chi² = 11.52, df = 5 (P = 0.04); I² = 57%							
Test for overall effect: Z = 2.82 (P = 0.005)							
**Total (95% CI)**		**1361**		**1349**	**100.0%**	**0.63 [0.49, 0.81]**	
Total events	420		519				
Heterogeneity: Tau² = 0.05; Chi² = 11.74, df = 7 (P = 0.11); I² = 40%							
Test for overall effect: Z = 3.59 (P = 0.0003)							
Test for subgroup differences: Chi² = 0.04, df = 1 (P = 0.84), I² = 0%							

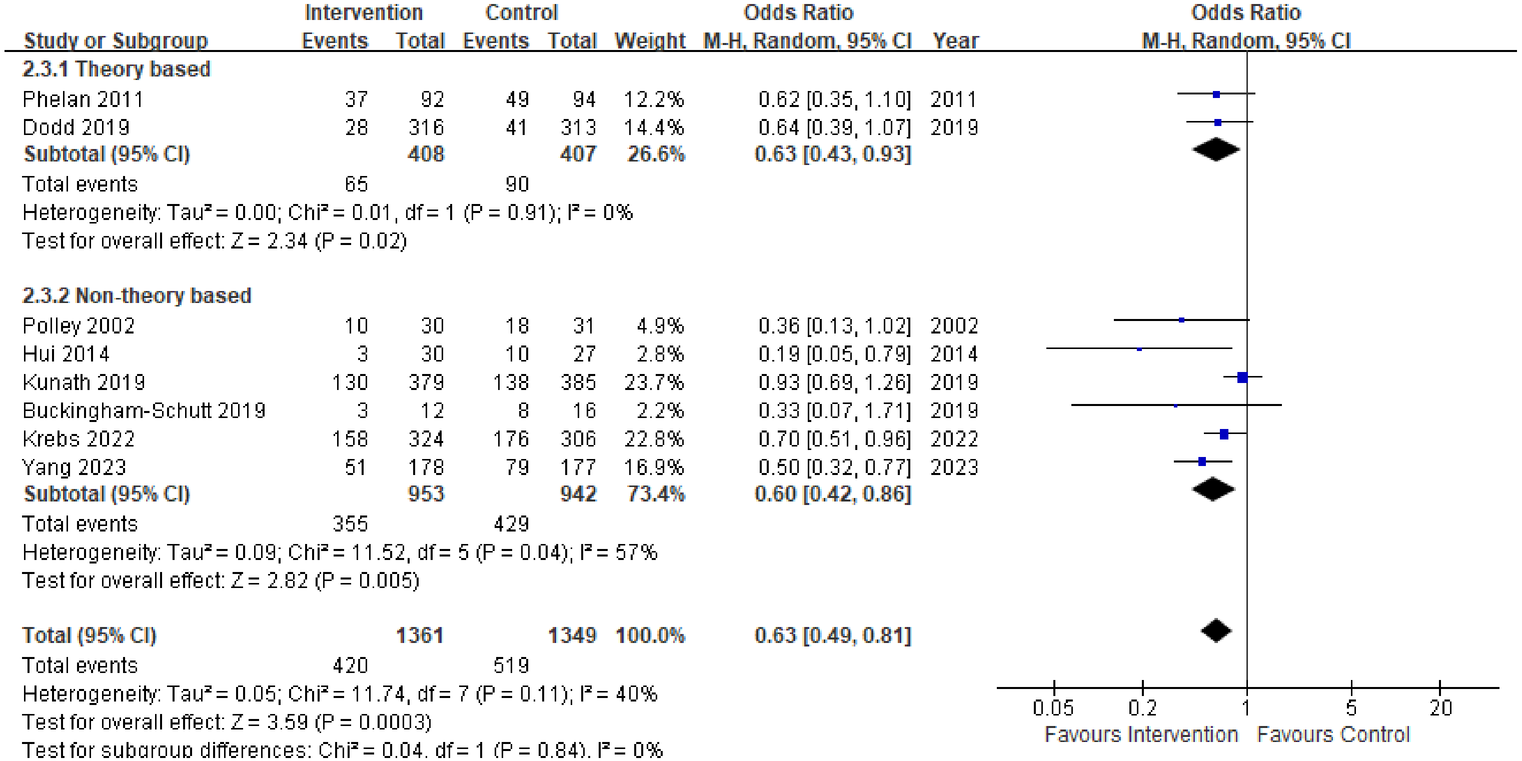

Figure S22. Forest plot of the effect of **Feedback** on EGWG

Study or Subgroup	Intervention Events	Intervention Total	Control Events	Control Total	Weight	Odds Ratio M-H, Random, 95% CI	Year
**2.12.1 With feedback**							
Polley 2002	10	30	18	31	4.9%	0.36 [0.13, 1.02]	2002
Phelan 2011	37	92	49	94	12.2%	0.62 [0.35, 1.10]	2011
Hui 2014	3	30	10	27	2.8%	0.19 [0.05, 0.79]	2014
Dodd 2019	28	316	41	313	14.4%	0.64 [0.39, 1.07]	2019
Buckingham-Schutt 2019	3	12	8	16	2.2%	0.33 [0.07, 1.71]	2019
Kunath 2019	130	379	138	385	23.7%	0.93 [0.69, 1.26]	2019
Yang 2023	51	178	79	177	16.9%	0.50 [0.32, 0.77]	2023
**Subtotal (95% CI)**		**1037**		**1043**	**77.2%**	**0.59 [0.42, 0.82]**	
Total events	262		343				
Heterogeneity: Tau² = 0.08; Chi² = 11.72, df = 6 (P = 0.07); I² = 49%							
Test for overall effect: Z = 3.13 (P = 0.002)							
**2.12.2 Without feedback**							
Krebs 2022	158	324	176	306	22.8%	0.70 [0.51, 0.96]	2022
**Subtotal (95% CI)**		**324**		**306**	**22.8%**	**0.70 [0.51, 0.96]**	
Total events	158		176				
Heterogeneity: Not applicable							
Test for overall effect: Z = 2.20 (P = 0.03)							
**Total (95% CI)**		**1361**		**1349**	**100.0%**	**0.63 [0.49, 0.81]**	
Total events	420		519				
Heterogeneity: Tau² = 0.05; Chi² = 11.74, df = 7 (P = 0.11); I² = 40%							
Test for overall effect: Z = 3.59 (P = 0.0003)							
Test for subgroup differences: Chi² = 0.57, df = 1 (P = 0.45), I² = 0%							

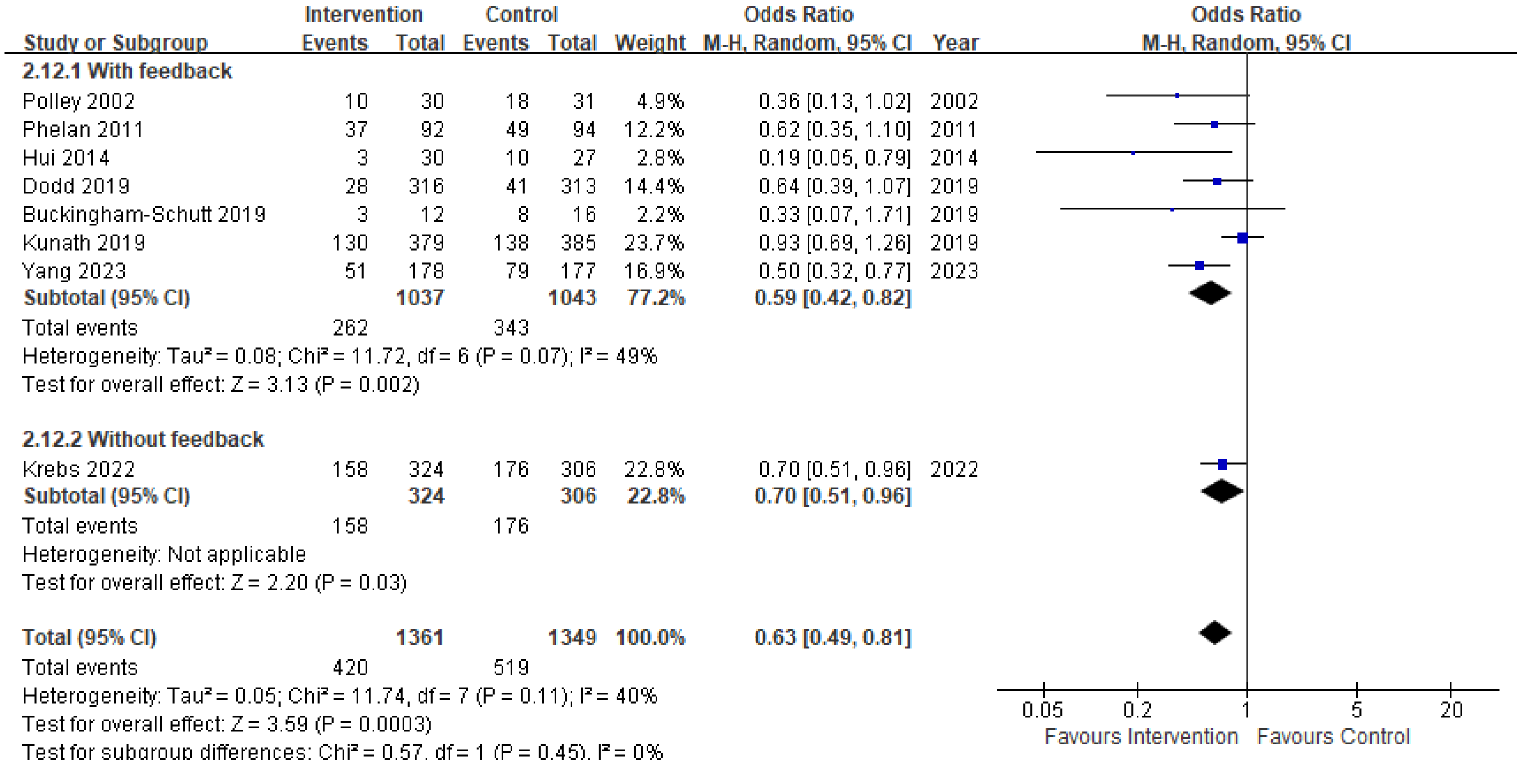

Figure S23. Forest plot of Effect of **Delivery Mode** in the diet component on EGWG

Study or Subgroup	Intervention Events	Intervention Total	Control Events	Control Total	Weight	Odds Ratio M-H, Random, 95% CI	Year
**2.6.1 Face to face**							
Hui 2014	3	30	10	27	2.8%	0.19 [0.05, 0.79]	2014
Kunath 2019	130	379	138	385	23.7%	0.93 [0.69, 1.26]	2019
Krebs 2022	158	324	176	306	22.8%	0.70 [0.51, 0.96]	2022
**Subtotal (95% CI)**		**733**		**718**	**49.3%**	**0.72 [0.47, 1.12]**	
Total events	291		324				
Heterogeneity: Tau² = 0.08; Chi² = 5.63, df = 2 (P = 0.06); I² = 64%							
Test for overall effect: Z = 1.47 (P = 0.14)							
**2.6.2 Face to face and remote**							
Polley 2002	10	30	18	31	4.9%	0.36 [0.13, 1.02]	2002
Phelan 2011	37	92	49	94	12.2%	0.62 [0.35, 1.10]	2011
Dodd 2019	28	316	41	313	14.4%	0.64 [0.39, 1.07]	2019
Buckingham-Schutt 2019	3	12	8	16	2.2%	0.33 [0.07, 1.71]	2019
Yang 2023	51	178	79	177	16.9%	0.50 [0.32, 0.77]	2023
**Subtotal (95% CI)**		**628**		**631**	**50.7%**	**0.54 [0.41, 0.72]**	
Total events	129		195				
Heterogeneity: Tau² = 0.00; Chi² = 1.71, df = 4 (P = 0.79); I² = 0%							
Test for overall effect: Z = 4.34 (P < 0.0001)							
**Total (95% CI)**		**1361**		**1349**	**100.0%**	**0.63 [0.49, 0.81]**	
Total events	420		519				
Heterogeneity: Tau² = 0.05; Chi² = 11.74, df = 7 (P = 0.11); I² = 40%							
Test for overall effect: Z = 3.59 (P = 0.0003)							
Test for subgroup differences: Chi² = 1.15, df = 1 (P = 0.28), I² = 13.3%							

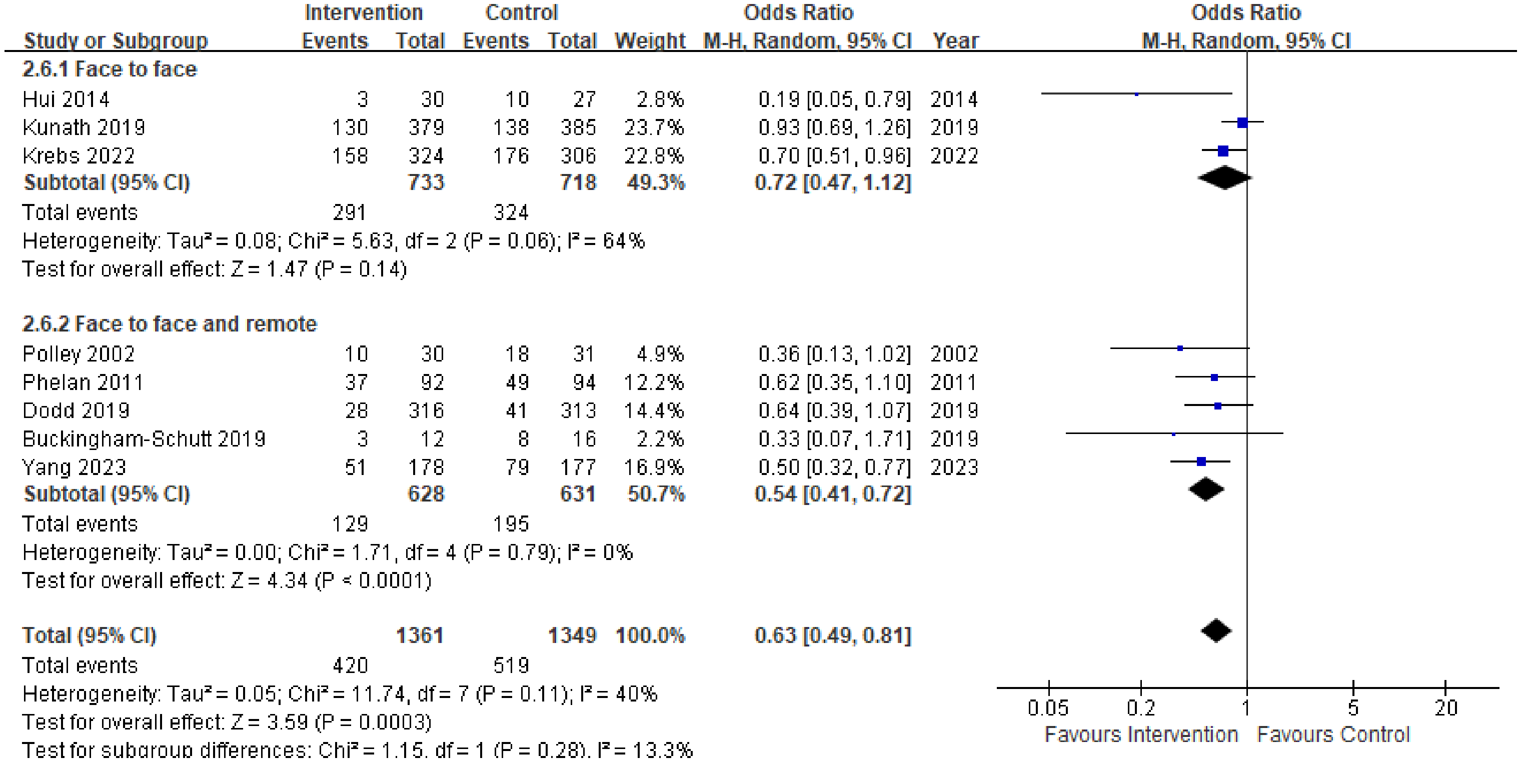

Figure S24. Forest plot of Effect of **Delivery Mode** in the physical activity component on EGWG

Study or Subgroup	Intervention Events	Intervention Total	Control Events	Control Total	Weight	Odds Ratio M-H, Random, 95% CI	Year
**2.17.1 Face to face**							
Hui 2014	3	30	10	27	2.8%	0.19 [0.05, 0.79]	2014
Kunath 2019	130	379	138	385	23.7%	0.93 [0.69, 1.26]	2019
Krebs 2022	158	324	176	306	22.8%	0.70 [0.51, 0.96]	2022
**Subtotal (95% CI)**		**733**		**718**	**49.3%**	**0.72 [0.47, 1.12]**	
Total events	291		324				
Heterogeneity: Tau² = 0.08; Chi² = 5.63, df = 2 (P = 0.06); I² = 64%							
Test for overall effect: Z = 1.47 (P = 0.14)							
**2.17.2 Face to face and remote**							
Polley 2002	10	30	18	31	4.9%	0.36 [0.13, 1.02]	2002
Phelan 2011	37	92	49	94	12.2%	0.62 [0.35, 1.10]	2011
Dodd 2019	28	316	41	313	14.4%	0.64 [0.39, 1.07]	2019
Buckingham-Schutt 2019	3	12	8	16	2.2%	0.33 [0.07, 1.71]	2019
Yang 2023	51	178	79	177	16.9%	0.50 [0.32, 0.77]	2023
**Subtotal (95% CI)**		**628**		**631**	**50.7%**	**0.54 [0.41, 0.72]**	
Total events	129		195				
Heterogeneity: Tau² = 0.00; Chi² = 1.71, df = 4 (P = 0.79); I² = 0%							
Test for overall effect: Z = 4.34 (P < 0.0001)							
**Total (95% CI)**		**1361**		**1349**	**100.0%**	**0.63 [0.49, 0.81]**	
Total events	420		519				
Heterogeneity: Tau² = 0.05; Chi² = 11.74, df = 7 (P = 0.11); I² = 40%							
Test for overall effect: Z = 3.59 (P = 0.0003)							
Test for subgroup differences: Chi² = 1.15, df = 1 (P = 0.28), I² = 13.3%							

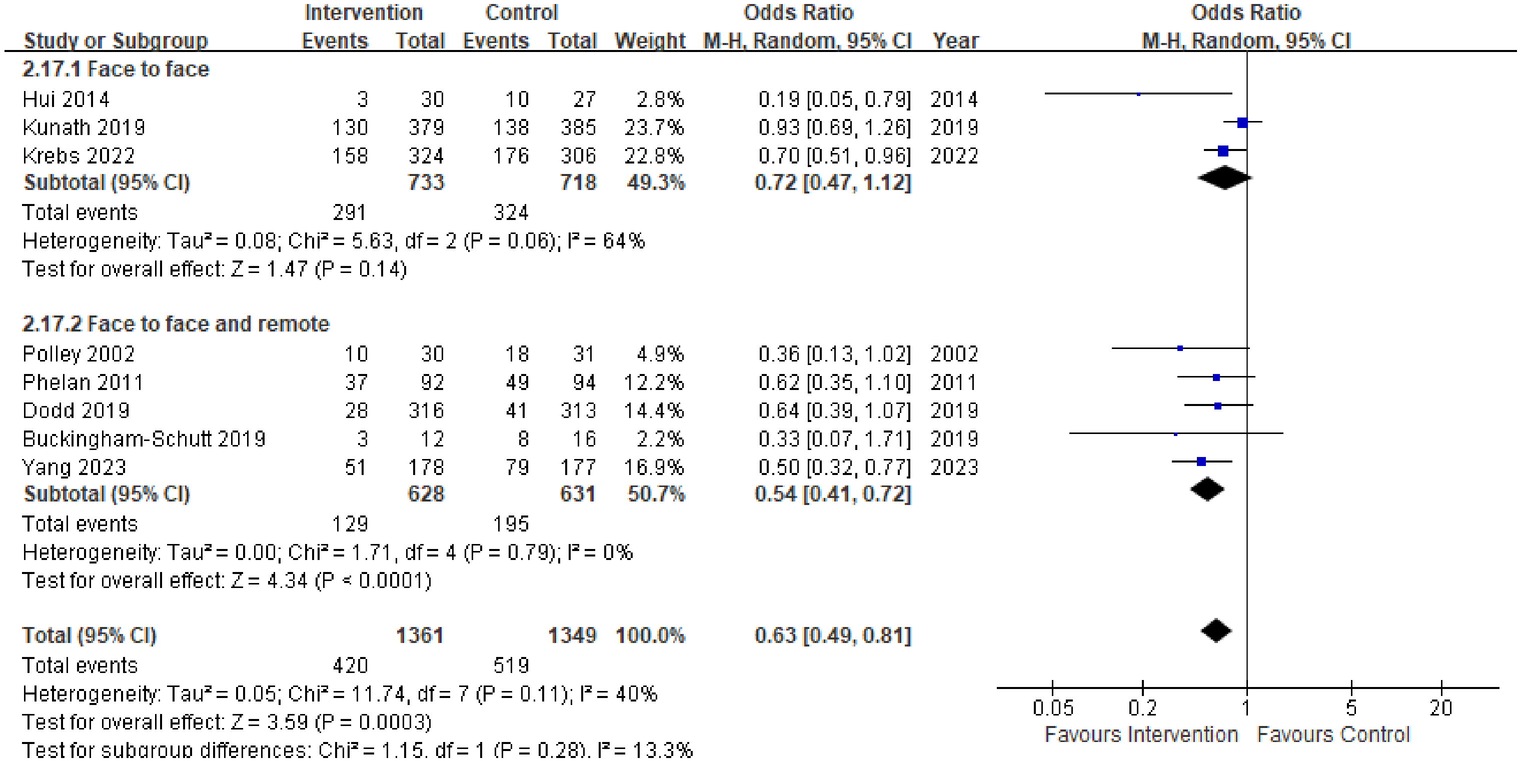

Figure S25. Forest plot of Effect of **Intervention format** in the physical activity component on EGWG

Study or Subgroup	Intervention Events	Intervention Total	Control Events	Control Total	Weight	Odds Ratio M-H, Random, 95% CI	Year
**2.18.1 Individual**							
Polley 2002	10	30	18	31	4.9%	0.36 [0.13, 1.02]	2002
Phelan 2011	37	92	49	94	12.2%	0.62 [0.35, 1.10]	2011
Dodd 2019	28	316	41	313	14.4%	0.64 [0.39, 1.07]	2019
Buckingham-Schutt 2019	3	12	8	16	2.2%	0.33 [0.07, 1.71]	2019
Kunath 2019	130	379	138	385	23.7%	0.93 [0.69, 1.26]	2019
Krebs 2022	158	324	176	306	22.8%	0.70 [0.51, 0.96]	2022
Yang 2023	51	178	79	177	16.9%	0.50 [0.32, 0.77]	2023
**Subtotal (95% CI)**		**1331**		**1322**	**97.2%**	**0.67 [0.54, 0.83]**	
Total events	417		509				
Heterogeneity: Tau² = 0.02; Chi² = 8.55, df = 6 (P = 0.20); I² = 30%							
Test for overall effect: Z = 3.56 (P = 0.0004)							
**2.18.2 Individual and group**							
Hui 2014	3	30	10	27	2.8%	0.19 [0.05, 0.79]	2014
**Subtotal (95% CI)**		**30**		**27**	**2.8%**	**0.19 [0.05, 0.79]**	
Total events	3		10				
Heterogeneity: Not applicable							
Test for overall effect: Z = 2.29 (P = 0.02)							
**Total (95% CI)**		**1361**		**1349**	**100.0%**	**0.63 [0.49, 0.81]**	
Total events	420		519				
Heterogeneity: Tau² = 0.05; Chi² = 11.74, df = 7 (P = 0.11); I² = 40%							
Test for overall effect: Z = 3.59 (P = 0.0003)							
Test for subgroup differences: Chi² = 2.95, df = 1 (P = 0.09), I² = 66.1%							

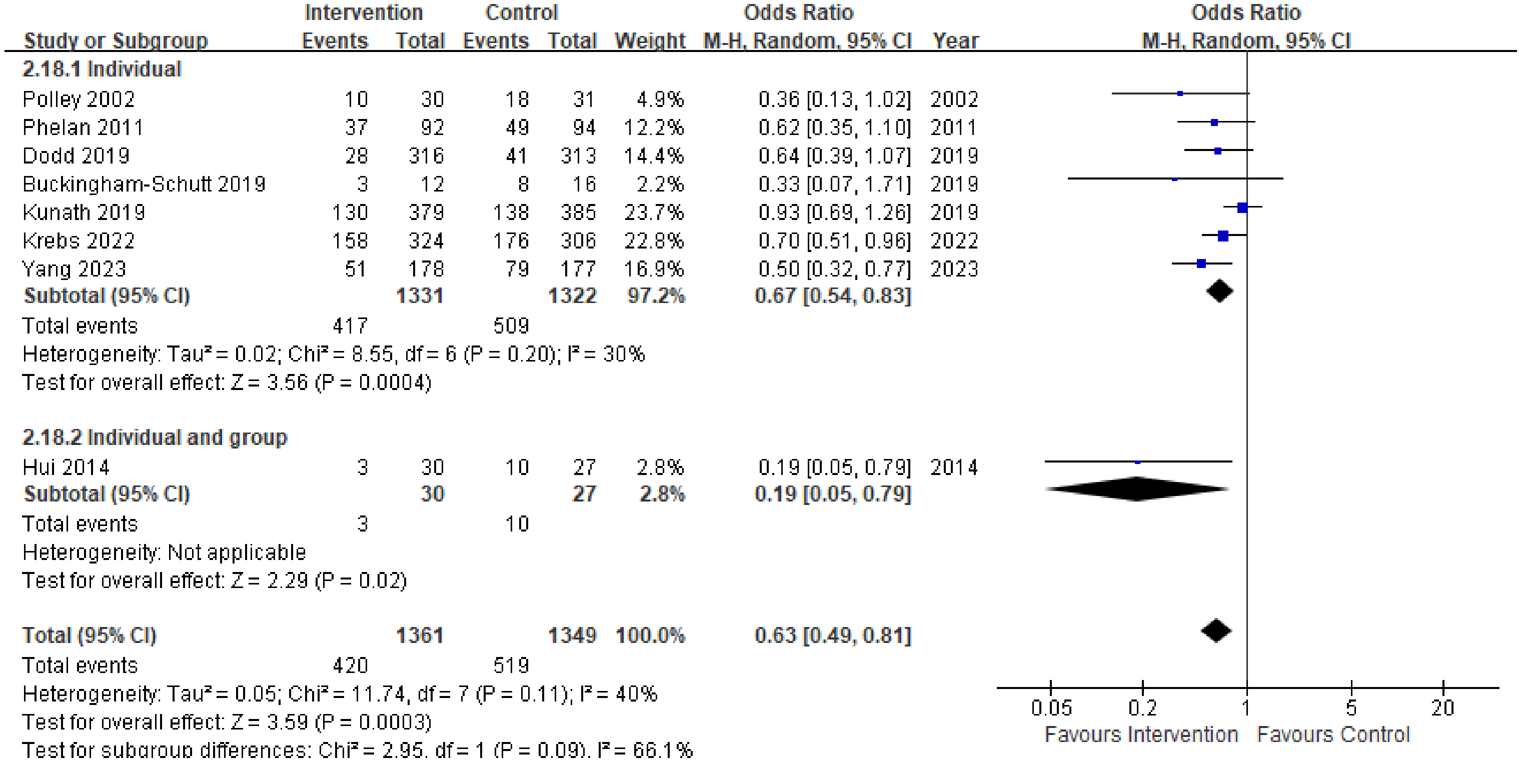

Figure S26. Forest plot of Effect of **Provider** in the diet component on EGWG

Study or Subgroup	Intervention Events	Intervention Total	Control Events	Control Total	Weight	Odds Ratio M-H, Random, 95% CI	Year
**2.8.1 Nutritionist**							
Phelan 2011	37	92	49	94	12.2%	0.62 [0.35, 1.10]	2011
Hui 2014	3	30	10	27	2.8%	0.19 [0.05, 0.79]	2014
Buckingham-Schutt 2019	3	12	8	16	2.2%	0.33 [0.07, 1.71]	2019
Dodd 2019	28	316	41	313	14.4%	0.64 [0.39, 1.07]	2019
**Subtotal (95% CI)**		**450**		**450**	**31.6%**	**0.57 [0.39, 0.82]**	
Total events	71		108				
Heterogeneity: Tau² = 0.00; Chi² = 3.02, df = 3 (P = 0.39); I² = 1%							
Test for overall effect: Z = 3.06 (P = 0.002)							
**2.8.2 Other**							
Polley 2002	10	30	18	31	4.9%	0.36 [0.13, 1.02]	2002
Kunath 2019	130	379	138	385	23.7%	0.93 [0.69, 1.26]	2019
Krebs 2022	158	324	176	306	22.8%	0.70 [0.51, 0.96]	2022
Yang 2023	51	178	79	177	16.9%	0.50 [0.32, 0.77]	2023
**Subtotal (95% CI)**		**911**		**899**	**68.4%**	**0.67 [0.48, 0.93]**	
Total events	349		411				
Heterogeneity: Tau² = 0.06; Chi² = 7.35, df = 3 (P = 0.06); I² = 59%							
Test for overall effect: Z = 2.36 (P = 0.02)							
**Total (95% CI)**		**1361**		**1349**	**100.0%**	**0.63 [0.49, 0.81]**	
Total events	420		519				
Heterogeneity: Tau² = 0.05; Chi² = 11.74, df = 7 (P = 0.11); I² = 40%							
Test for overall effect: Z = 3.59 (P = 0.0003)							
Test for subgroup differences: Chi² = 0.44, df = 1 (P = 0.51), I² = 0%							

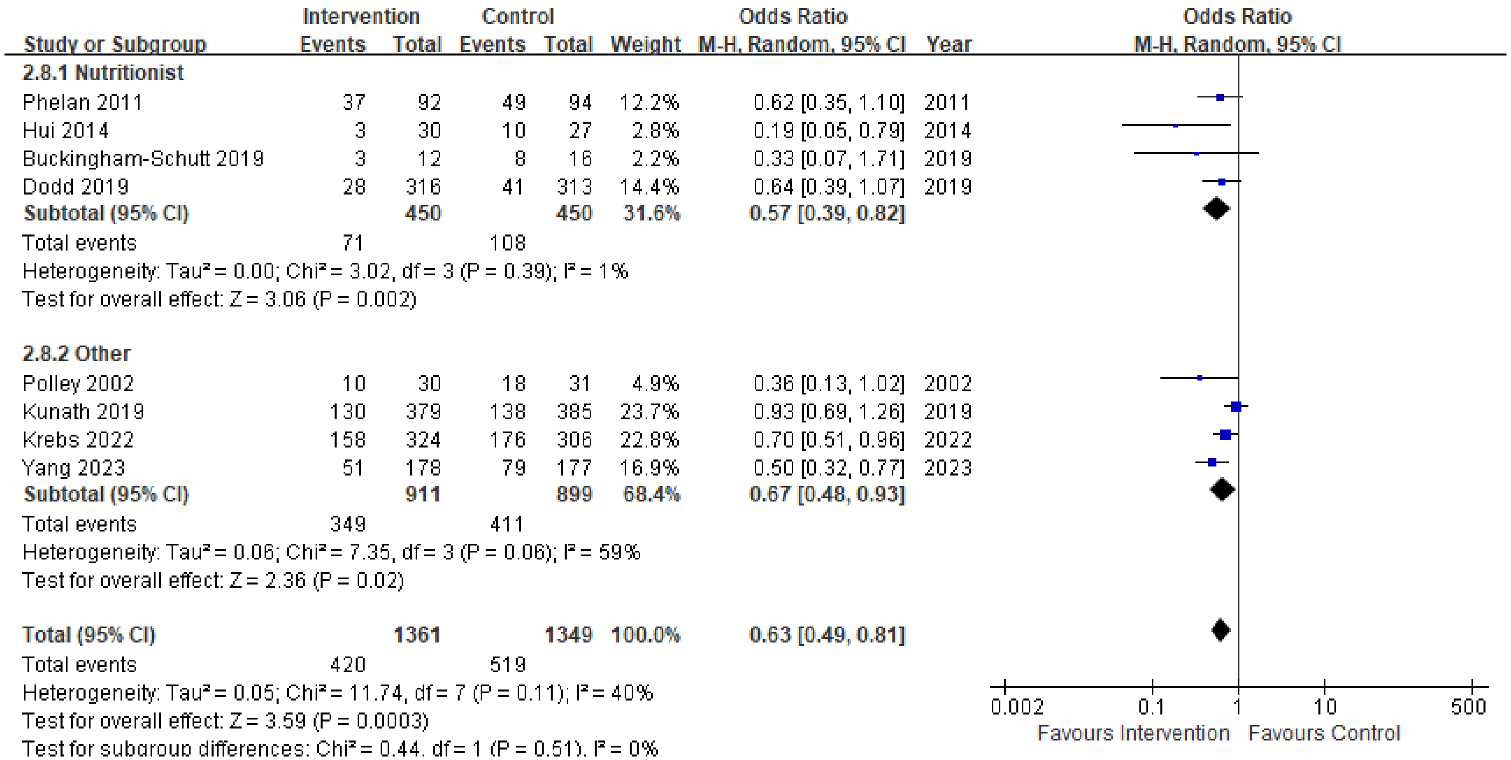

Figure S27. Forest plot of Effect of **Provider** in the physical activity component on EGWG

Study or Subgroup	Intervention Events	Intervention Total	Control Events	Control Total	Weight	Odds Ratio M-H, Random, 95% CI	Year
**2.19.1 Sports health professionals**							
Hui 2014	3	30	10	27	2.8%	0.19 [0.05, 0.79]	2014
**Subtotal (95% CI)**		**30**		**27**	**2.8%**	**0.19 [0.05, 0.79]**	
Total events	3		10				
Heterogeneity: Not applicable							
Test for overall effect: Z = 2.29 (P = 0.02)							
**2.19.2 Other**							
Polley 2002	10	30	18	31	4.9%	0.36 [0.13, 1.02]	2002
Phelan 2011	37	92	49	94	12.2%	0.62 [0.35, 1.10]	2011
Buckingham-Schutt 2019	3	12	8	16	2.2%	0.33 [0.07, 1.71]	2019
Kunath 2019	130	379	138	385	23.7%	0.93 [0.69, 1.26]	2019
Dodd 2019	28	316	41	313	14.4%	0.64 [0.39, 1.07]	2019
Krebs 2022	158	324	176	306	22.8%	0.70 [0.51, 0.96]	2022
Yang 2023	51	178	79	177	16.9%	0.50 [0.32, 0.77]	2023
**Subtotal (95% CI)**		**1331**		**1322**	**97.2%**	**0.67 [0.54, 0.83]**	
Total events	417		509				
Heterogeneity: Tau² = 0.02; Chi² = 8.55, df = 6 (P = 0.20); I² = 30%							
Test for overall effect: Z = 3.56 (P = 0.0004)							
**Total (95% CI)**		**1361**		**1349**	**100.0%**	**0.63 [0.49, 0.81]**	
Total events	420		519				
Heterogeneity: Tau² = 0.05; Chi² = 11.74, df = 7 (P = 0.11); I² = 40%							
Test for overall effect: Z = 3.59 (P = 0.0003)							
Test for subgroup differences: Chi² = 2.95, df = 1 (P = 0.09), I² = 66.1%							

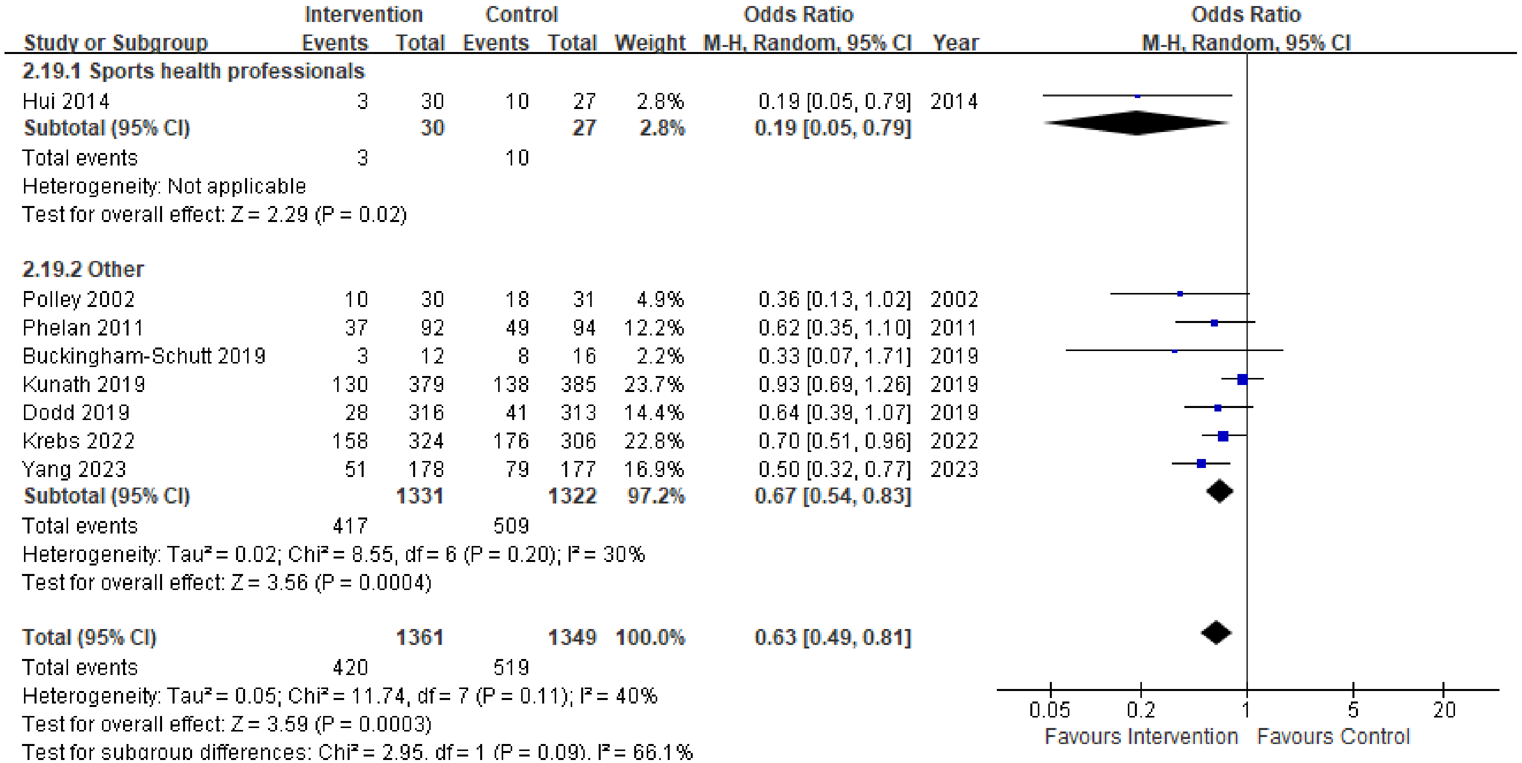

Figure S28. Forest plot of Effect of **Location** in the diet component on EGWG

Study or Subgroup	Intervention Events	Intervention Total	Control Events	Control Total	Weight	Odds Ratio M-H, Random, 95% CI	Year
**2.9.1 Healthcare Facility**							
Polley 2002	10	30	18	31	4.9%	0.36 [0.13, 1.02]	2002
Phelan 2011	37	92	49	94	12.2%	0.62 [0.35, 1.10]	2011
Kunath 2019	130	379	138	385	23.7%	0.93 [0.69, 1.26]	2019
Buckingham-Schutt 2019	3	12	8	16	2.2%	0.33 [0.07, 1.71]	2019
Dodd 2019	28	316	41	313	14.4%	0.64 [0.39, 1.07]	2019
Krebs 2022	158	324	176	306	22.8%	0.70 [0.51, 0.96]	2022
Yang 2023	51	178	79	177	16.9%	0.50 [0.32, 0.77]	2023
**Subtotal (95% CI)**		**1331**		**1322**	**97.2%**	**0.67 [0.54, 0.83]**	
Total events	417		509				
Heterogeneity: Tau² = 0.02; Chi² = 8.55, df = 6 (P = 0.20); I² = 30%							
Test for overall effect: Z = 3.56 (P = 0.0004)							
**2.9.2 Other**							
Hui 2014	3	30	10	27	2.8%	0.19 [0.05, 0.79]	2014
**Subtotal (95% CI)**		**30**		**27**	**2.8%**	**0.19 [0.05, 0.79]**	
Total events	3		10				
Heterogeneity: Not applicable							
Test for overall effect: Z = 2.29 (P = 0.02)							
**Total (95% CI)**		**1361**		**1349**	**100.0%**	**0.63 [0.49, 0.81]**	
Total events	420		519				
Heterogeneity: Tau² = 0.05; Chi² = 11.74, df = 7 (P = 0.11); I² = 40%							
Test for overall effect: Z = 3.59 (P = 0.0003)							
Test for subgroup differences: Chi² = 2.95, df = 1 (P = 0.09), I² = 66.1%							

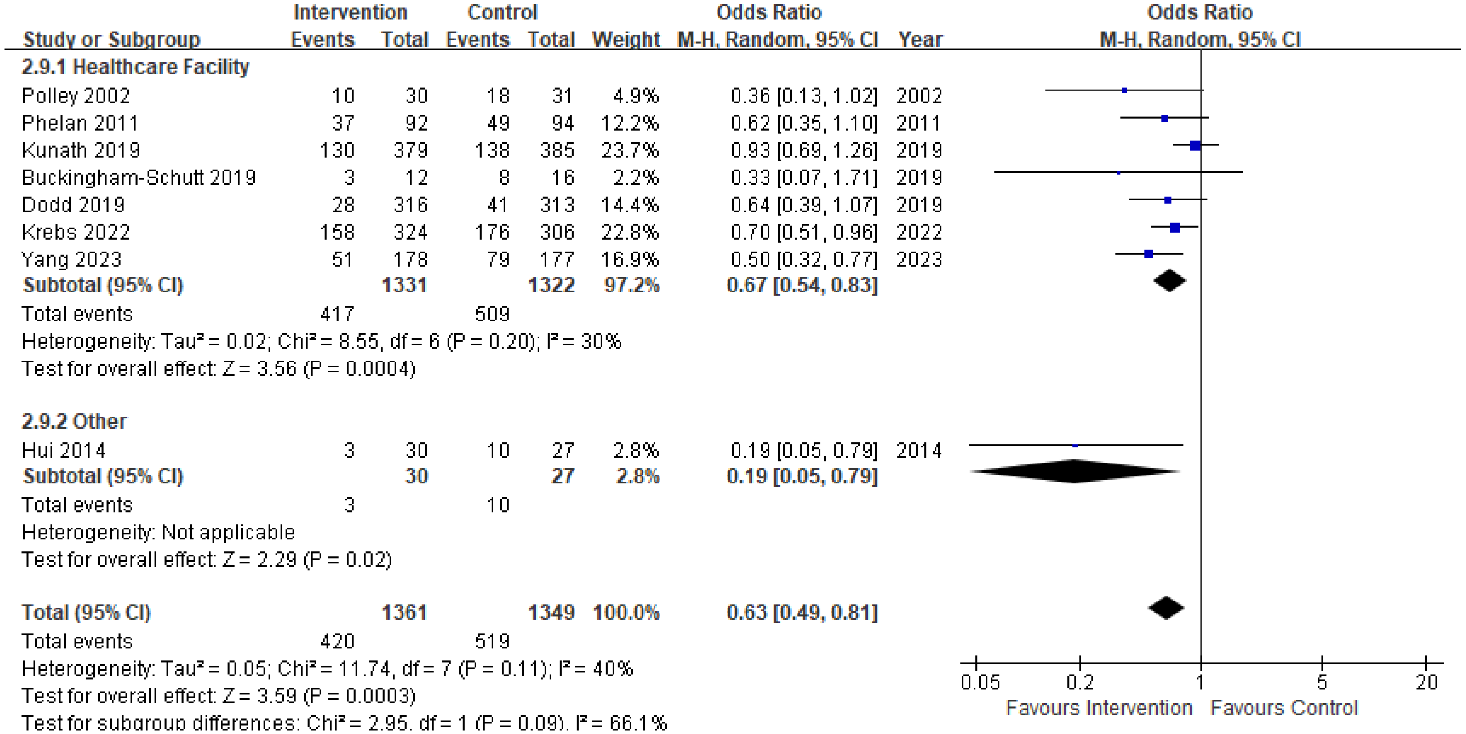

Figure S29. Forest plot of Effect of **Location** in the physical activity component on EGWG

Study or Subgroup	Intervention Events	Intervention Total	Control Events	Control Total	Weight	Odds Ratio M-H, Random, 95% CI	Year
**2.20.1 Healthcare Facility**							
Polley 2002	10	30	18	31	4.9%	0.36 [0.13, 1.02]	2002
Phelan 2011	37	92	49	94	12.2%	0.62 [0.35, 1.10]	2011
Buckingham-Schutt 2019	3	12	8	16	2.2%	0.33 [0.07, 1.71]	2019
Dodd 2019	28	316	41	313	14.4%	0.64 [0.39, 1.07]	2019
Kunath 2019	130	379	138	385	23.7%	0.93 [0.69, 1.26]	2019
Krebs 2022	158	324	176	306	22.8%	0.70 [0.51, 0.96]	2022
Yang 2023	51	178	79	177	16.9%	0.50 [0.32, 0.77]	2023
**Subtotal (95% CI)**		**1331**		**1322**	**97.2%**	**0.67 [0.54, 0.83]**	
Total events	417		509				
Heterogeneity: Tau² = 0.02; Chi² = 8.55, df = 6 (P = 0.20); I² = 30%							
Test for overall effect: Z = 3.56 (P = 0.0004)							
**2.20.2 Other**							
Hui 2014	3	30	10	27	2.8%	0.19 [0.05, 0.79]	2014
**Subtotal (95% CI)**		**30**		**27**	**2.8%**	**0.19 [0.05, 0.79]**	
Total events	3		10				
Heterogeneity: Not applicable							
Test for overall effect: Z = 2.29 (P = 0.02)							
**Total (95% CI)**		**1361**		**1349**	**100.0%**	**0.63 [0.49, 0.81]**	
Total events	420		519				
Heterogeneity: Tau² = 0.05; Chi² = 11.74, df = 7 (P = 0.11); I² = 40%							
Test for overall effect: Z = 3.59 (P = 0.0003)							
Test for subgroup differences: Chi² = 2.95, df = 1 (P = 0.09), I² = 66.1%							

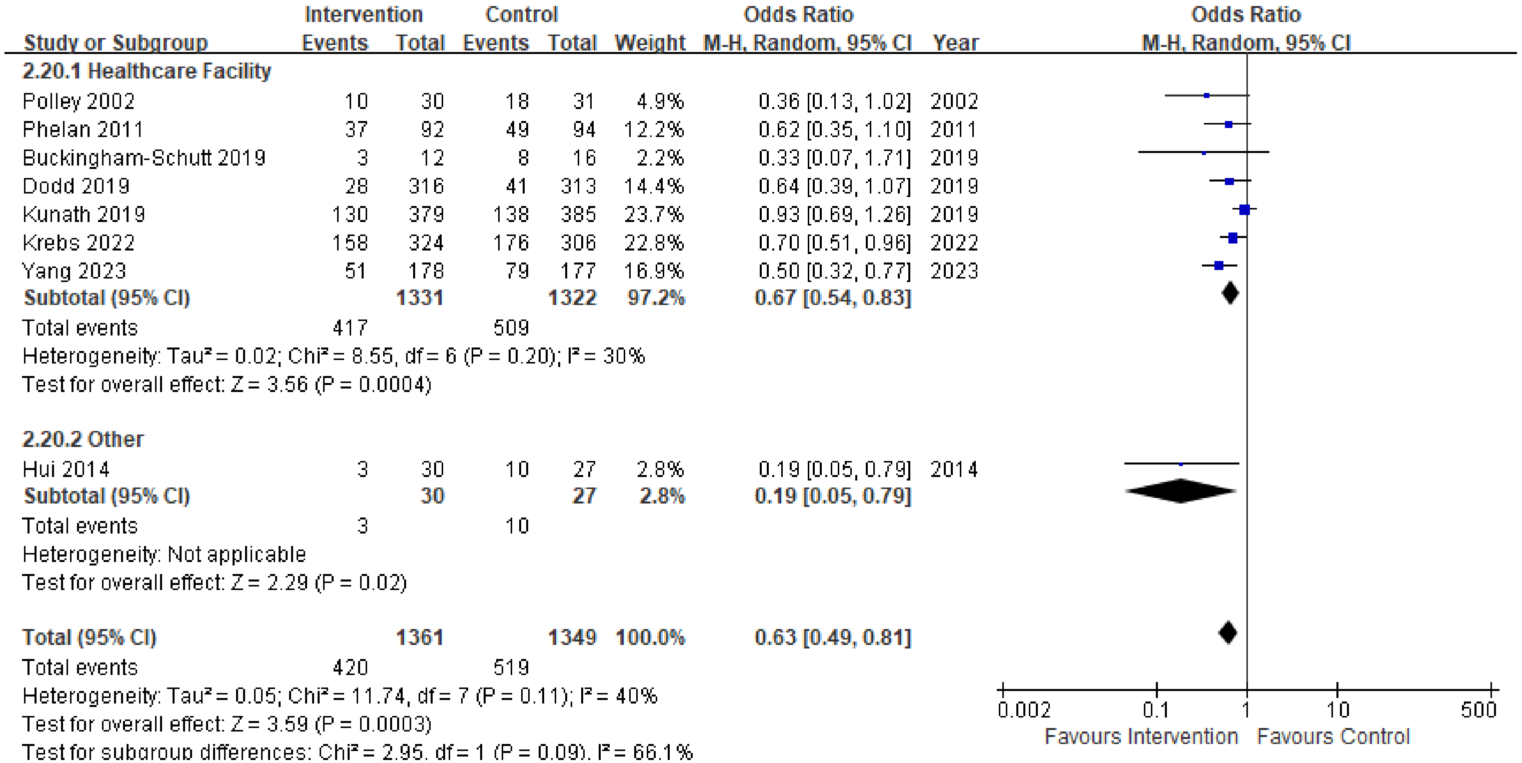

Figure S30. Forest plot of Effect of **Duration** in the diet component on EGWG

Study or Subgroup	Intervention Events	Intervention Total	Control Events	Control Total	Weight	Odds Ratio M-H, Random, 95% CI	Year
**2.10.1 Low:1-12**							
Hui 2014	3	30	10	27	2.8%	0.19 [0.05, 0.79]	2014
**Subtotal (95% CI)**		**30**		**27**	**2.8%**	**0.19 [0.05, 0.79]**	
Total events	3		10				
Heterogeneity: Not applicable							
Test for overall effect: Z = 2.29 (P = 0.02)							
**2.10.2 Moderate: 13-20**							
Polley 2002	10	30	18	31	4.9%	0.36 [0.13, 1.02]	2002
Dodd 2019	28	316	41	313	14.4%	0.64 [0.39, 1.07]	2019
**Subtotal (95% CI)**		**346**		**344**	**19.3%**	**0.58 [0.37, 0.91]**	
Total events	38		59				
Heterogeneity: $Tau^2 = 0.00$; $Chi^2 = 0.96$, df = 1 (P = 0.33); $I^2 = 0\%$							
Test for overall effect: Z = 2.36 (P = 0.02)							
**2.10.3 High: ⩾21**							
Phelan 2011	37	92	49	94	12.2%	0.62 [0.35, 1.10]	2011
Kunath 2019	130	379	138	385	23.7%	0.93 [0.69, 1.26]	2019
Buckingham-Schutt 2019	3	12	8	16	2.2%	0.33 [0.07, 1.71]	2019
Krebs 2022	158	324	176	306	22.8%	0.70 [0.51, 0.96]	2022
Yang 2023	51	178	79	177	16.9%	0.50 [0.32, 0.77]	2023
**Subtotal (95% CI)**		**985**		**978**	**77.8%**	**0.69 [0.53, 0.90]**	
Total events	379		450				
Heterogeneity: $Tau^2 = 0.03$; $Chi^2 = 6.79$, df = 4 (P = 0.15); $I^2 = 41\%$							
Test for overall effect: Z = 2.78 (P = 0.005)							
**Total (95% CI)**		**1361**		**1349**	**100.0%**	**0.63 [0.49, 0.81]**	
Total events	420		519				
Heterogeneity: $Tau^2 = 0.05$; $Chi^2 = 11.74$, df = 7 (P = 0.11); $I^2 = 40\%$							
Test for overall effect: Z = 3.59 (P = 0.0003)							
Test for subgroup differences: $Chi^2 = 3.30$, df = 2 (P = 0.19), $I^2 = 39.4\%$							

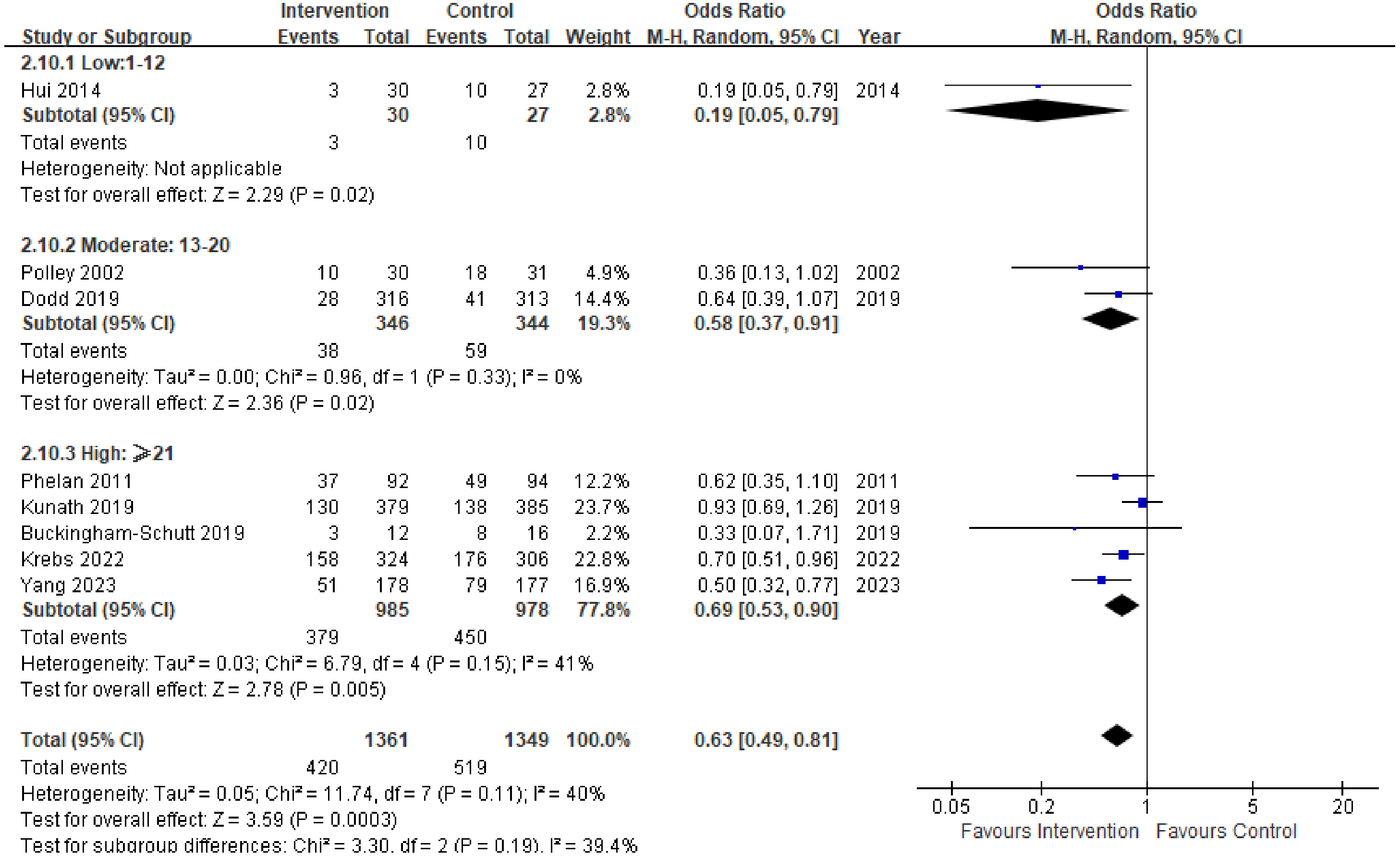

Figure S31. Forest plot of Effect of **Duration** in the physical activity component on EGWG

Study or Subgroup	Intervention Events	Intervention Total	Control Events	Control Total	Weight	Odds Ratio M-H, Random, 95% CI	Year
**2.21.1 Moderate: 13-20**							
Polley 2002	10	30	18	31	4.9%	0.36 [0.13, 1.02]	2002
Dodd 2019	28	316	41	313	14.4%	0.64 [0.39, 1.07]	2019
**Subtotal (95% CI)**		**346**		**344**	**19.3%**	**0.58 [0.37, 0.91]**	
Total events	38		59				
Heterogeneity: Tau² = 0.00; Chi² = 0.96, df = 1 (P = 0.33); I² = 0%							
Test for overall effect: Z = 2.36 (P = 0.02)							
**2.21.2 High: ≥21**							
Phelan 2011	37	92	49	94	12.2%	0.62 [0.35, 1.10]	2011
Hui 2014	3	30	10	27	2.8%	0.19 [0.05, 0.79]	2014
Buckingham-Schutt 2019	3	12	8	16	2.2%	0.33 [0.07, 1.71]	2019
Kunath 2019	130	379	138	385	23.7%	0.93 [0.69, 1.26]	2019
Krebs 2022	158	324	176	306	22.8%	0.70 [0.51, 0.96]	2022
Yang 2023	51	178	79	177	16.9%	0.50 [0.32, 0.77]	2023
**Subtotal (95% CI)**		**1015**		**1005**	**80.7%**	**0.64 [0.47, 0.87]**	
Total events	382		460				
Heterogeneity: Tau² = 0.06; Chi² = 10.13, df = 5 (P = 0.07); I² = 51%							
Test for overall effect: Z = 2.87 (P = 0.004)							
**Total (95% CI)**		**1361**		**1349**	**100.0%**	**0.63 [0.49, 0.81]**	
Total events	420		519				
Heterogeneity: Tau² = 0.05; Chi² = 11.74, df = 7 (P = 0.11); I² = 40%							
Test for overall effect: Z = 3.59 (P = 0.0003)							
Test for subgroup differences: Chi² = 0.15, df = 1 (P = 0.70), I² = 0%							

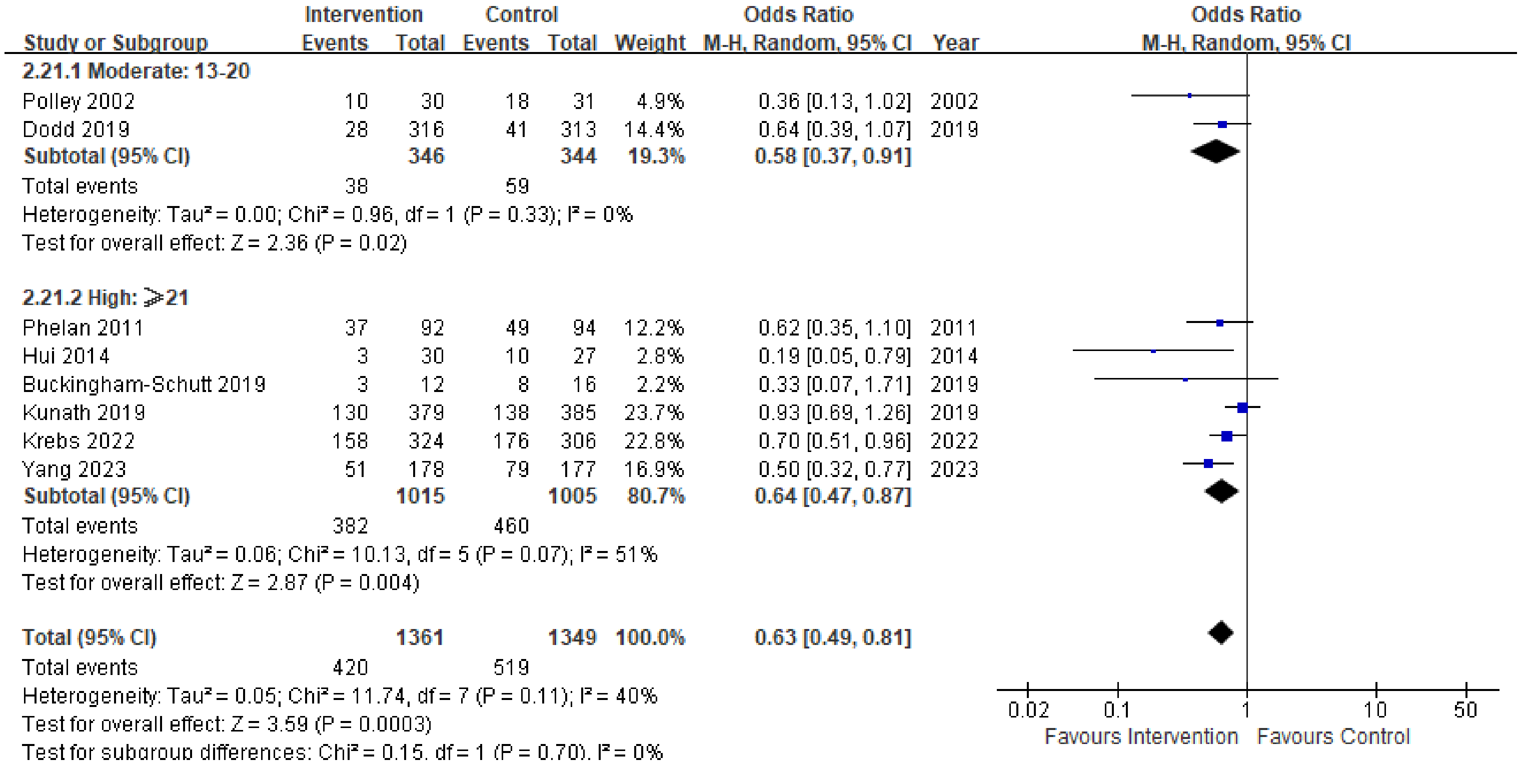

Figure S32. Forest plot of Effect of **No. interventions** in the diet component on EGWG

Study or Subgroup	Intervention Events	Intervention Total	Control Events	Control Total	Weight	Odds Ratio M-H, Random, 95% CI	Year
**2.11.1 Low:1-5**							
Hui 2014	3	30	10	27	2.8%	0.19 [0.05, 0.79]	2014
Kunath 2019	130	379	138	385	23.7%	0.93 [0.69, 1.26]	2019
Yang 2023	51	178	79	177	16.9%	0.50 [0.32, 0.77]	2023
**Subtotal (95% CI)**		**587**		**589**	**43.5%**	**0.57 [0.29, 1.11]**	
Total events	184		227				
Heterogeneity: Tau² = 0.23; Chi² = 9.00, df = 2 (P = 0.01); I² = 78%							
Test for overall effect: Z = 1.66 (P = 0.10)							
**2.11.2 Moderate: 6-20**							
Polley 2002	10	30	18	31	4.9%	0.36 [0.13, 1.02]	2002
Phelan 2011	37	92	49	94	12.2%	0.62 [0.35, 1.10]	2011
Dodd 2019	28	316	41	313	14.4%	0.64 [0.39, 1.07]	2019
Buckingham-Schutt 2019	3	12	8	16	2.2%	0.33 [0.07, 1.71]	2019
Krebs 2022	158	324	176	306	22.8%	0.70 [0.51, 0.96]	2022
**Subtotal (95% CI)**		**774**		**760**	**56.5%**	**0.64 [0.51, 0.81]**	
Total events	236		292				
Heterogeneity: Tau² = 0.00; Chi² = 2.13, df = 4 (P = 0.71); I² = 0%							
Test for overall effect: Z = 3.69 (P = 0.0002)							
**Total (95% CI)**		**1361**		**1349**	**100.0%**	**0.63 [0.49, 0.81]**	
Total events	420		519				
Heterogeneity: Tau² = 0.05; Chi² = 11.74, df = 7 (P = 0.11); I² = 40%							
Test for overall effect: Z = 3.59 (P = 0.0003)							
Test for subgroup differences: Chi² = 0.11, df = 1 (P = 0.74), I² = 0%							

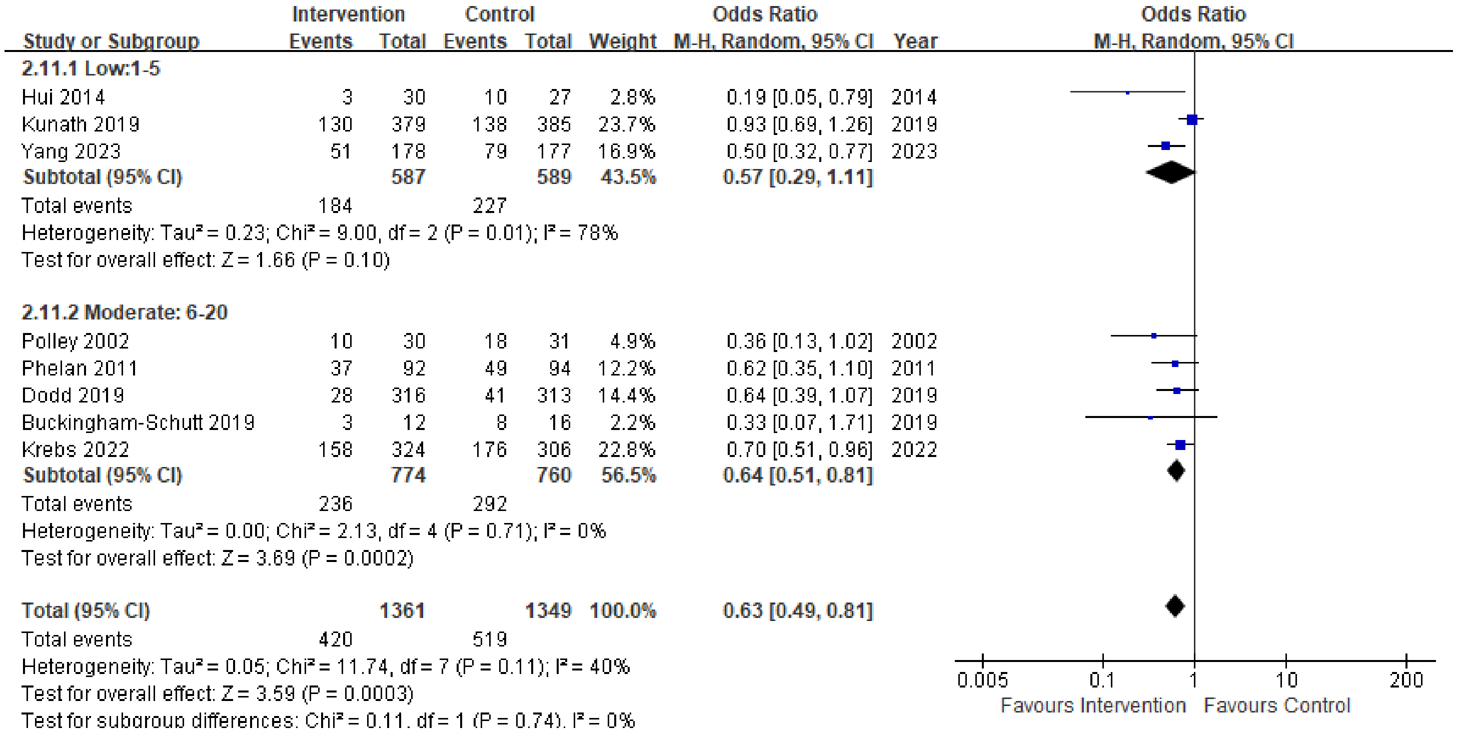

Figure S33. Forest plot of Effect of **No. interventions** in the physical activity component on EGWG

Study or Subgroup	Intervention Events	Intervention Total	Control Events	Control Total	Weight	Odds Ratio M-H, Random, 95% CI	Year
**2.22.1 Moderate: 6-20**							
Polley 2002	10	30	18	31	4.9%	0.36 [0.13, 1.02]	2002
Phelan 2011	37	92	49	94	12.2%	0.62 [0.35, 1.10]	2011
Dodd 2019	28	316	41	313	14.4%	0.64 [0.39, 1.07]	2019
Krebs 2022	158	324	176	306	22.8%	0.70 [0.51, 0.96]	2022
**Subtotal (95% CI)**		**762**		**744**	**54.3%**	**0.65 [0.52, 0.83]**	
Total events	233		284				
Heterogeneity: Tau² = 0.00; Chi² = 1.49, df = 3 (P = 0.68); I² = 0%							
Test for overall effect: Z = 3.54 (P = 0.0004)							
**2.22.2 High: ≥21**							
Hui 2014	3	30	10	27	2.8%	0.19 [0.05, 0.79]	2014
Buckingham-Schutt 2019	3	12	8	16	2.2%	0.33 [0.07, 1.71]	2019
Kunath 2019	130	379	138	385	23.7%	0.93 [0.69, 1.26]	2019
Yang 2023	51	178	79	177	16.9%	0.50 [0.32, 0.77]	2023
**Subtotal (95% CI)**		**599**		**605**	**45.7%**	**0.54 [0.30, 1.00]**	
Total events	187		235				
Heterogeneity: Tau² = 0.21; Chi² = 9.89, df = 3 (P = 0.02); I² = 70%							
Test for overall effect: Z = 1.97 (P = 0.05)							
**Total (95% CI)**		**1361**		**1349**	**100.0%**	**0.63 [0.49, 0.81]**	
Total events	420		519				
Heterogeneity: Tau² = 0.05; Chi² = 11.74, df = 7 (P = 0.11); I² = 40%							
Test for overall effect: Z = 3.59 (P = 0.0003)							
Test for subgroup differences: Chi² = 0.30, df = 1 (P = 0.58), I² = 0%							

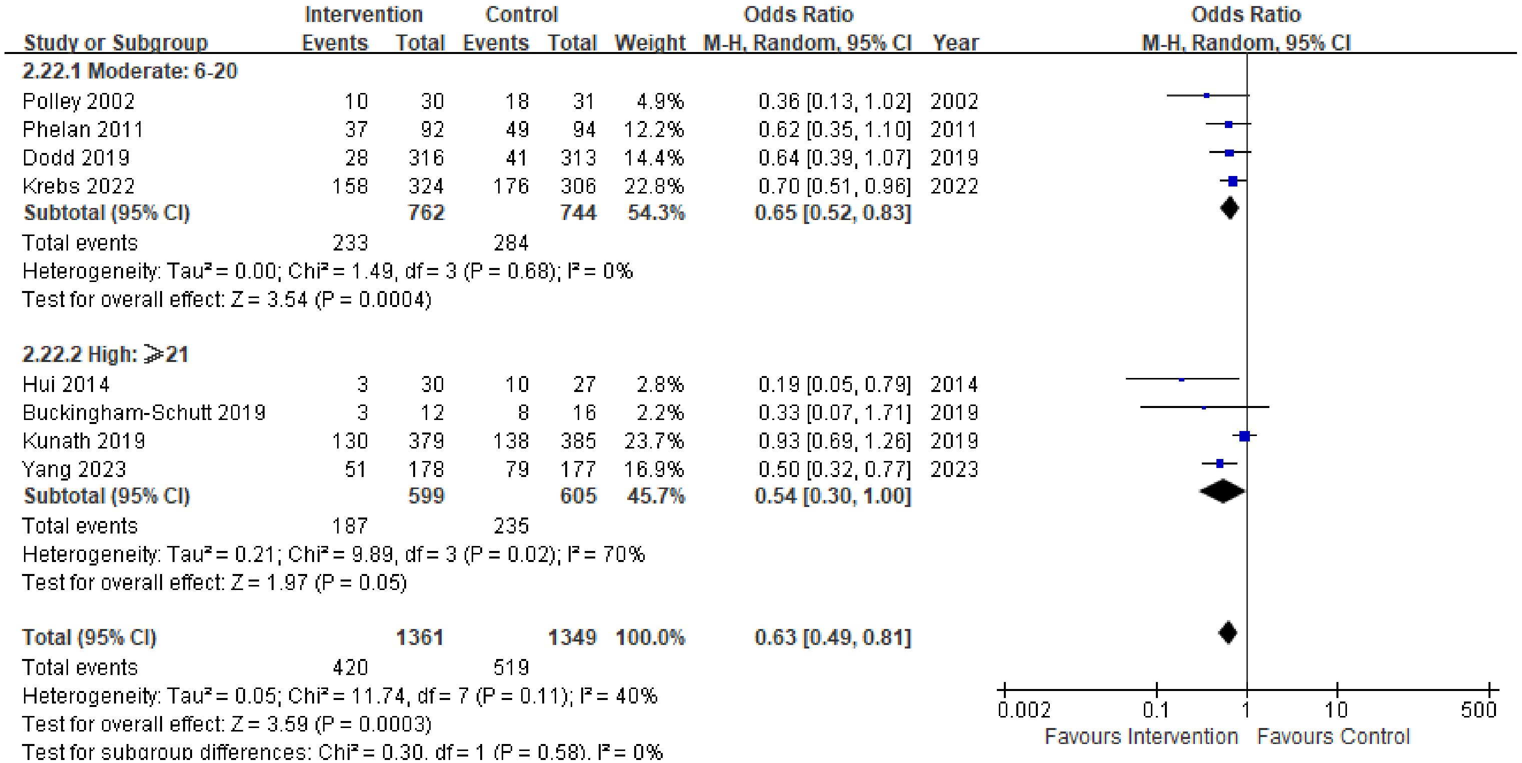

Figure S34. Forest plot of Effect of **Tailoring** in the physical activity component on EGWG

Study or Subgroup	Intervention Events	Intervention Total	Control Events	Control Total	Weight	Odds Ratio M-H, Random, 95% CI	Year
**2.23.1 With tailoring**							
Polley 2002	10	30	18	31	4.9%	0.36 [0.13, 1.02]	2002
Kunath 2019	130	379	138	385	23.7%	0.93 [0.69, 1.26]	2019
Dodd 2019	28	316	41	313	14.4%	0.64 [0.39, 1.07]	2019
Krebs 2022	158	324	176	306	22.8%	0.70 [0.51, 0.96]	2022
Yang 2023	51	178	79	177	16.9%	0.50 [0.32, 0.77]	2023
**Subtotal (95% CI)**		**1227**		**1212**	**82.8%**	**0.68 [0.52, 0.88]**	
Total events	377		452				
Heterogeneity: Tau² = 0.04; Chi² = 7.53, df = 4 (P = 0.11); I² = 47%							
Test for overall effect: Z = 2.89 (P = 0.004)							
**2.23.2 Without tailoring**							
Phelan 2011	37	92	49	94	12.2%	0.62 [0.35, 1.10]	2011
Hui 2014	3	30	10	27	2.8%	0.19 [0.05, 0.79]	2014
Buckingham-Schutt 2019	3	12	8	16	2.2%	0.33 [0.07, 1.71]	2019
**Subtotal (95% CI)**		**134**		**137**	**17.2%**	**0.45 [0.23, 0.88]**	
Total events	43		67				
Heterogeneity: Tau² = 0.10; Chi² = 2.54, df = 2 (P = 0.28); I² = 21%							
Test for overall effect: Z = 2.31 (P = 0.02)							
**Total (95% CI)**		**1361**		**1349**	**100.0%**	**0.63 [0.49, 0.81]**	
Total events	420		519				
Heterogeneity: Tau² = 0.05; Chi² = 11.74, df = 7 (P = 0.11); I² = 40%							
Test for overall effect: Z = 3.59 (P = 0.0003)							
Test for subgroup differences: Chi² = 1.22, df = 1 (P = 0.27), I² = 18.0%							

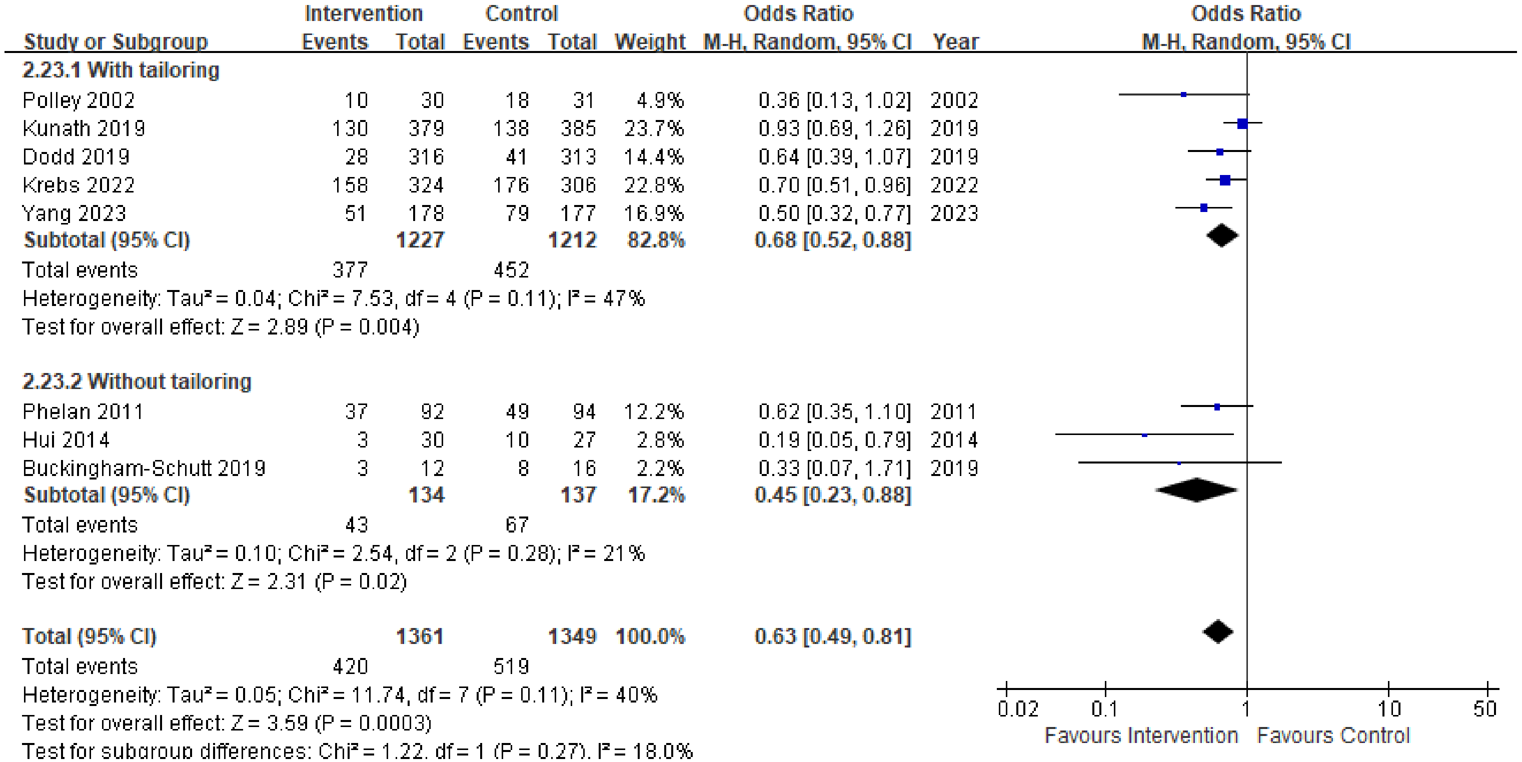

Figure S35. Forest plot of Effect of **Resource** in the diet component on EGWG

Study or Subgroup	Intervention Events	Intervention Total	Control Events	Control Total	Weight	Odds Ratio M-H, Random, 95% CI	Year
**2.4.1 Other resource**							
Polley 2002	10	30	18	31	4.9%	0.36 [0.13, 1.02]	2002
Hui 2014	3	30	10	27	2.8%	0.19 [0.05, 0.79]	2014
Dodd 2019	28	316	41	313	14.4%	0.64 [0.39, 1.07]	2019
Krebs 2022	158	324	176	306	22.8%	0.70 [0.51, 0.96]	2022
Yang 2023	51	178	79	177	16.9%	0.50 [0.32, 0.77]	2023
**Subtotal (95% CI)**		**878**		**854**	**61.9%**	**0.57 [0.44, 0.76]**	
Total events	250		324				
Heterogeneity: Tau² = 0.02; Chi² = 5.18, df = 4 (P = 0.27); I² = 23%							
Test for overall effect: Z = 3.95 (P < 0.0001)							
**2.4.2 Combination**							
Phelan 2011	37	92	49	94	12.2%	0.62 [0.35, 1.10]	2011
Buckingham-Schutt 2019	3	12	8	16	2.2%	0.33 [0.07, 1.71]	2019
Kunath 2019	130	379	138	385	23.7%	0.93 [0.69, 1.26]	2019
**Subtotal (95% CI)**		**483**		**495**	**38.1%**	**0.78 [0.53, 1.15]**	
Total events	170		195				
Heterogeneity: Tau² = 0.04; Chi² = 2.80, df = 2 (P = 0.25); I² = 29%							
Test for overall effect: Z = 1.27 (P = 0.20)							
**Total (95% CI)**		**1361**		**1349**	**100.0%**	**0.63 [0.49, 0.81]**	
Total events	420		519				
Heterogeneity: Tau² = 0.05; Chi² = 11.74, df = 7 (P = 0.11); I² = 40%							
Test for overall effect: Z = 3.59 (P = 0.0003)							
Test for subgroup differences: Chi² = 1.55, df = 1 (P = 0.21), I² = 35.5%							

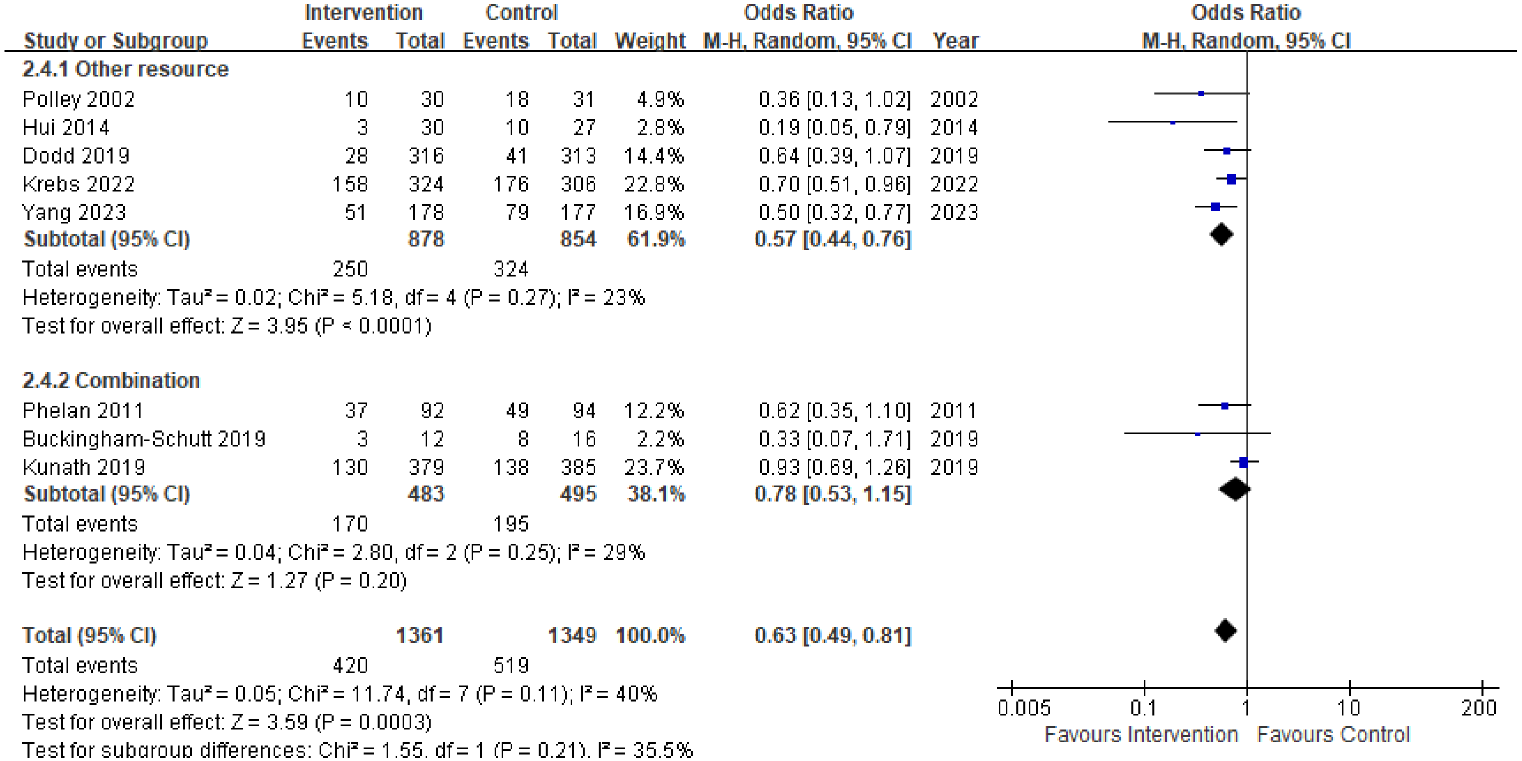

Figure S36. Forest plot of Effect of **Resource** in the physical activity component on EGWG

Study or Subgroup	Intervention Events	Intervention Total	Control Events	Control Total	Weight	Odds Ratio M-H, Random, 95% CI	Year
**2.15.1 Other resource**							
Polley 2002	10	30	18	31	4.9%	0.36 [0.13, 1.02]	2002
Hui 2014	3	30	10	27	2.8%	0.19 [0.05, 0.79]	2014
Dodd 2019	28	316	41	313	14.4%	0.64 [0.39, 1.07]	2019
Krebs 2022	158	324	176	306	22.8%	0.70 [0.51, 0.96]	2022
Yang 2023	51	178	79	177	16.9%	0.50 [0.32, 0.77]	2023
**Subtotal (95% CI)**		**878**		**854**	**61.9%**	**0.57 [0.44, 0.76]**	
Total events	250		324				
Heterogeneity: Tau² = 0.02; Chi² = 5.18, df = 4 (P = 0.27); I² = 23%							
Test for overall effect: Z = 3.95 (P < 0.0001)							
**2.15.2 Combination**							
Phelan 2011	37	92	49	94	12.2%	0.62 [0.35, 1.10]	2011
Kunath 2019	130	379	138	385	23.7%	0.93 [0.69, 1.26]	2019
Buckingham-Schutt 2019	3	12	8	16	2.2%	0.33 [0.07, 1.71]	2019
**Subtotal (95% CI)**		**483**		**495**	**38.1%**	**0.78 [0.53, 1.15]**	
Total events	170		195				
Heterogeneity: Tau² = 0.04; Chi² = 2.80, df = 2 (P = 0.25); I² = 29%							
Test for overall effect: Z = 1.27 (P = 0.20)							
**Total (95% CI)**		**1361**		**1349**	**100.0%**	**0.63 [0.49, 0.81]**	
Total events	420		519				
Heterogeneity: Tau² = 0.05; Chi² = 11.74, df = 7 (P = 0.11); I² = 40%							
Test for overall effect: Z = 3.59 (P = 0.0003)							
Test for subgroup differences: Chi² = 1.55, df = 1 (P = 0.21), I² = 35.5%							

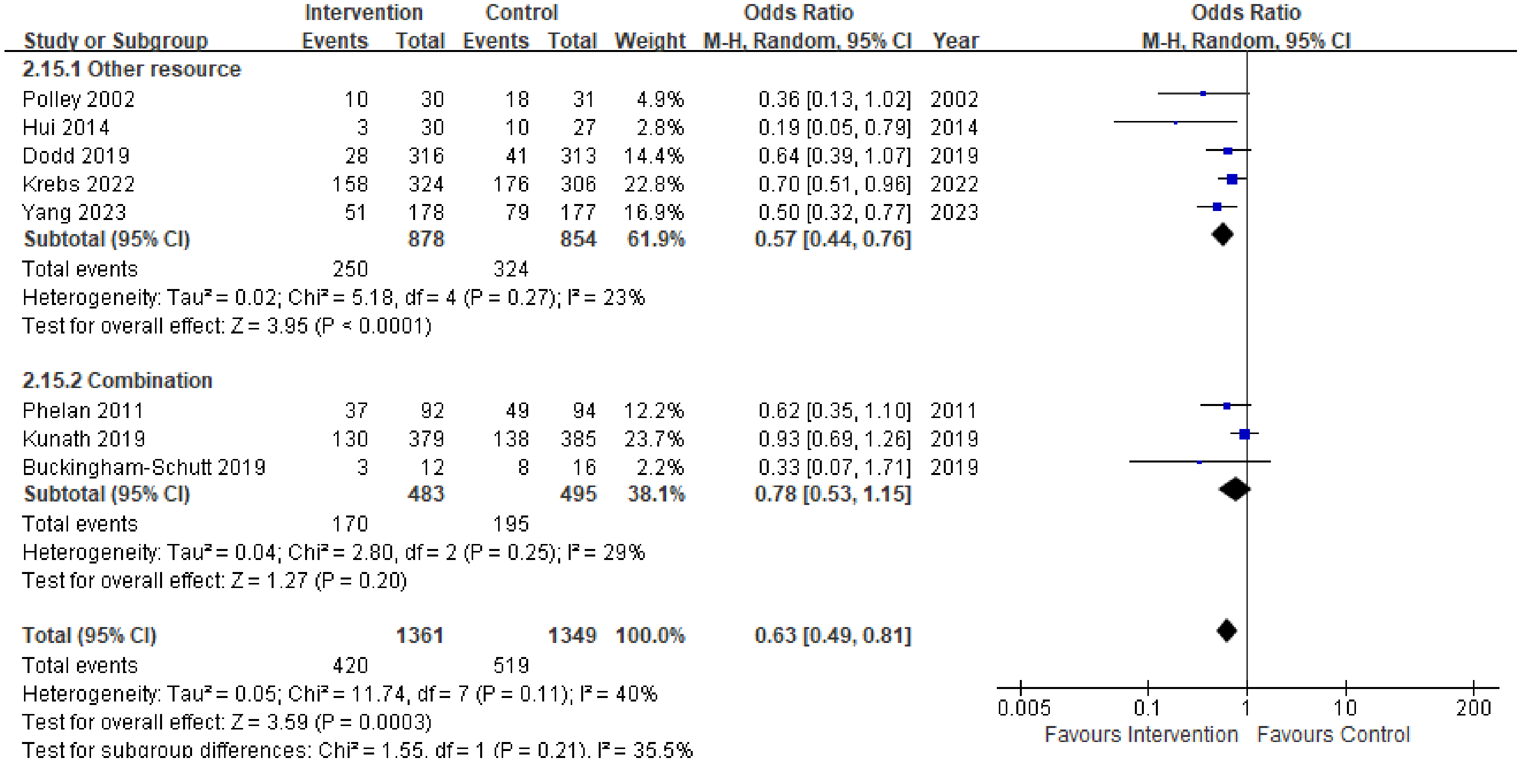

4. Funnel plot of total gestational weight gain

Figure S37. Funnel plot of total GWG (*n*=10)

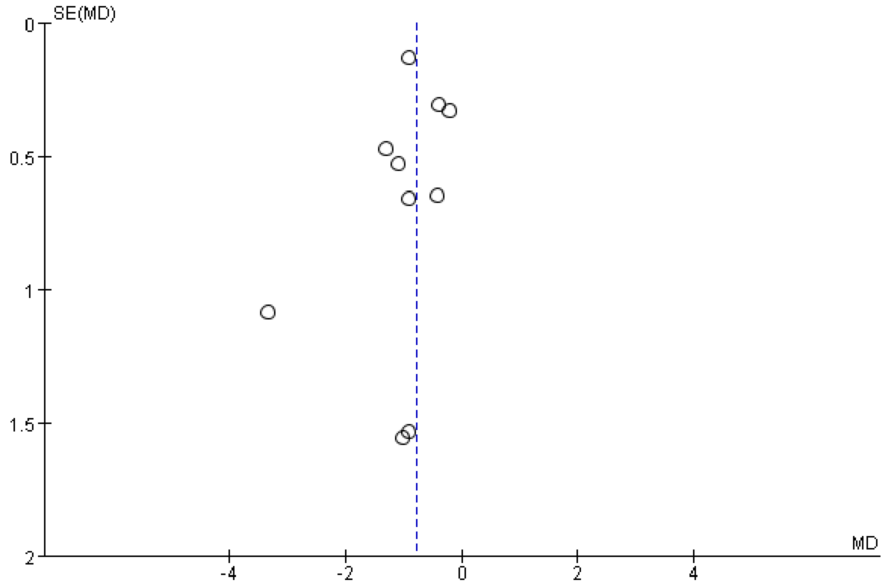

Supplement: Supplementary file 1 [file healthcare-14-01035-s001.zip › Supplementary File S6. Supplementary figures.pdf]
