# Supplementary material for: Effects of Combined Diet and Physical Activity on Gestational Weight Gain in Low-Risk Pregnant Women Based on the TIDieR Checklist: A Systematic Review and Meta-Analysis
Source: Healthcare (Basel). 2026 Apr 14;14(8):1035. doi: 10.3390/healthcare14081035 (PMC13115787; doi:10.3390/healthcare14081035)
Supplement: Supplementary file 1 [file healthcare-14-01035-s001.zip › Supplementary File S7. Summary of the results of sensitivity analyses.pdf]

Supplementary File S7. Summary of the results of sensitivity analyses

Summary of the results of sensitivity analyses		
**Outcomes**	**Removed study**	**Statistical results**
1. Total gestational weight gain (kg)<br>MD [95% CI]: −0.78 [−1.12, −0.44], *P*<0.00001	Polley 2002	MD [95% CI]: −0.79 [−1.15, −0.43], *P*<0.0001
	Phelan 2011	MD [95% CI]: −0.78 [−1.16, −0.41], *P*<0.0001
	Hui 2014	MD [95% CI]: −0.78 [−0.99, −0.57], *P*<0.00001
	Sagedal 2016	MD [95% CI]: −0.76 [−1.14, −0.38], *P*<0.0001
	Buckingham-Schutt 2019	MD [95% CI]: −0.79 [−1.15, −0.43], *P*<0.0001
	Kunath 2019	MD [95% CI]: −0.88 [−1.18, −0.57], *P*<0.00001
	Dodd 2019	MD [95% CI]: −0.87 [−1.24, −0.50], *P*<0.00001
	Atkinson 2022	MD [95% CI]: −0.81 [−1.18, −0.45], *P*<0.0001
	Krebs 2022	MD [95% CI]: −0.77 [−1.22, −0.31], *P*=0.0009
	Yang 2023	MD [95% CI]: −0.73 [−1.09, −0.36], *P*<0.0001
2. The incidence of EGWG (%)<br>OR [95% CI]: 0.63 [0.49, 0.81], *P*=0.0003	Polley 2002	OR [95% CI]: 0.65 [0.51, 0.84], *P*=0.0009
	Phelan 2011	OR [95% CI]: 0.62 [0.47, 0.83], *P*=0.001
	Hui 2014	OR [95% CI]: 0.67 [0.54, 0.83], *P*=0.0004
	Buckingham-Schutt 2019	OR [95% CI]: 0.64 [0.50, 0.83], *P*=0.0007
	Kunath 2019	OR [95% CI]: 0.59 [0.48, 0.73], *P*<0.00001
	Dodd 2019	OR [95% CI]: 0.62 [0.46, 0.83], *P*=0.001
	Krebs 2022	OR [95% CI]: 0.59 [0.42, 0.82], *P*=0.002
	Yang 2023	OR [95% CI]: 0.67 [0.51, 0.87], *P*=0.003
3. The proportion of women with total GWG within the IOM-recommended range (%)<br>OR [95% CI]: 1.38 [1.05, 1.80], P=0.02	Polley 2002	OR [95% CI]: 1.36 [1.03, 1.80], *P*=0.03
	Phelan 2011	OR [95% CI]: 1.33 [0.98, 1.81], *P*=0.06
	Buckingham-Schutt 2019	OR [95% CI]: 1.34 [1.02, 1.76], *P*=0.04
	Dodd 2019	OR [95% CI]: 1.70 [1.04, 2.78], *P*=0.03
4. The proportion of women with total GWG below the IOM-recommended range (%)<br>OR [95% CI]: 0.00 [−0.06, 0.06], *P*=0.92	Polley 2002	OR [95% CI]: 0.01 [−0.07, 0.06], *P*=0.83
	Phelan 2011	OR [95% CI]: 0.00 [−0.07, 0.07], *P*=0.99
	Buckingham-Schutt 2019	OR [95% CI]: 0.00 [−0.06, 0.07], *P*=0.89
	Dodd 2019	OR [95% CI]: 0.04 [−0.05, 0.12], *P*=0.40
