# Supplementary material for: Effects of Combined Diet and Physical Activity on Gestational Weight Gain in Low-Risk Pregnant Women Based on the TIDieR Checklist: A Systematic Review and Meta-Analysis
Source: Healthcare (Basel). 2026 Apr 14;14(8):1035. doi: 10.3390/healthcare14081035 (PMC13115787; doi:10.3390/healthcare14081035)
Supplement: Supplementary file 1 [file healthcare-14-01035-s001.zip › Supplementary File S8. GRADE evidence of outcomes.pdf]

Supplementary File S8. GRADE evidence of outcomes

Certainty assessment							№ of patients		Effect		Certainty	Importance
№ of studies	Study design	Risk of bias	Inconsistency	Indirectness	Imprecision	Other considerations	Intervention	Control	Relative (95% CI)	Absolute (95% CI)		
**1. Total gestational weight gain (kg)**												
10	randomised trials	serious	not serious	not serious	not serious	none	1608	1616	-	MD **0.78 lower** (1.12 lower to 0.44 lower)	⨁⨁⨁◯ Moderate	-
**2. The incidence of EGWG (%)**												
8	randomised trials	serious	not serious	not serious	not serious	none	420/1316 (30.9%)	519/1349 (38.5%)	**OR 0.63** (0.49 to 0.81)	**102 fewer per 1,000** (from 150 fewer to 49 fewer)	⨁⨁⨁◯ Moderate	-
**3. The proportion of women with total GWG within the IOM-recommended range (%)**												
4	randomised trials	serious	not serious	not serious	not serious	none	189/450 (42.0%)	157/454 (34.6%)	**OR 1.38** (1.05 to 1.80)	**76 more per 1,000** (from 11 more to 142 more)	⨁⨁⨁◯ Moderate	-
**4. The proportion of women with total GWG below the IOM-recommended range (%)**												
4	randomised trials	serious	not serious	not serious	serious	none	183/450 (40.7%)	181/454 (39.9%)	**OR 0.00** (-0.06 to 0.06)	**-- per 1,000** (from 440 fewer to 360 fewer)	⨁⨁◯◯ Low	-

CI: confidence interval; **MD:** mean difference; **OR:** odds ratio
